# Mallory-Type Reactivity of 1,2-Dihydroazaborinines: 6π-Electrocyclization-[1,5]‑H Shift Cascade toward BN-Doped Polycyclic Frameworks

**DOI:** 10.1021/acs.orglett.5c04676

**Published:** 2025-12-31

**Authors:** Sonja M. Biebl, Robert C. Richter, Markus Ströbele, Ivana Fleischer, Holger F. Bettinger

**Affiliations:** † Institut für Organische Chemie, 9188 Eberhard Karls Universität Tübingen, Auf der Morgenstelle 18, 72076 Tübingen, Germany; ‡ Institut für Anorganische Chemie, Eberhard Karls Universität Tübingen, Auf der Morgenstelle 18, 72076 Tübingen, Germany

## Abstract

1,2-Dihydro-1,2-azaborinines exhibit highly selective substitution-dependent photochemistry, yielding either Dewar or benzvalene isomers. Through targeted substitution, an o-BN-terphenyl framework is constructed, which undergoes 6π-electrocyclization upon irradiation. The resulting BN-polyenes rearomatize spontaneously via a [1,5]-hydrogen shift. Under oxidative conditions, an unprecedented BN incorporated triphenylene results. By rationally tuning substitution and reaction conditions, this pathway is favored over Dewar isomer formation, offering selective routes to BN-doped polycyclic frameworks from multiphotoresponsive precursors.

## Graphical abstract

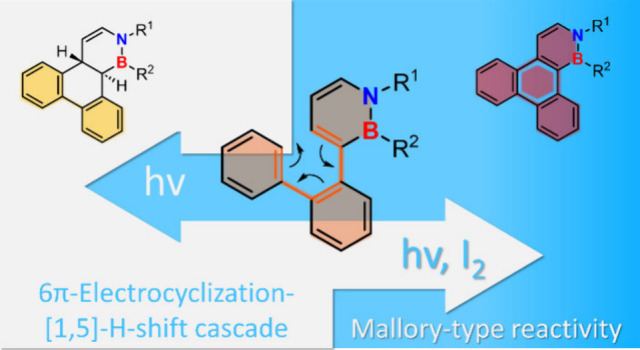

Concerted photochemical reactions provide a powerful complement to their thermal, ground-state counterparts and are gaining increasing significance in synthetic organic chemistry.^1^ Among these photochemical processes, the 6π-electrocyclization of 1,3,5-hexatriene systems continues to attract considerable attention due to its synthetic utility.^2,3^ One prominent example is the photocyclization of diarylethenes, which proceeds through a disrotatory 6π-electrocyclization pathway under UV irradiation.^4^ This transformation yields highly reactive polyenes, which then undergo oxidation (Mallory reaction),^4,5^ elimination,^1,6–8^ or subsequent rearrangements^9^ to restore the aromaticity of the system. Through modifying the reactants or varying the reaction conditions, in addition to phenanthrenes, other complex functionalized products^10^ such as naphthalenes^11^ or benzannulated heterocycles^12–14^ can be generated with high selectivity. However, the outcome of such photochemical reactions becomes challenging to predict when a molecule contains multiple photoactive moieties, each capable of undergoing competing photochemical transformations.^15^ Although promising in theory, efficient multi responsive materials remain scarce,^11,16,17^ as product mixtures cause reduced yields and tedious separation procedures and necessitate adaptation of the reaction conditions to favor selectivity toward one of the competing pathways.^11^

**Scheme 1:**
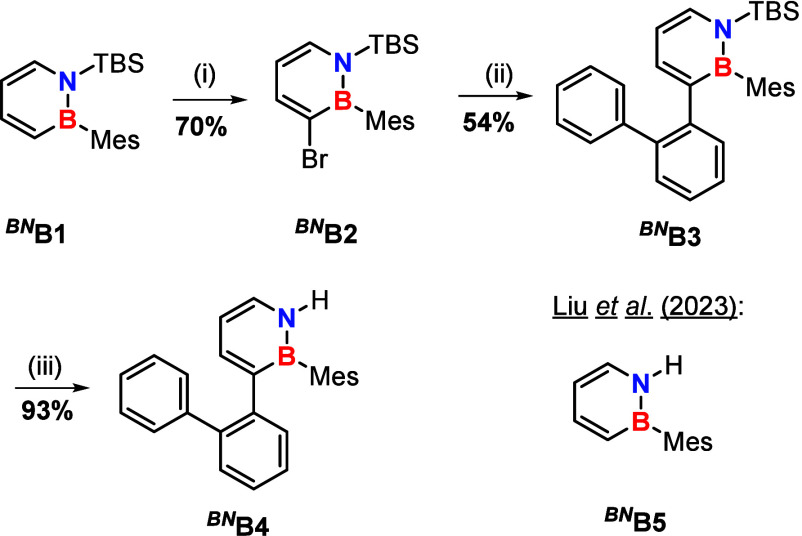
Preparation of the Compounds *^BN^* **B3** and *^BN^* **B4** Starting from Literature-Known Precursors^a^

a (i) Br_2_ (1 equiv), DCM, 0 °C to rt; (ii) Pd_2_dba_3_ (2 mol %), *S*-BIDIME (6 mol %), 2-biphenylboronic acid (1.3 equiv), Cs_2_CO_3_ (2 equiv), toluene/water 5/1, 50 °C, 20 h; (iii) TBAF (1.3 equiv), THF, rt, 1 h; *^BN^* **B5** was synthesized according to Liu et al.^27^

Herein, we describe the synthesis and photochemical behavior of 3-(2-biphenylyl)-1,2-dihydro-1,2-azaborinine derivatives (Figure 1). Upon UV irradiation substitution-dependent and highly selective photoisomerizations have been observed in the past for this substance class (*^BN^* **B**, Scheme 1).^18–22^ Species bearing substituents at nitrogen, boron, and the carbon atom adjacent to boron (C3) undergo a selective photoisomerization to the corresponding Dewar isomer (*^BN^* **D**, 2-aza-3-borabicyclo[2.2.0]­hex-5-ene).^18,19,23,24^ In contrast, an aryl substituent at the carbon atom *para* to boron (C5) directs the reaction toward isomerization to the benzvalene isomer (3-aza-4-boratricyclo­[3.1.0.0^2.6^]-hexane).^20^ With respect to thermal isomerizations, Liu and co-workers reported a Claisen rearrangement occurring in the presence of suitable substitution patterns (Figure 1).^25^ The presence of the 2-biphenylyl substituent also enables a photochemical 6π-electrocyclization involving one bond from each aromatic ring (Scheme 2). Since this process leads to the loss of aromaticity in all three rings, it appears to be less favorable. However, a subsequent thermal [1,5]-hydrogen shift confines the loss to the heterocyclic ring.^26^ Through targeted structural modification of the precursor compound, exclusively the 6π-electrocyclization-[1,5]-hydrogen-shift cascade is observed, providing access to previously unreported BN-triphenylenes with a nonfused BN bond.

**Figure 1:**
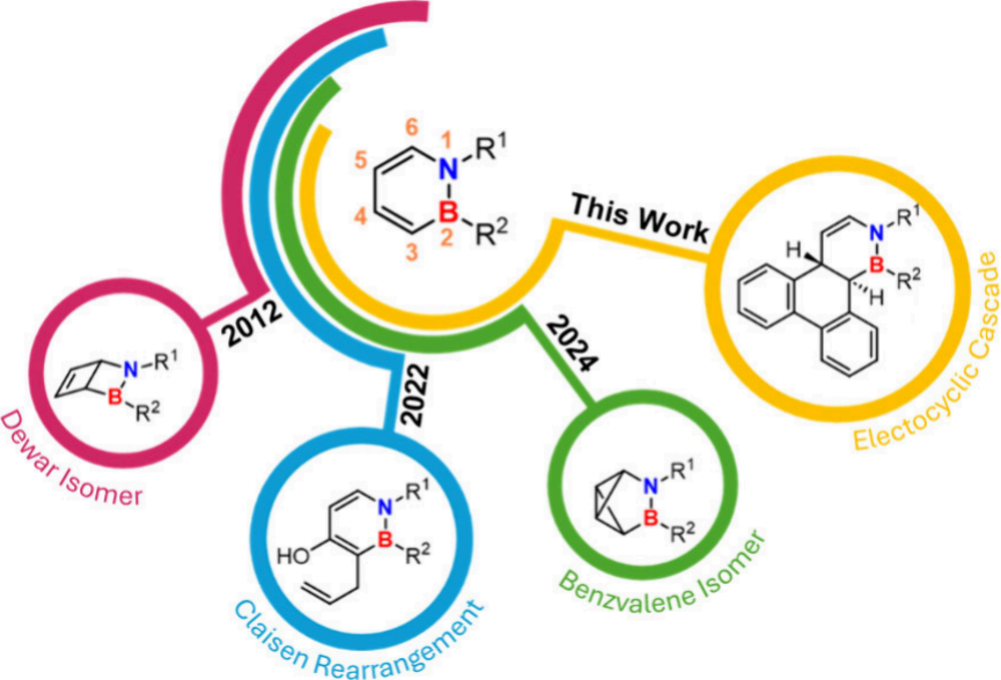
Overview of literature-known isomerization products of *^BN^* **B** with varying substituents.

**Scheme 2:**
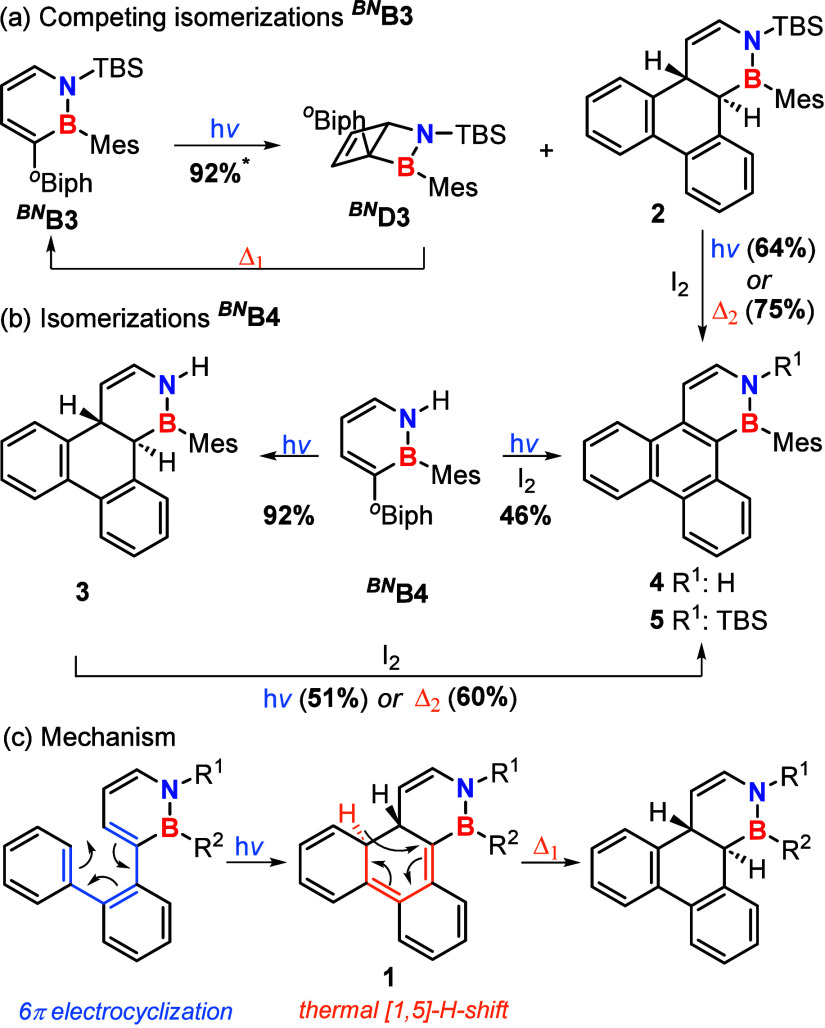
Photoisomerization of *^BN^* **B3** (a) and *^BN^* **B4** (b), with Mechanistic Proposal for the Formation of Compounds **2** and **3** (c)^a^

a hν: 280–400 nm (at 30–35 °C); Δ_1_: 100 °C; Δ_2_: 60 °C; all reactions were carried out in (deuterated) cyclohexane; oxidations were performed in the presence of 2.2 equiv of iodine; * yield after ten cycles of irradiation and heating (see SI for details); ^*o*^BiPh = *ortho*-biphenylyl.

The synthesis *^BN^* **B1** to *^BN^* **B3** follows literature-known protocols (Scheme 1).^28,29^ The cross coupling not only afforded the desired *^BN^* **B3**, but also induced a catalytic deprotection at nitrogen for traces of the reaction product (*^BN^* **B4**). Similar deprotection behavior was previously observed with DPPE or DPPP as ligands; however, no coupling occurred under those conditions.^24^ This unique reactivity thus likely originates from either 2-biphenylyl-boronic acid or the coupling product, both of which may coordinate to the palladium catalyst. Due to the large difference in polarity, the two products can be separated via normal phase column chromatography. Upon addition of a slight excess of tetrabutylammonium fluoride to *^BN^* **B3**, compound *^BN^* **B4** is obtained in very good yield (Scheme 1).^20,27^ The extension of the π-system by introduction of the *ortho*-biphenylyl substituent results in a bathochromic shift of the absorption maximum by 23 nm compared to that of C3-unsubstituted *^BN^* **B1**, yielding λ_max_ of 303 nm (Figure 2). This shift is slightly smaller than that observed for the literature-known C3-*para*-biphenylyl-substituted *^BN^* **B5** (λ_max_ = 312 nm),^24^ presumably due to reduced orbital overlap. Removal of the N-substituent (*^BN^* **B4**) causes a hypsochromic shift of the absorption maximum by 7 to 296 nm (Figure 2). This modification does not affect the general absorption pattern of the UV–vis spectra of *^BN^* **B3** and *^BN^* **B4**.

**Figure 2:**
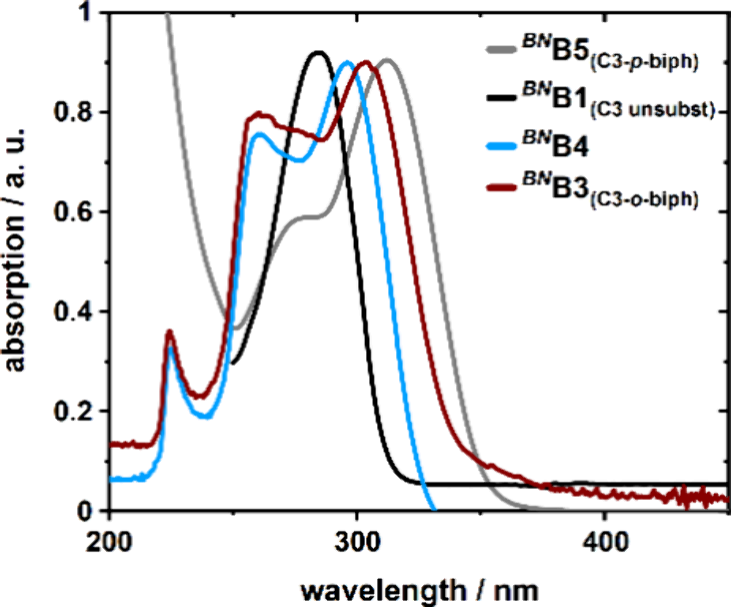
Comparative assessment of the UV–vis absorption of the compounds *^BN^* **B1** and *^BN^* **B3**-*^BN^* **B5** in cyclohexane at 25 °C. The maximum absorption was scaled to 0.9 au.

Upon irradiation of *^BN^* **B3** (solution in C_6_D_12_) with UV light (280–400 nm), formation of *^BN^* **D3** through a distrotatory 4π-electrocyclization is observed (for details, see the SI). In parallel, a second photoproduct (**2**) is formed (*^BN^* **D3**:**2** 60:40). While *^BN^* **D3** undergoes slow ring opening to *^BN^* **B3** at 70 °C, the proportion of **2** remains unchanged, indicating higher thermal stability. Consequently, repeated cycles of irradiation and heating allowed for the enrichment of **2**.

The latter was sufficiently inert to allow purification by size exclusion chromatography. Based on comprehensive NMR characterization and high-resolution mass spectrometry, **2** was subsequently identified as the product of a cascade reaction consisting of a photochemically allowed conrotatory 6π-electrocyclization followed by a thermally allowed suprafacial [1,5]-hydrogen shift (see Scheme 2).^30^ Key findings from the NMR analysis include ^1^H NMR chemical shifts of 2.62 ppm (H-3), 3.68 ppm (H-4), 6.08 ppm (H-5), and 6.51 ppm (H-6) of nonaromatic protons, whereas the remaining proton signals of the carbon backbone are located in the aromatic region of the spectrum. The large vicinal coupling constant of 18.01 Hz between H-3 and H-4 indicates the *trans* configuration of these protons. Due to the quadrupole moment of the ^11^B nucleus the adjacent C3 exhibits a characteristic, strongly broadened signal in the ^13^C NMR spectrum. Its nonquaternary nature can be confirmed by means of an ^1^H–^13^C HSQC spectrum and a ^13^C DEPT-135 spectrum. Since the sample was cooled during irradiation, ensuring temperatures below 40 °C, a very low activation barrier can be assumed for this isomerization. This is supported by DFT calculations, which arrive at an activation barrier of only 8.5 kcal/mol for this follow-up reaction (Figure S44). As the [1,5]-H shift restores the aromaticity in both C_6_ rings, it provides a significant thermodynamic driving force of 48.0 kcal/mol (Figure S44). Therefore, intermediate **1** could not be observed by ^1^H NMR spectroscopy at any point in the irradiation experiment. Theoretical^31–35^ and experimental^25,26,36–38^ studies indicate that the aromatic stabilization of *^BN^* **B** systems corresponds to approximately 70% of that of benzene. Side products arising from an alternative [1,5]-H-shift, which would restore the aromaticity of one C_6_ ring and the BN ring, are accordingly not observed. In the case of *^BN^* **B4**, this process is simplified, as *^BN^* **D4** is never detected upon irradiation and **3** is formed quantitatively within 3–5 min. Comparable behavior is also observed for other N-unsubstituted dihydroazaborinines, which do not show photoisomerization to the corresponding *^BN^* **D** or other isomers, even after prolonged irradiation at room temperature. Computational studies of 2-mesityl-1,2-dihydro-1,2-azaborinine (*^BN^* **B5**) suggest that the activation barrier for thermal ring opening of *^BN^* **B5** is only 21.6 kcal/mol, making it challenging to detect at room temperature. In agreement with this, low-temperature irradiation experiments on *^BN^* **B5** monitored by UV–vis spectroscopy show a decrease in the absorption bands associated with *^BN^* **B5**. Upon warming, partial recovery of the *^BN^* **B5** UV–vis absorption is observed, suggesting that photoisomerization of such compounds is possible, but that the resulting products are only stable at low temperatures with respect to reversion or alternative decomposition pathways (see the SI for details). By irradiation of *^BN^* **B4** in the presence of elemental iodine (Mallory conditions) an immediate oxidation to photoproduct **4** is achieved, while compound **3** is not detected (Scheme 2b). This behavior is consistent with that of carbon analogues under oxidative conditions.^39,40^ Oxidation by iodine can also be achieved starting from **2** or **3**, either photochemically (280–400 nm, at 30–35 °C) or thermally (60 °C), whereas the Mallory reaction of *^BN^* **B3** is accompanied by decomposition, presumably originating from the corresponding *Dewar* isomer (*^BN^* **D3**) formed upon irradiation (see Scheme 2a; see the SI for details).

Owning to its low solubility in apolar solvents, species **4** crystallized from *n*-hexane at −40 °C (Figure 3). The mesityl substituent at boron is orthogonal to the plane of the heterocycle, resulting in a C3–B–C7–C8 dihedral angle of 91°. Therefore, the intermolecular interactions are characterized not exclusively by π stacking between the BN-triphenylene units, but also by additional intermolecular interactions between the NH moiety and the π-systems of the mesityl and heteroaromatic fragments (Figure 3b and Figure S53). The UV–vis spectra of the photoisomers **2** and **3** differ significantly from that of the dihydroazaborinines *^BN^* **B3** and *^BN^* **B4** (Figure 4a). Their longest wavelength absorption maxima at 303 nm (*^BN^* **B3**) or 296 nm (*^BN^* **B4**) are markedly reduced in intensity and appear only as a weak shoulder in **2** and **3**. Instead, the absorption maximum at 266 nm becomes more pronounced. In this region, the UV–vis spectra of *^BN^* **B4** and *^BN^* **B5** exhibit shoulders.

**Figure 3:**
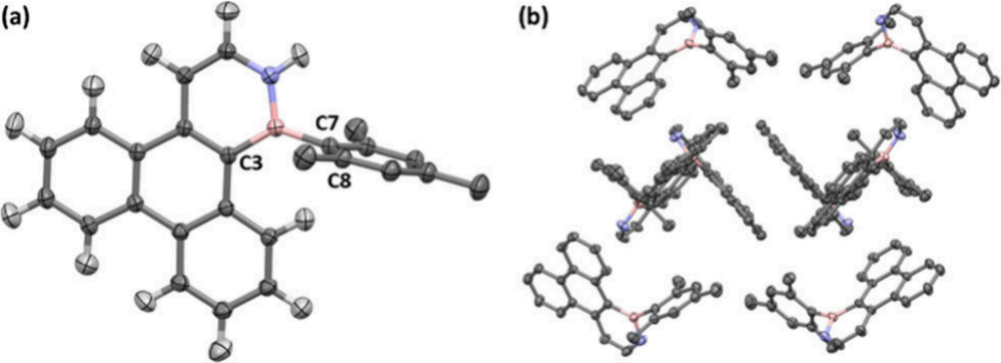
Solid-state structure (a) and packing (b) of BN-triphenylene **4**. The hydrogens are (partially) omitted for clarity. Thermal ellipsoids are shown at the 50% probability level.

**Figure 4:**
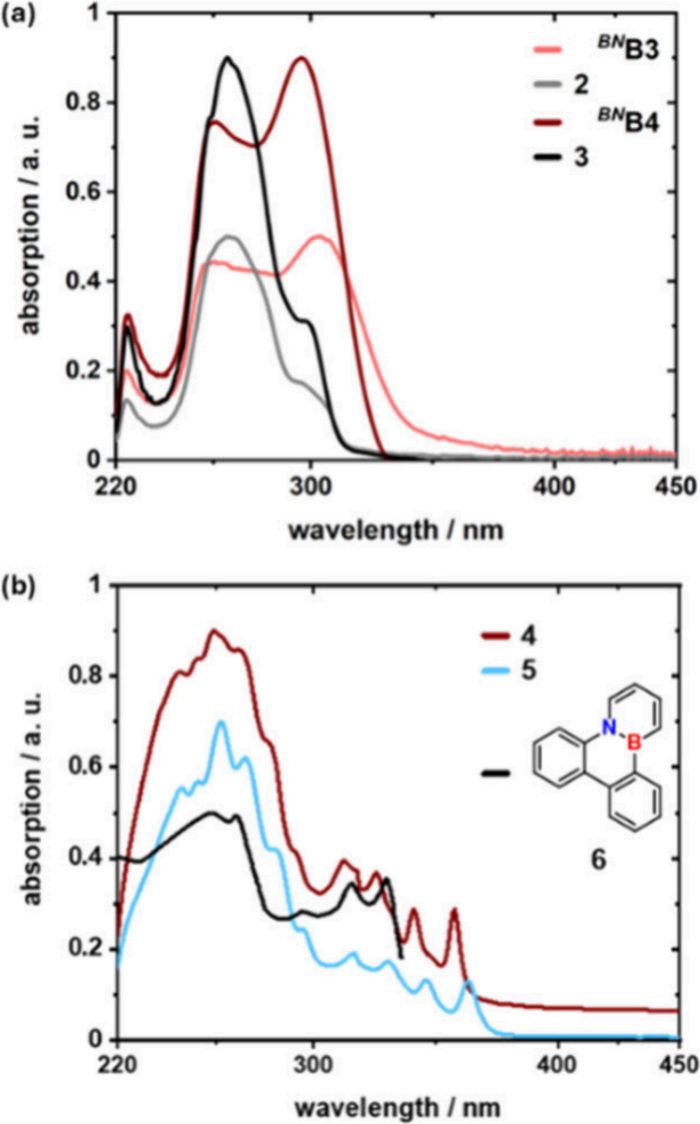
Comparison of the UV–vis absorption of *^BN^* **B3**-*^BN^* **B4** with their corresponding photoisomers **2**-**3** (cyclohexane, rt, (a)) and of **4**-**5** (cyclohexane) with **6** (ethanol, measured by Dewar et al.).^41,43^ Maximum absorptions are scaled to 0.4 or 0.9 au.

The absorption of **4** and **5** is red-shifted relative to the corresponding *^BN^* **B** due to the extended π-systems and exhibits a pronounced vibrational fine structure (Figure 4b). To date, among BN-triphenylene with a single BN unit, only the 4b,12b-BN-isomer **6** has been synthesized and characterized by Dewar et al. (Figure 4b),^41^ whereas all other regioisomers of BN-triphenylene have so far been investigated exclusively on a theoretical basis.^42^

In summary, we describe the synthesis and photo reactivity of 3-(2-biphenylyl)-1-(*tert*-butyldimethylsilyl)-2-mesityl-1,2-dihydro-1,2-azaborinine (*^BN^* **B3**). This multiphotoresponsive compound is capable of undergoing both a 4π-electrocyclization to the corresponding Dewar isomer (*^BN^* **D3**) and a cascade reaction consisting of a 6π-electrocyclization followed by a [1,5]-hydrogen shift, yielding BN-dihydrotriphenylene **2**. The latter pathway can be favored through targeted modification of the starting material. Through slight variations of the reaction conditions, a one-pot oxidation to BN-triphenylene **4** is achievable as well. These findings establish a new strategy for the selective synthesis of BN-doped polycyclic frameworks from multiphotoresponsive precursors, thereby expanding the toolbox of BN-heteroaromatic chemistry. BN-doped compounds offer access to a broad range of electronic and structural properties that are not attainable with fully carbon-based analogues. In particular, targeted positioning of the BN unit allows for fine-tuning of frontier orbital energies,^44^ molecular packing,^45^ and intermolecular interactions,^46^ thereby broadening the chemical space available for materials applications such as organic electronics, photonics, and sensing. Nevertheless, the precise incorporation of BN fragments into polycyclic aromatics and the expansion of polycyclic aromatic frameworks remain significant challenges, and improvements in synthetic methodologies are a key factor for further development in this field.^44^ Hence, the current study complements existing strategies where BN units are incorporated into the inner positions of the aromatic core (**6**)^41^ and highlights the potential advantages of the current design. In particular, the nitrogen atom on the triphenylene periphery can be utilized as anchor for installation of a wide variety of groups by electrophilic substitution.^27^

## Supplementary Material



## Data Availability

The data underlying this study are available in the published article and its Supporting Information.

## Notes

Experimental procedure, NMR/UV–vis/high resolution mass spectrometry data, crystal structure data, computational details, Cartesian coordinates (PDF)
