# Supplementary material for: Mallory-Type Reactivity of 1,2-Dihydroazaborinines: 6π-Electrocyclization-[1,5]‑H Shift Cascade toward BN-Doped Polycyclic Frameworks
Source: Org Lett. 2025 Dec 31;28(2):646–50. doi: 10.1021/acs.orglett.5c04676 (PMC12814532; doi:10.1021/acs.orglett.5c04676)
Supplement: Supplementary file 1 [file ol5c04676_si_001.pdf]

Supporting Information

Mallory-type Reactivity of 1,2-Dihydroazaborinines: 6π Electrocyclization [1,5]-H Shift Cascade towards BN-Doped Polycyclic Frameworks

Sonja M. Biebl,[a] Robert C. Richter,[a] Markus Ströbele,[b] Ivana Fleischer,[a] Holger F. Bettinger[a,*]

[a] Institut für Organische Chemie, Eberhard Karls Universität Tübingen, Auf der Morgenstelle 18, 72076 Tübingen, Germany, E-Mail: holger.bettinger@uni-tuebingen.de

[b] Institut für Anorganische Chemie, Eberhard Karls Universität Tübingen, Auf der Morgenstelle 18, 72076 Tübingen, Germany

Table of Contents

1. Methods 3
2. Analytics 3
3. Synthesis 5
3-Bromo-1-(*tert*-butyldimethylsilyl)-2-mesityl-1,2-dihydro-1,2-azaborinine (^{BN}B2) 5
3-(2-Biphenylyl)-1-(*tert*-butyldimethylsilyl)-2-mesityl-1,2-dihydro-1,2-azaborinine (^{BN}B3) 10
3-(2-Biphenylyl))-2-mesityl-1,2-dihydro-1,2-azaborinine (^{BN}B4) 15
4. Irradiation Experiments 20
Irradiation of ^{BN}B3 21
Irradiation of ^{BN}B4 28
Synthesis of compound 4 35
Reaction of 2 in the presence of iodine 41
Low-temperature UV-Vis irradiation experiment with ^{BN}B5 47
5. Crystal structures 48
6. Computations 52
Relative energies of the optimized structures (M06-2X/6-311+G(d,p)) 53
Cartesian coordinates 54
Intrinsic Reaction Coordinate 66

1. Methods

Unless otherwise noted, all experiments were carried out under inert conditions using Schlenk technique with argon or nitrogen as the protective gas or in a glove box (UNIlab Pro, MBraun). Glassware was dried before use by heating. Commercial triethylamine (water content ~ 0.2%) was refluxed over KOH for two hours and then distilled before use. The remaining chemicals used were employed as received from the manufacturer without further purification. Anhydrous solvents were obtained from Thermo Fisher Scientific Inc., Acros Organics B.V. B.A., Sigma-Aldrich or, in the case of dichloromethane, diethyl ether, n-hexane, tetrahydrofuran and toluene, from an SPS-800 solvent drying system by the manufacturer MBraun.

2. Analytics

NMR Spectroscopy: The acquisition of ^{1}H, ^{13}C-{^{1}H}, and ^{11}B-{^{1}H} NMR spectra were performed on a Bruker Advance III HD 400 MHz instrument (^{1}H spectra) at 101 MHz (^{13}C-{^{1}H} spectra), and 128 MHz (^{11}B-{^{1}H} spectra). Complementarily, spectra of these nuclei were recorded on a Bruker Advance III HD 300 MHz NanoBay at 300 MHz (^{1}H spectra), 76 MHz (^{13}C-{^{1}H} spectra), and 96 MHz (^{11}B-{^{1}H} spectra). For further NMR measurements (kinetic experiments), a Bruker Avance III HDX 600 spectrometer was employed with a measuring frequency of 600 MHz (^{1}H spectra), 151 MHz (^{13}C-{^{1}H} spectra) or 192 MHz (^{11}B-{^{1}H} spectra) and on a Bruker Avance III HDX 700 with a measuring frequency of 700 MHz (^{1}H spectra) or 176 MHz (^{13}C-{^{1}H} spectra). All obtained NMR spectra were referenced to the solvent peak. Deuterated dichloromethane (5.32 ppm), or cyclohexane (1.38 ppm) from Sigma-Aldrich or Deutero were used for referencing. Structural assignments were made with additional information from gCOSY, gHSQC, and gHMBC experiments.

MPLC Chromatography: Column chromatographic purifications were performed using the puriFlash 430 in combination with pre-packed silica gel columns (particle size: 30 µm) from Interchim. Detection was carried out using a puriFlash One Series UV detector (DAD 200-600 nm) from the same manufacturer.

All solvents used were of HPLC-grade purity. Solvent mixtures are indicated as V/V ratio.

GPC Chromatography: The size exclusion chromatography was conducted on a Recycling Preparative HPLC and GPC (LaboACE LC-7080 Plus II) device and a JAIGEL-2.5 HR Plus column both from Japan Analytical Industry Co.

UV/VIS Spectroscopy: For determining the absorption maxima of the presented 1,2-substituted 1,2-dihydro-1,2-azaborinines, a Lambda 1050 UV/VIS/NIR spectrometer (Perkin Elmer) with a 3D WB detection module was used, operated with the accompanying UV Win Lab software (Version 2.0.2). The wavelength range covered was from 250 nm to 850 nm. All solvents used had a cut-off wavelength below 250 nm and were water-free.

Low-temperature UV-Vis measurements: For low-temperature measurements (273.15 K), an Optistat DN2 cryostat connected to a Mercury iTC control unit (Oxford Instruments) was used.

Irradiation experiments: As light source an Osram HBO-500-W/2 high pressure mercury lamp in an Oriel housing with quartz optics and a dichroic mirror (280 - 400 nm) was applied. No additional filters were used.

The samples for the kinetic studies prepared in quartz glas J.-Young-NMR tubes as the reaction vessel.

Mass spectrometry: High resolution mass spectra were recorded on a HR-ESI/APCI-TOF device (maXis 4G, Bruker) or a HR-ESI-Orbitrap-MS (Q exactive HF, Thermo Scientific) for ESI ionization. The sample was dissolved in dichloromethane and injection was carried out using a syringe pump.

X-Ray Crystallography: Crystaly suitable for X-Ray diffraction were grown by vapour diffusion with *n*-hexane and dichloromethane at room temperature. Single crystals were selected, coated with Parabar 10312 and fixed on a microloop.

Data were collected on a XtaLAB Synergy, Dualflex, HyPix diffractometer using ω scans with Cu K_α radiation. The crystal was kept at a steady temperature during data collection. The diffraction pattern was indexed and the total number of runs and images was based on the strategy calculation from the program CrysAlisPro 1.171.42.49, which was also used for refining the unit cell. The structure was solved with the **ShelXT** 2018/2 solution program[1-3] using dual methods and by using **Olex2** 1.5-ac5-024 as the graphical interface.[4] The model was refined with **olex2.refine** 1.5-ac5-024 using full matrix least squares minimization on F^2.[5]

3. Synthesis

1-(*tert*-Butyldimethylsilyl)-2-mesityl-1,2-dihydro-1,2-azaborinine (^{BN}**B1**) was synthesized according to Richter *et al.*[6]

2-Mesityl-1,2-dihydro-1,2-azaborinin (^{BN}**B5**) was synthesized according to Lee *et al.*[7]

3-Bromo-1-(*tert*-butyldimethylsilyl)-2-mesityl-1,2-dihydro-1,2-azaborinine (^{BN}B2)

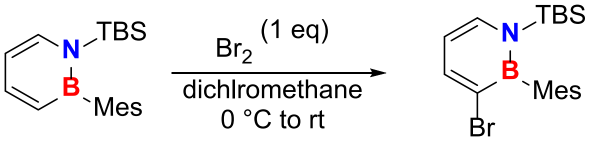

The synthesis was carried out according to a procedure by Bettinger *et al.*[8] 1-(*tert*-butyldimethylsilyl)-2-mesityl-1,2-dihydro-1,2-azaborinine (**1**) (1 g, 3.2 mmol, 1 eq) was solved in dichloromethane (10 mL) and cooled to 0 °C. A solution of bromine (0.09 mL, 3.5 mmol, 1.1 eq) in dichloromethane (10 mL) was added dropwise over 1 h. The reaction mixture was stirred for 30 minutes at 0 °C. The solution was allowed to reach room temperature and stirred for another 30 minutes, before a saturated solution of Na_2S0_3 (20 mL) was added. The aqueous layer was extracted three times with *n*-hexane (15 mL) and the combined organic layers were dried over $MgSO_4$. After removing of the solvent, the crude product was purified by column chromatography (silica, *n*-hexane/dichloromethane gradient). The product was obtained as colorless solid/crystals (0.8774 g, 70%).

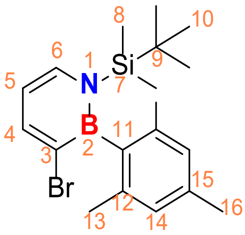

$C_{19}H_{29}BBrNSi$ (390.25 g/mol)

^{1}H-NMR (400 MHz, **CD_2Cl_2**): δ = 7.89 (dd, $^{3}J_{HH}$ = 7.18 Hz, $^{4}J_{HH}$ = 0.86 Hz, 1H, H-4), 7.48 (dd, $^{3}J_{HH}$ = 6.78 Hz, $^{4}J_{HH}$ = 0.86 Hz, 1H, H-6), 6.80 (s, 2H, H-14), 6.31 (ps. t, J = 6.89 Hz, 1H, H-5), 2.29 (s, 3H, H-16), 2.06 (s, 6H, H-13), 0.92 (s, 9 H, H-10), -0.01 (s, 6H, H-8) ppm.

^{13}C-{^{1}H}-NMR (100 MHz, **CD_2Cl_2**): δ = 144.8 (C4), 139.4 (C12/C15), 138.7 (C6), 137.4(), 127.2 (C14), 111.8 (C5), 27.6 (C10), 22.9 (C13), 21.4 (C16), 19.5 (C9), -3.0 (C8) ppm.

^{11}B-{^{1}H}-NMR (128 MHz, **CD_2Cl_2**): δ = 39.5 ppm.

The data are consistent with the literature.[8]

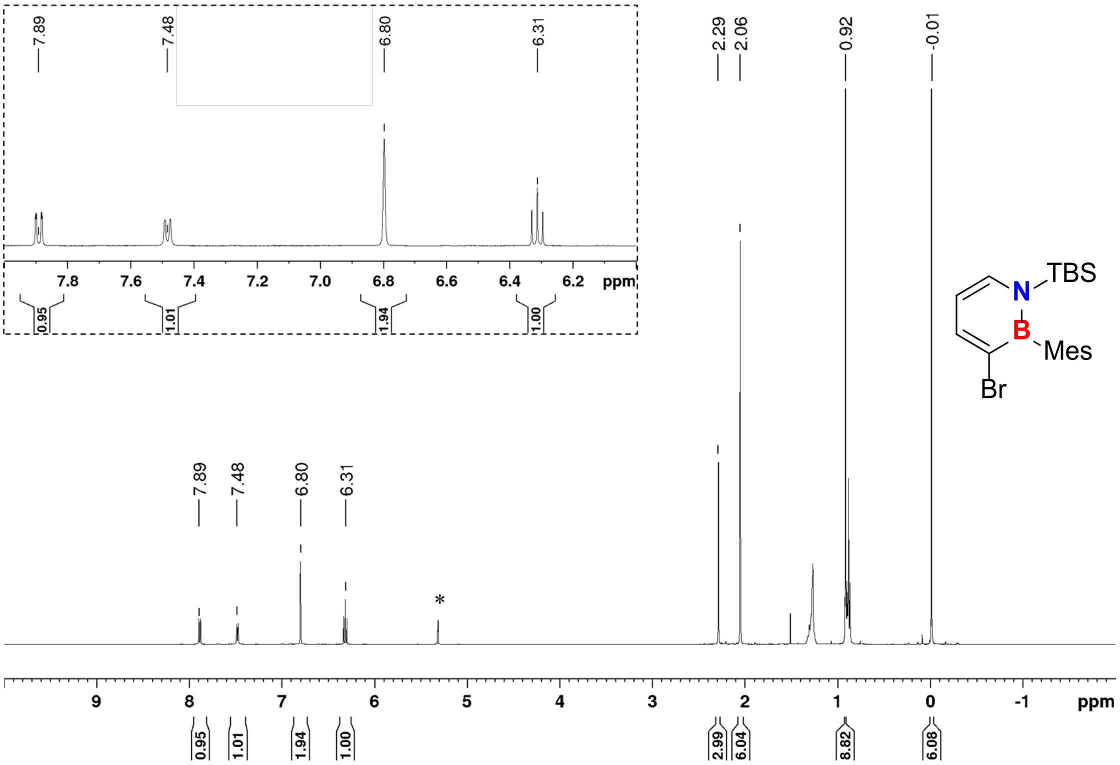

Figure S1. ^{1}H-NMR spectrum of compound ^{BN}**B2** in CD_2Cl_2 measured at a 400 MHz spectrometer. The enlarged section shows the region between 6 and 8 ppm for a better visibility of the aromatic signals. The solvent signal is marked with an asterisk.

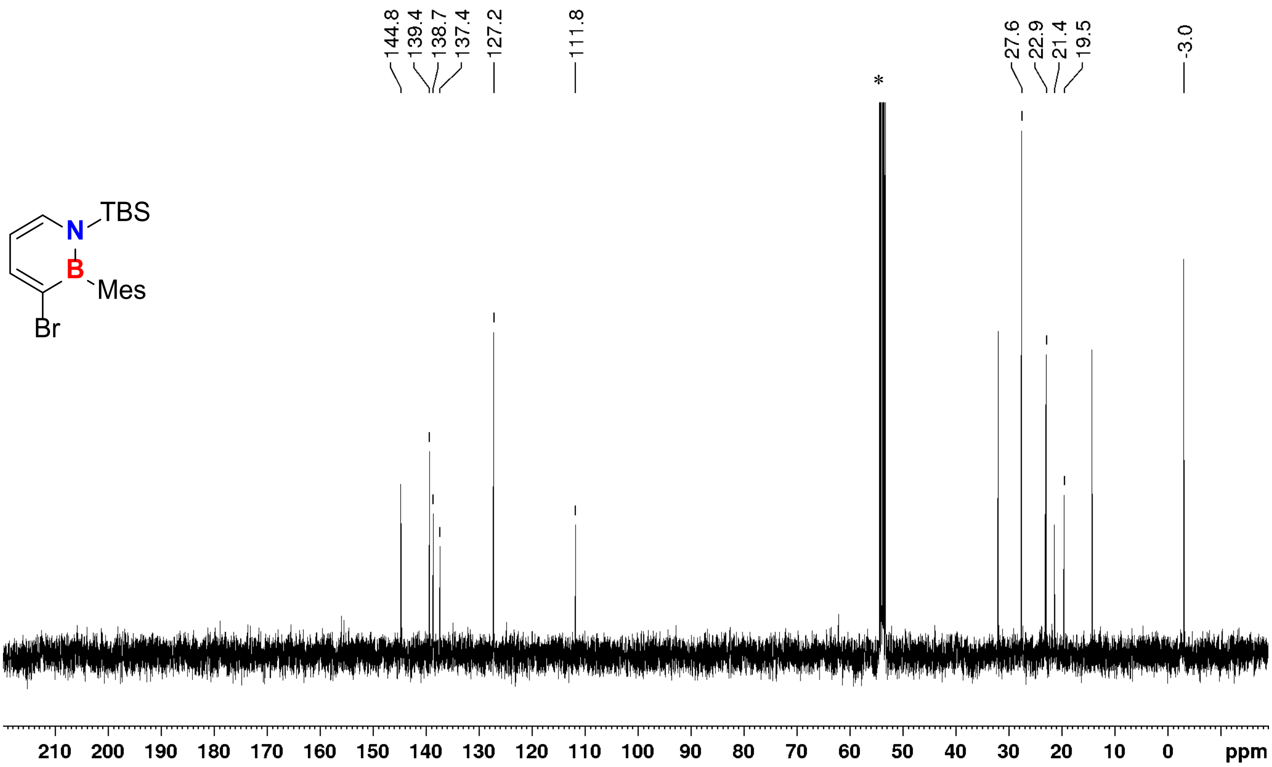

Figure S2. ^{13}C-{^{1}H}-NMR spectrum of compound ^{BN}**B2** in CD_2Cl_2 measured at a 400 MHz spectrometer. The solvent signal is marked with an asterisk.

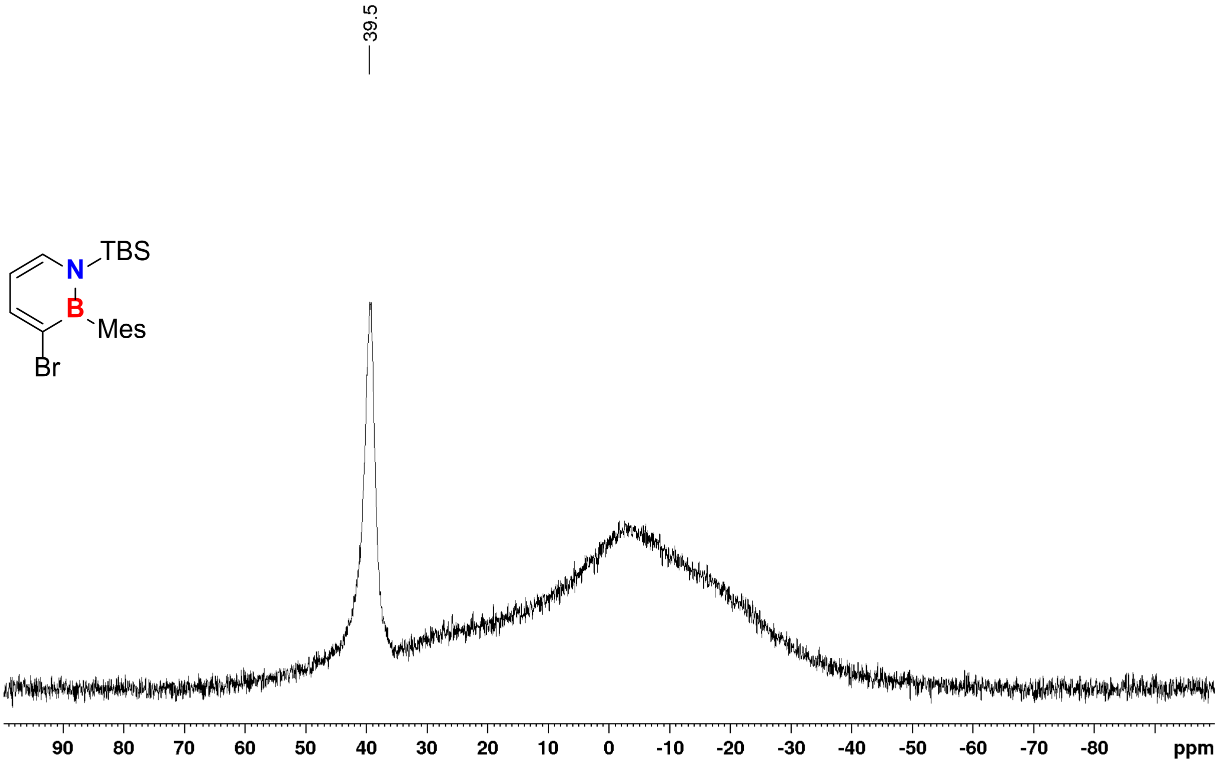

Figure S3. ^{11}B-{^{1}H}-NMR spectrum of compound ^{BN}**B2** in CD_2Cl_2 measured at a 400 MHz spectrometer. The broad signal between -30 and 30 ppm) is due to the borosilicate glass of the NMR tube.

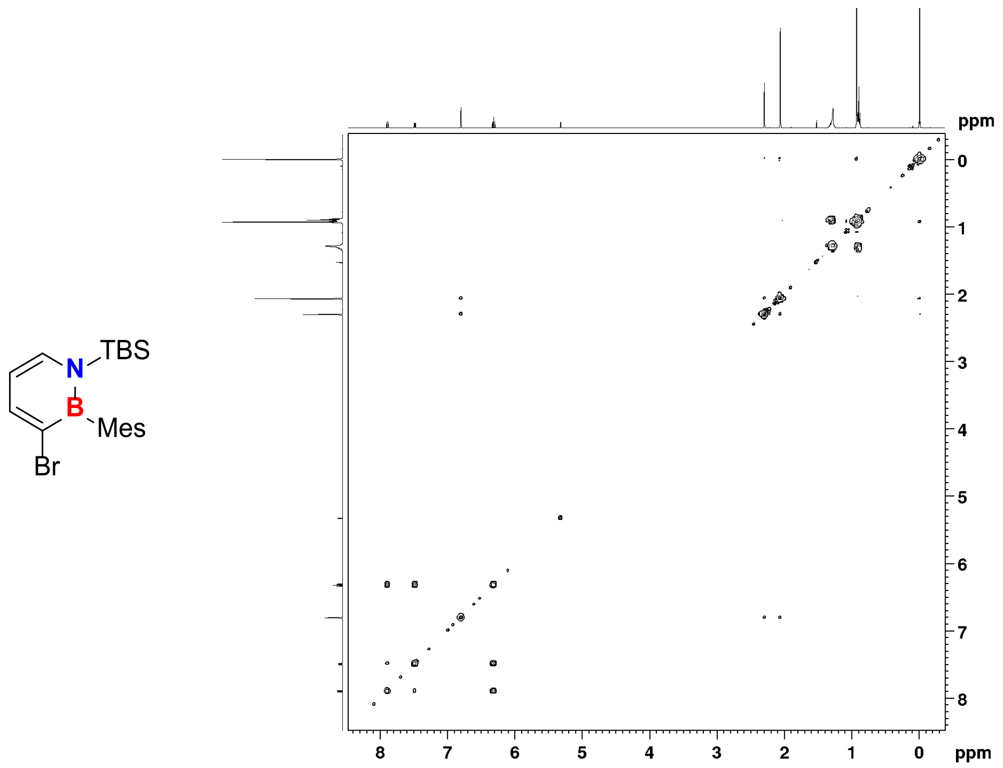

Figure S4. ¹H-¹H-COSY-NMR spectrum of compound ^{BN}**B2** in CD_2Cl_2 measured at a 400 MHz spectrometer.

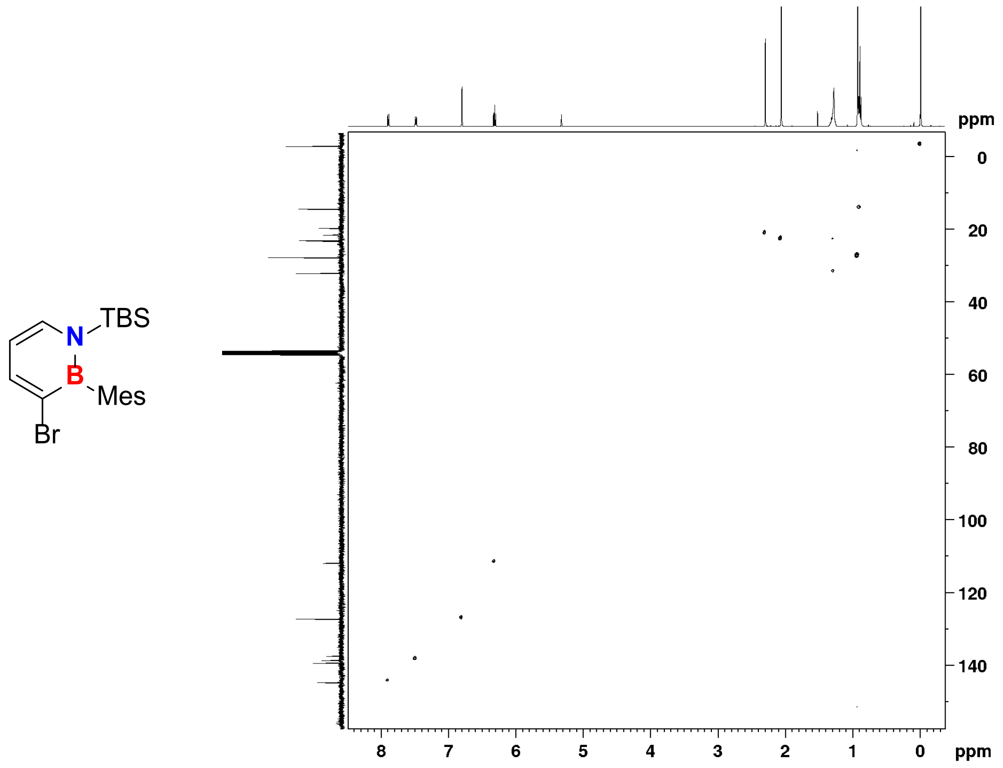

Figure S5. ¹H-¹³C-HSQC-NMR spectrum of compound ^{BN}**B2** in CD_2Cl_2 measured at a 400 MHz spectrometer.

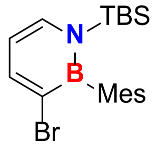

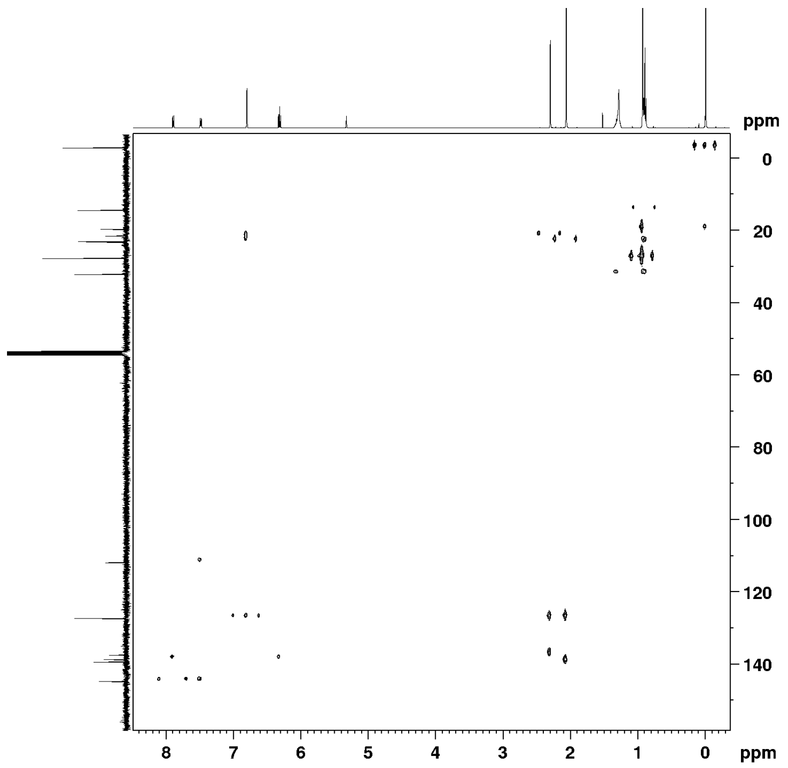

Figure S6. ^{1}H-^{13}C-HMBC-NMR spectrum of compound ^{BN}**B2** in CD_2Cl_2 measured at a 400 MHz spectrometer.

3-(2-Biphenylyl)-1-(*tert*-butyldimethylsilyl)-2-mesityl-1,2-dihydro-1,2-azaborinine (^{BN}B3)

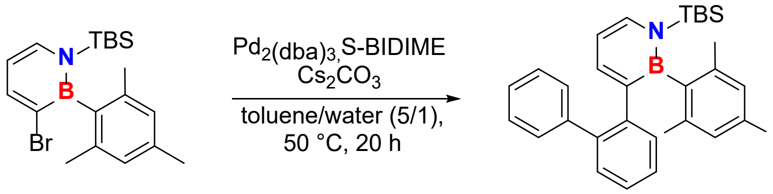

The synthesis was performed following procedures reported by Song *et al.*[9] and Bettinger *et al.*[8]

3-Bromo-1-(*tert*-butyldimethylsilyl)-2-mesityl-1,2-dihydro-1,2-azaborinine (^{BN}**B2**) (39.0 mg, 0.1 mmol, 1.0 equiv), 2-biphenylboronic acid (25.7 mg, 0.13 mmol, 1.3 equiv), dry Cs_2CO_3 (48.9 mg, 0.15 mmol, 1.5 equiv), $Pd_2(dba)_3$ (2 mg, 0.002 mmol, 2 mol%) and S-BIDIME (2 mg, 0.006 mmol, 6 mol%) were dissolved in dry, degassed toluene (1.5 mL) and degassed water (0.3 mL). The reaction mixture was stirred for 16 h at 50 °C. Subsequently, 2 mL of distilled water were added, and the aqueous phase was extracted with *n*-hexane (3 × 2 mL). The combined organic layers were dried over $MgSO_4$, and the solvent was removed under reduced pressure. The crude product was purified by column chromatography on silica gel using a n-hexane/dichloromethane gradient to afford the product as a colorless solid (25.1 mg, 0.054 mmol, 54%).

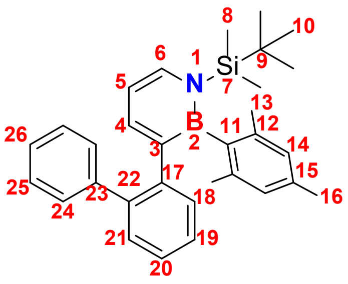

$C_{31}H_{38}BNSi$ (463.55 g/mol)

^{1}**H-NMR** (700 MHz, **C_6D_{12}**): δ = 7.29 (dd, $^{3}J_{HH}$ = 6.83 Hz, $^{4}J_{HH}$ = 1.13 Hz, 1H, H-4), 7.22 (dd, $^{3}J_{HH}$ = 6.68 Hz, $^{4}J_{HH}$ = 1.19 Hz, 1H, H-6), 6.99 (m, 5H, H-124/H-25/H-26), 6.90 (m, 3H, H-18/H-19/H-20), 6.79 (dm, $^{3}J_{HH}$ = 7.60 Hz, 1H, H-21), 6.45 (s, 2H, H-14), 6.22 (ps. t, 1H, H-5), 2.17 (s, 3H, H-16), 1.62 (br. s, 6H, H-13), 0.84 (s, 9H, H-10), -0.07 (s, 6H, H-8) ppm.

^{13}**C-{^{1}H}-NMR** (151 MHz, **C_6D_{12}**): δ = 145.3, 144.5, 144.1, 143.8, 141.0, 140.3, 140.2, 137.1, 136.8, 131.6, 131.0, 130.8, 127.7, 127.5, 126.4, 126.1, 126.0, 111.5, 27.8, 26.8, 23.3, 21.4, 19.8, -3.2 ppm.

^{11}**B-{^{1}H}-NMR** (128 MHz, **C_6D_{12}**): δ = 40.2 ppm.

HRMS (APCI) m/z: $[M + H]^+$ Calcd for $C_{31}H_{39}BNSi$ 464.2945; Found 464.2940.

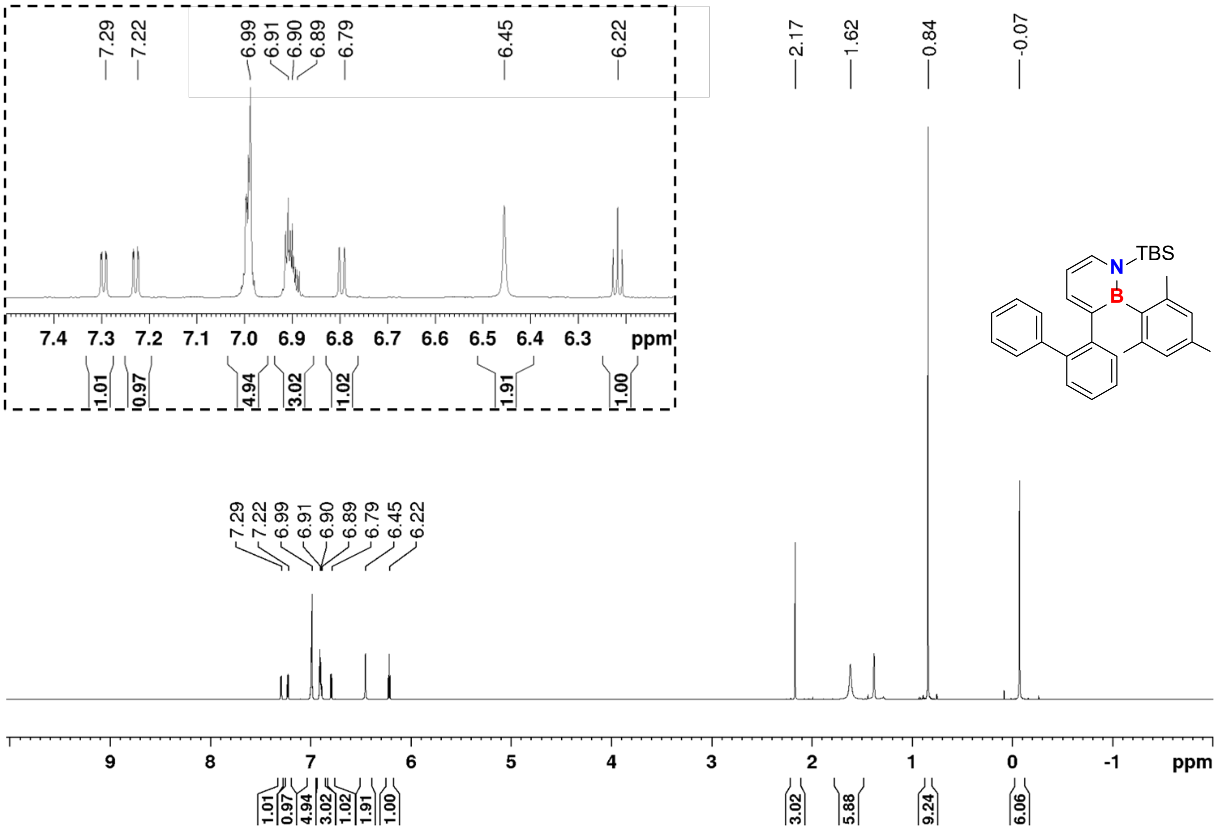

Figure S7. ^{1}H NMR spectrum of compound **BNB3** in C_6D_{12} measured on a 600 MHz spectrometer.

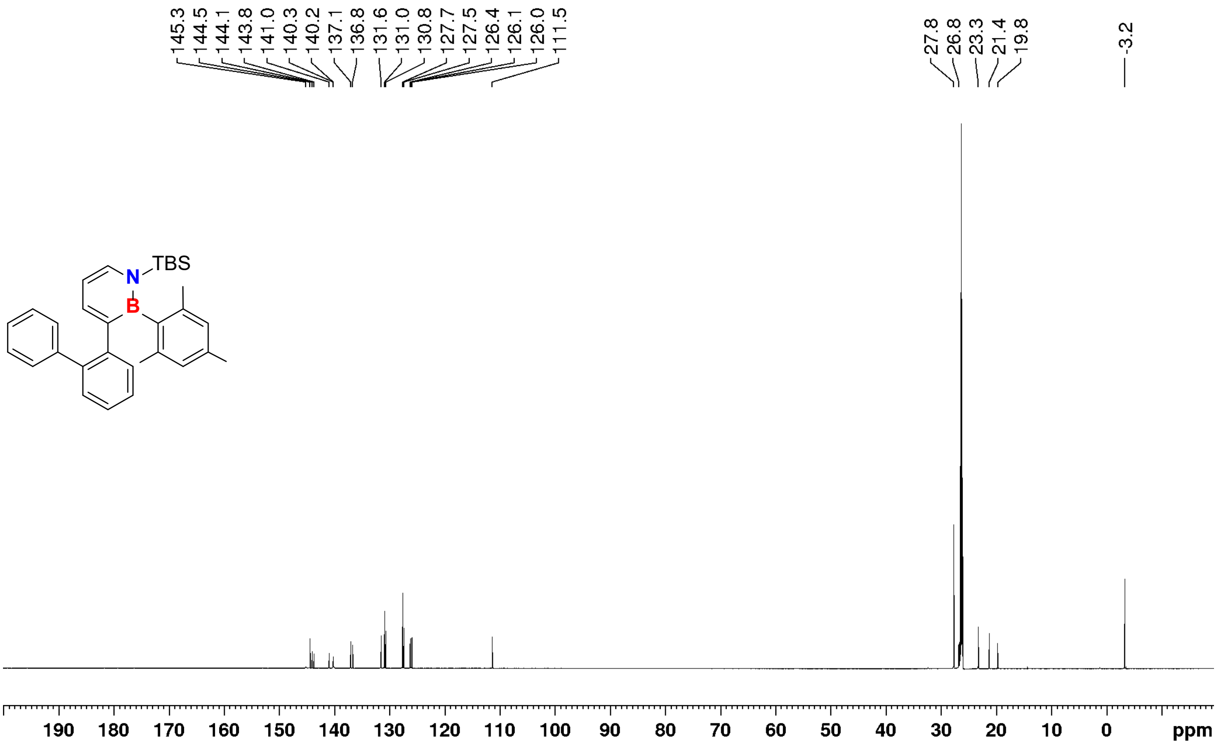

Figure S8. ^{13}C-{^{1}H} NMR spectrum of compound ^{BN}**B3** in C_6D_{12} measured on a 600 MHz spectrometer.

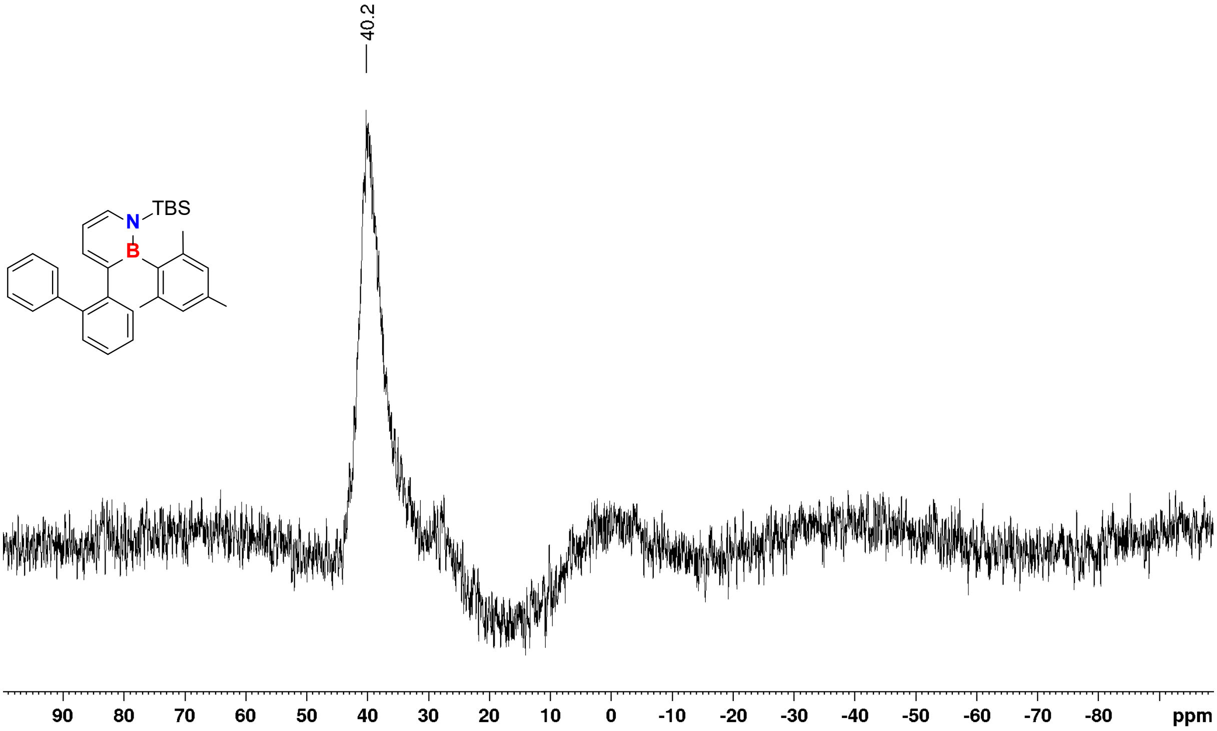

Figure S9. ^{11}B-{^{1}H} NMR spectrum of compound *BN***B3** in C_6D_{12} measured on a 600 MHz spectrometer.

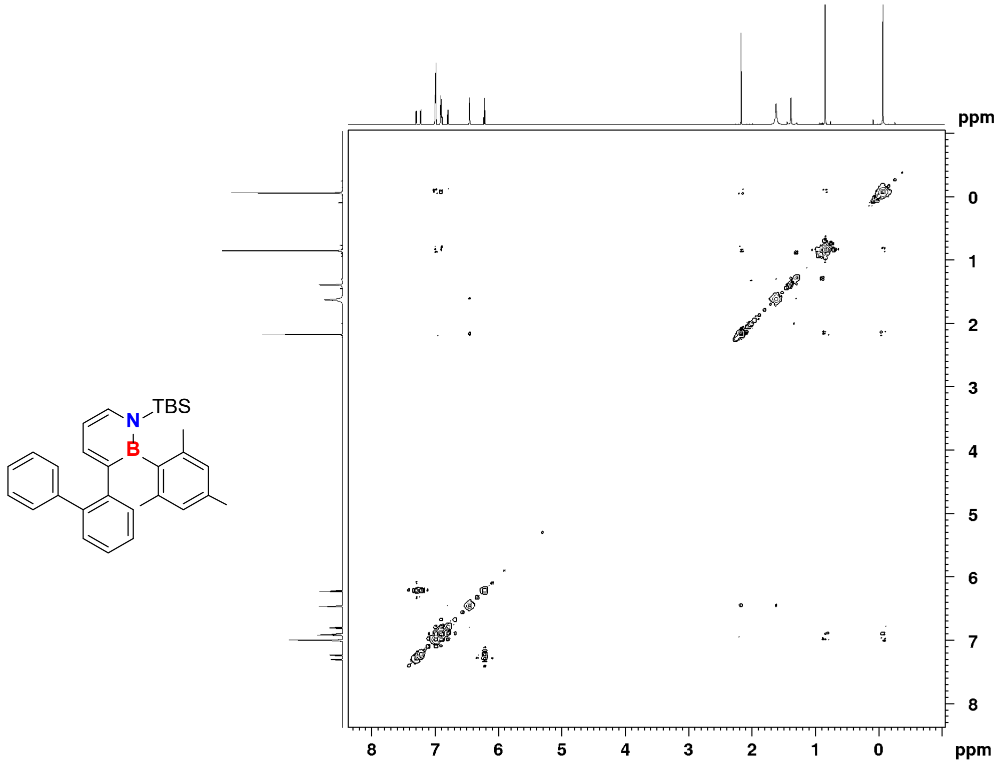

Figure S10. ^{1}H-^{1}H-COSY NMR spectrum of compound *BN***B3** in C_6D_{12} measured on a 600 MHz spectrometer.

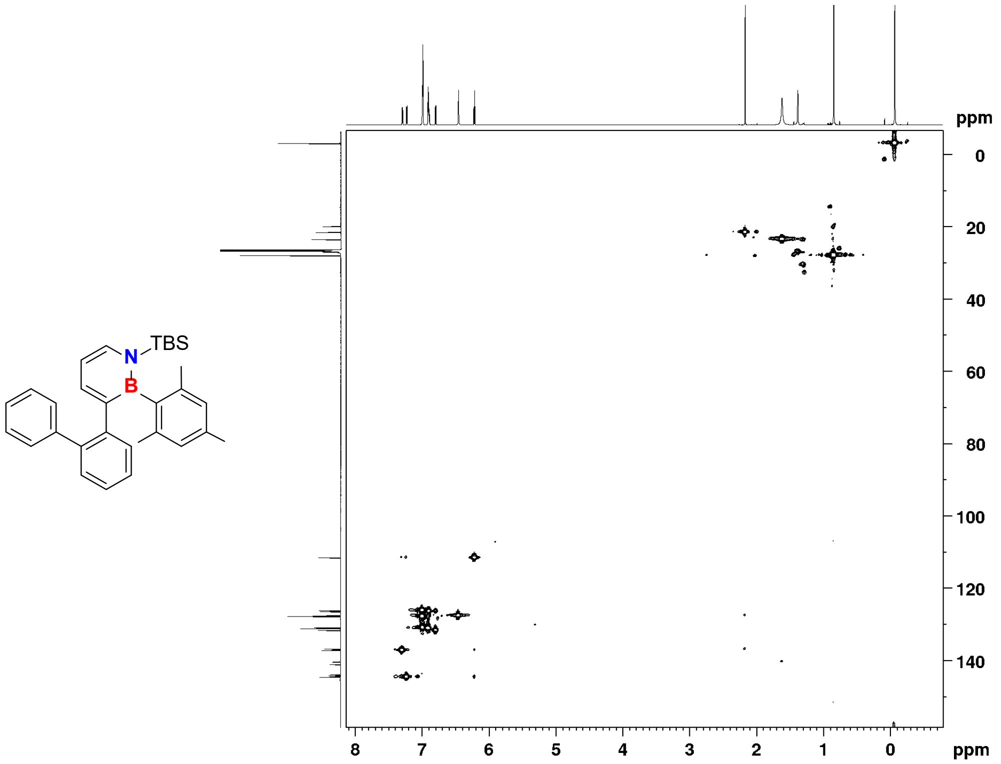

Figure S11. ^{1}H-^{13}C-HSQC NMR spectrum of compound ^{BN}**B3** in C_6D_{12} measured on a 600 MHz spectrometer.

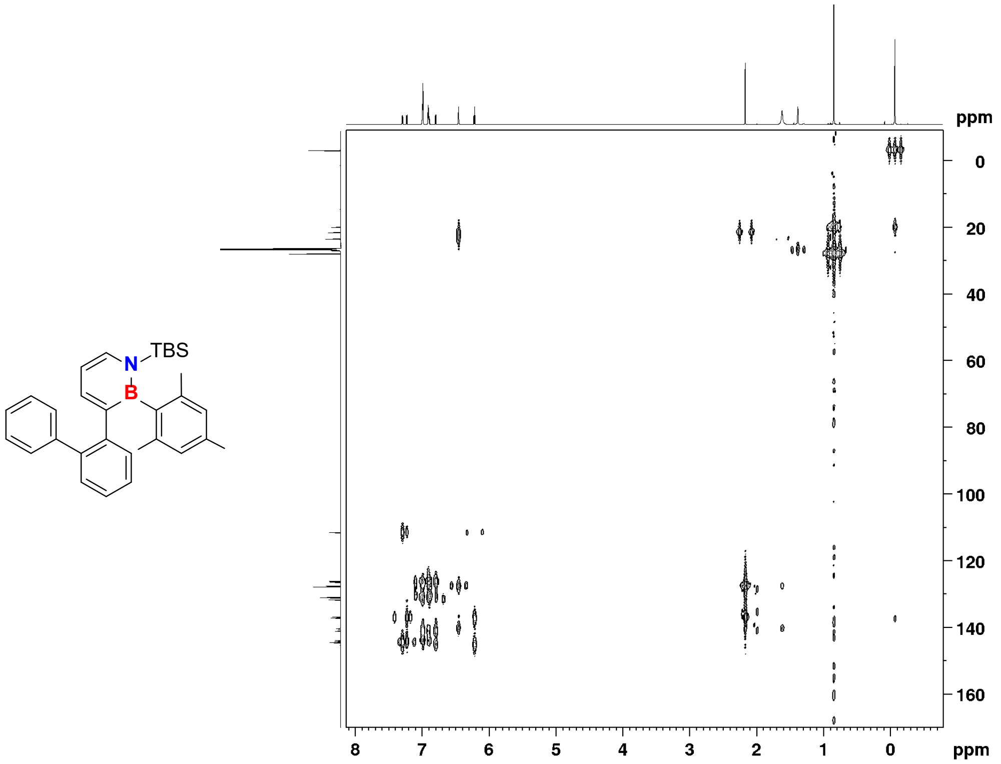

Figure S12. ^{1}H-^{13}C-HMBC NMR spectrum of compound ^{BN}**B3** in C_6D_{12} measured on a 600 MHz spectrometer.

3-(2-Biphenylyl))-2-mesityl-1,2-dihydro-1,2-azaborinine (*BN*B4)

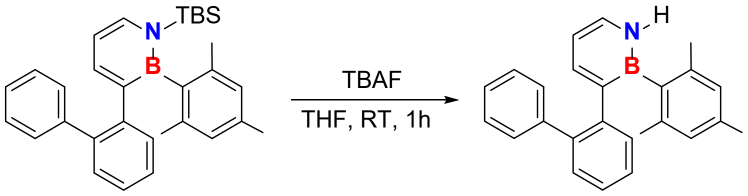

The synthesis was performed similar to a procedure reported by Liu *et al.*[7]

Dihydroazaborinine ***BN*B3** (10 mg, 0.02 mmol, 1.0 equiv) was dissolved in dry tetrahydrofuran (2 mL) and a solution of tetrabutylammonium fluoride (1 M in THF, 0.03 mL, 1.2 equiv) was added dropwise. The reaction mixture was stirred at room temperature for 1 h and then *quenched* with deionized water (5 mL). The aqueous phase was extracted three times with *n*-hexane (3 × 5 mL). The combined organic layers were dried over magnesium sulfate, filtered, and the solvent was removed under reduced pressure. Purification by column chromatography on silica gel using a *n*-hexane/dichloromethane gradient afforded the product as a colorless solid (7.1 mg, 0.02 mmol, 93%).

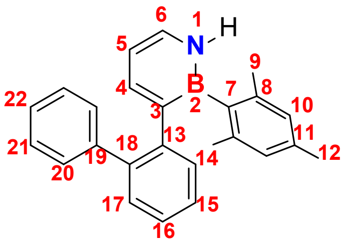

$C_{25}H_{24}BN$ (349.28 g/mol)

^{1}H-NMR (600 MHz, **C_6D_{12}**): δ = 7.88 (br. s, 1H, H-1), 7.43 (d, $^3J_{HH}$ = 6.91 Hz, 1H, H-4), 7.25 (ps. t, 1H, H-6), 7.07 (m, 7H, H-14/H-15/H-16/H-17/H-21/H-22), 6.90 (m, 2H, H-20), 6.53 (s, 2H, H-10), 6.27 (ps. t, 1H, H-5), 2.13 (s, 3H, H-12), 1.61 (s, 6H, H-9) ppm.

^{13}**C-{^{1}H}-NMR** (151 MHz, **C_6D_{12}**): δ = 143.6, 143.4, 143.0, 140.3, 139.8, 135.9, 131.2, 130.7, 130.2, 129.8, 126.9, 126.7, 126.2, 125.4, 125.3, 109.9, 21.8, 20.4 ppm.

^{11}**B-{^{1}H}-NMR** (193 MHz, **C_6D_{12}**): δ = 36.9 ppm.

HRMS (APCI) m/z: $[M + H]^+$ Calcd for $C_{25}H_{25}BN$ 349.2001; Found 349.1999.

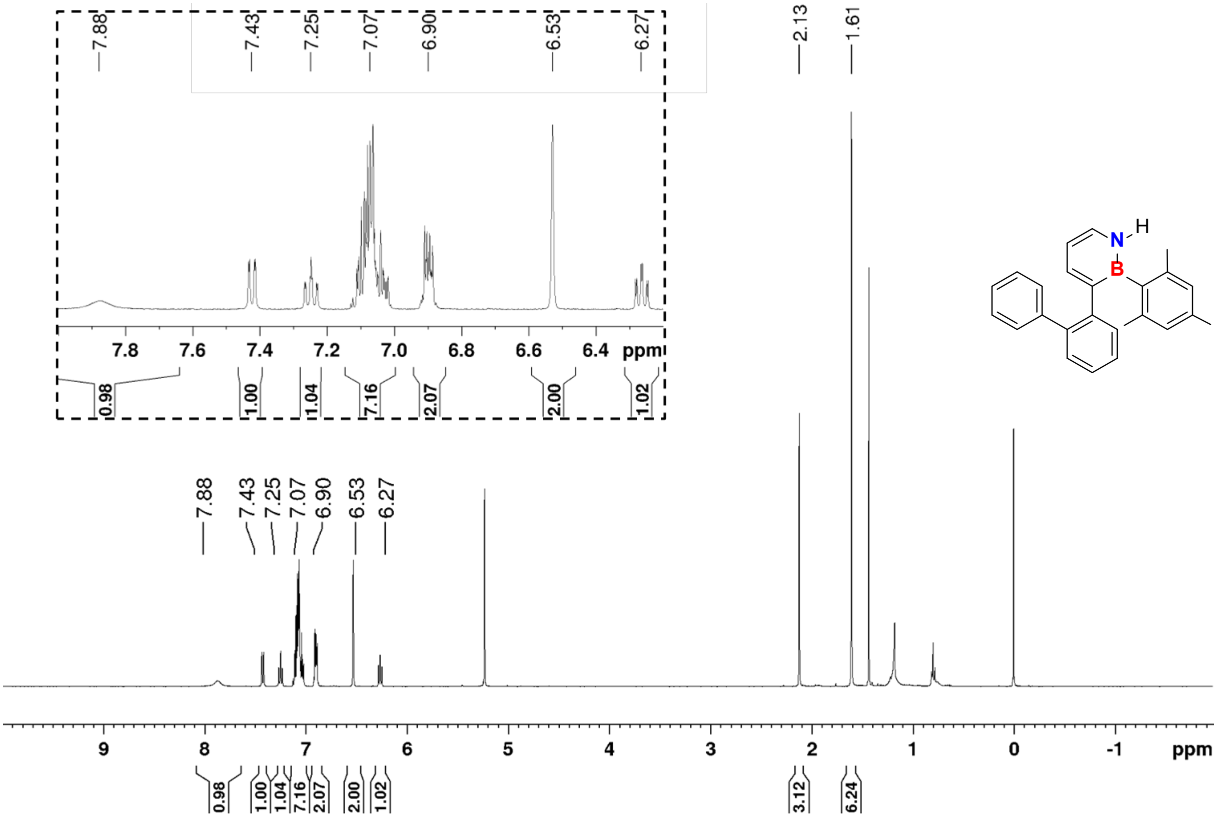

Figure S13. ^{1}H NMR spectrum of compound ^{BN}**B4** in C_6D_{12} measured on a 600 MHz spectrometer.

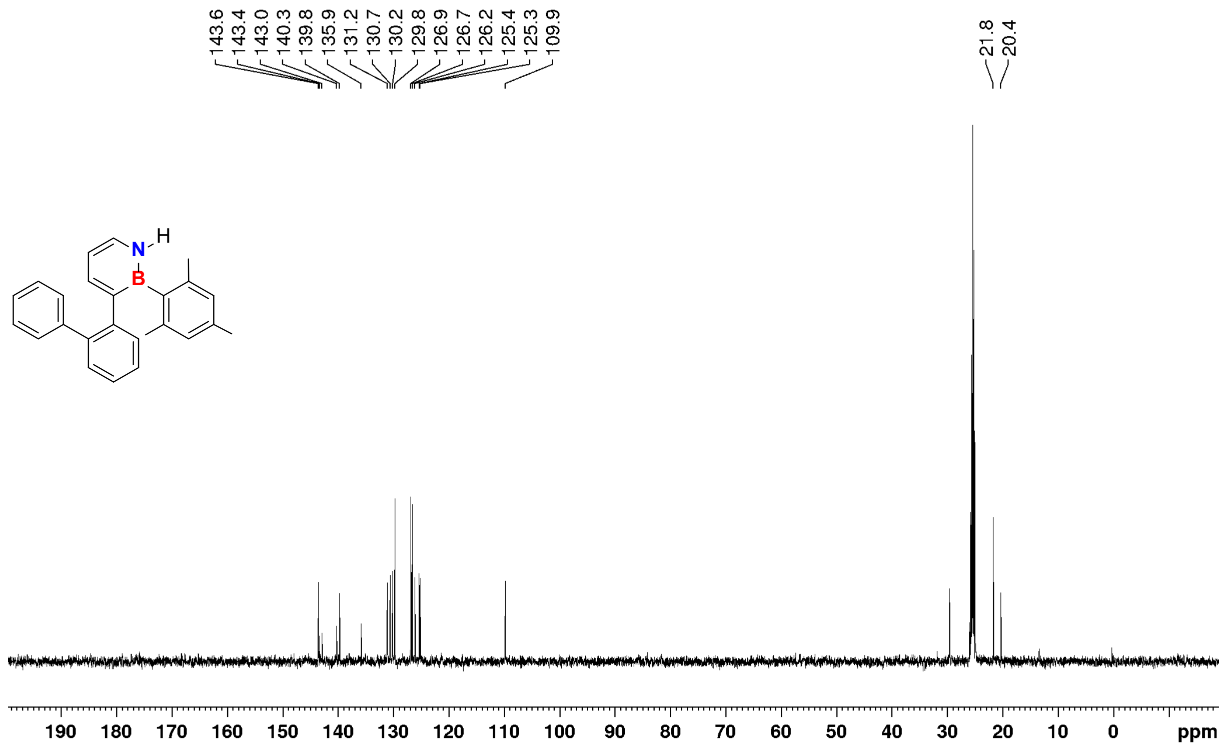

Figure S14. ^{13}C-{^{1}H} NMR spectrum of compound ^{BN}**B4** in C_6D_{12} measured on a 600 MHz spectrometer.

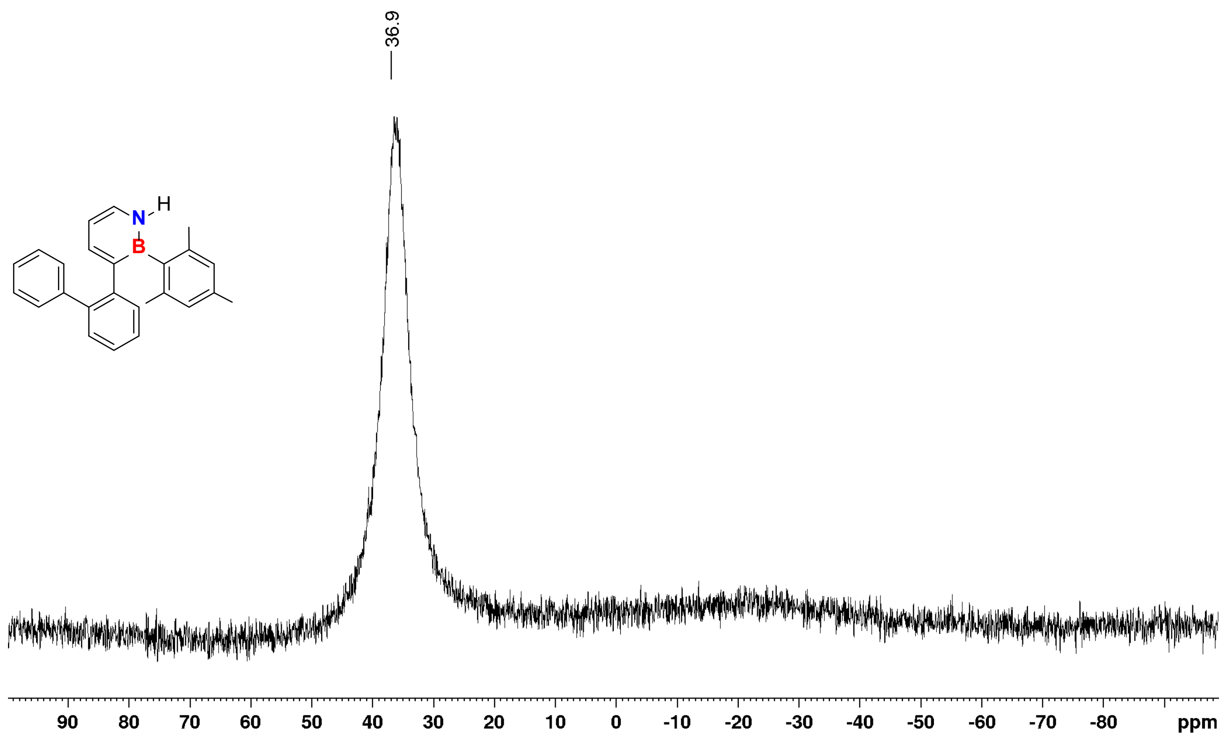

Figure S15. ^{11}B-{^{1}H} NMR spectrum of compound ^{BN}**B4** in C_6D_{12} measured on a 600 MHz spectrometer.

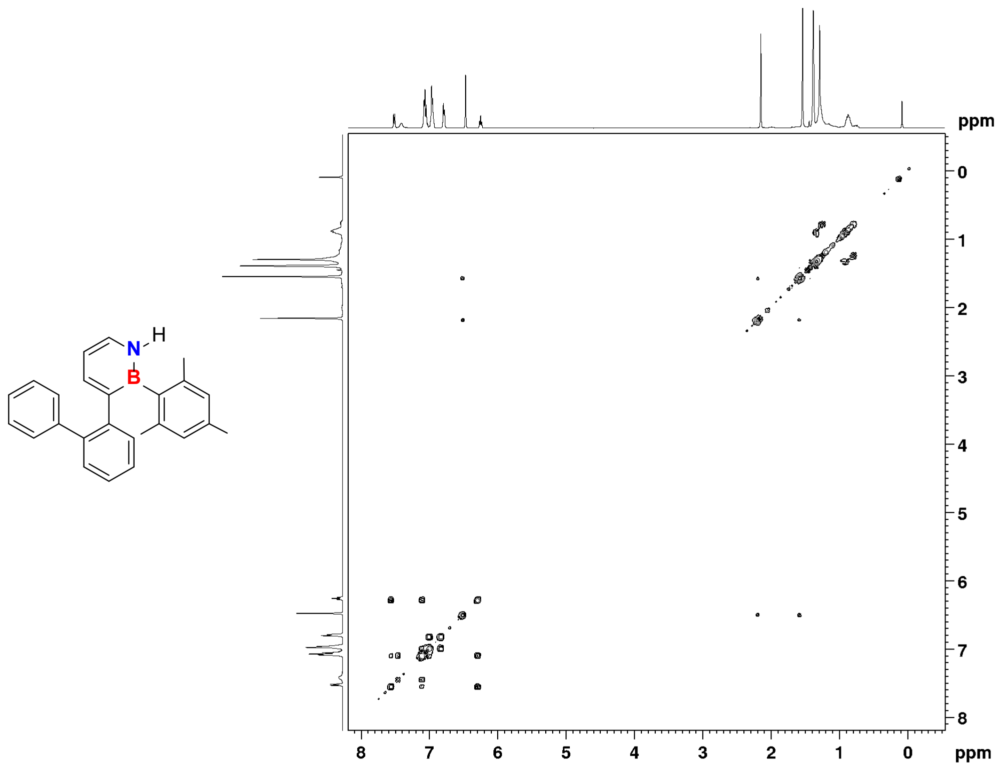

Figure S16. ^{1}H-^{1}H-COSY NMR spectrum of compound ^{BN}**B4** in C_6D_{12} measured on a 600 MHz spectrometer.

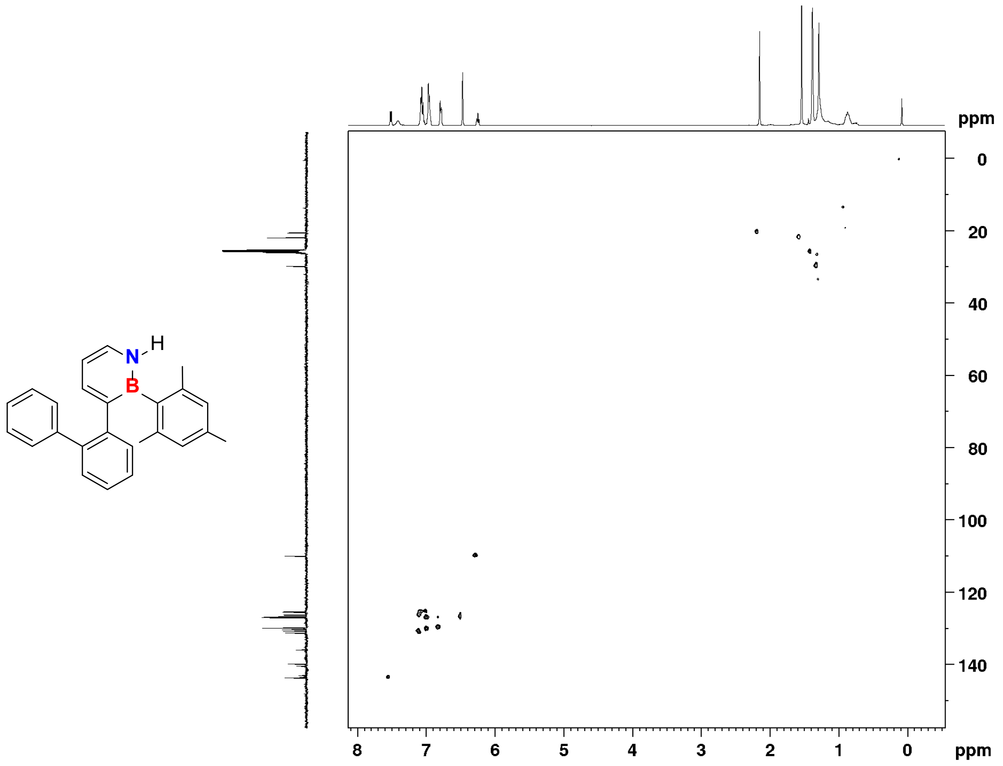

Figure S17. ^{1}H-^{13}C-HSQC NMR spectrum of compound ^{BN}**B4** in C_6D_{12} measured on a 600 MHz spectrometer.

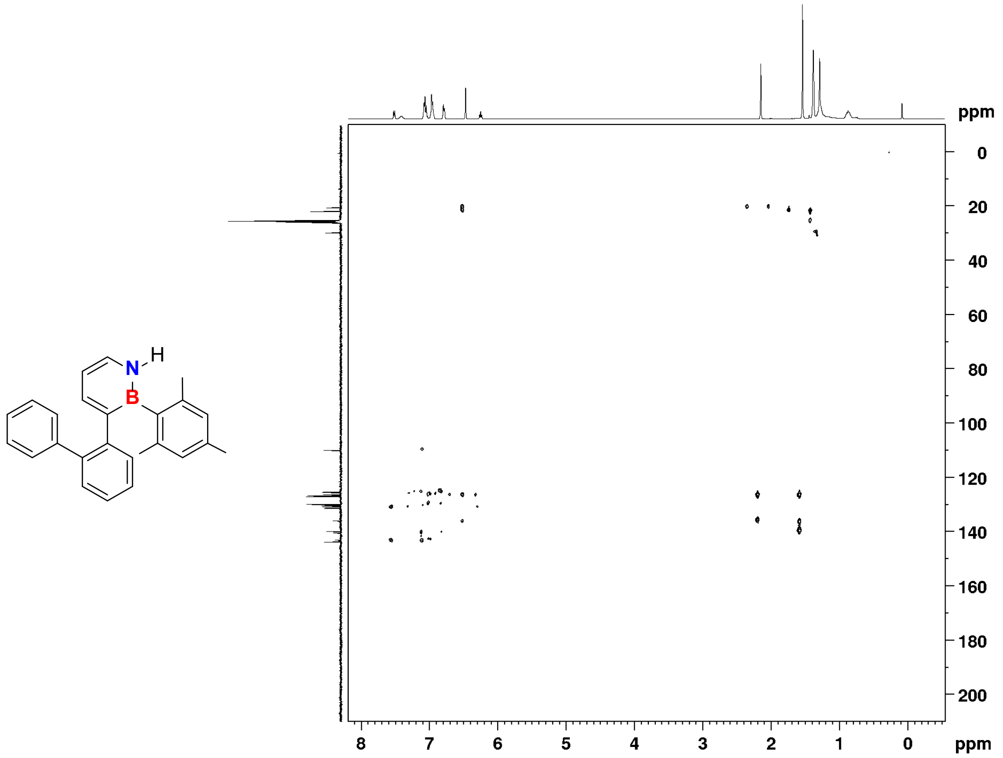

Figure S18. ^{1}H-^{13}C-HMBC NMR spectrum of compound ^{BN}**B4** in C_6D_{12} measured on a 600 MHz spectrometer.

4. Irradiation Experiments

All irradiations were carried out using mercury high pressure lamp and a dichroitic mirror that selects the wavelength range 280-400 nm, without additional filters. The samples were placed in quartz J. Young NMR tubes and solved in deuterated cyclohexane (C_6D_{12}, 0.05-0.1 M) under argon before the irradiation. Upon irradiation the sample was cooled with compressed air and a fan to prevent thermal cycloreversion. The exact irradiation time was dependent on the composition/concentration of the solution. It varied between three and ten minutes.

Irradiation of ^{BN}B3

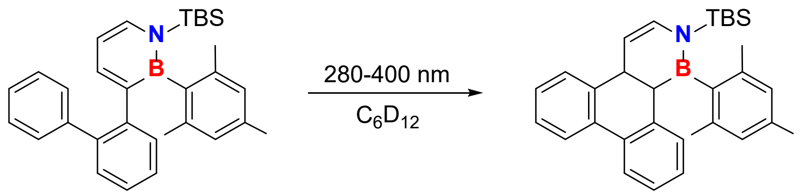

A 0.1 M solution of 3-(2-biphenylyl)-1-(tert-butyldimethylsilyl)-2-mesityl-1,2-dihydro-1,2-azaborinine (^{BN}**B3**) (23.2 mg, 50 µmol) in cyclohexane-d_{12} (0.5 mL) was prepared under inert atmosphere in a quartz J. Young NMR tube. The solution was irradiated for 5–10 min with a wavelength range of 280–400 nm while cooled with compressed air (T = 30–35 °C). The resulting mixture of the Dewar isomer (^{BN}**D3**) and the target compound (**2**) was then heated at 100 °C for 48–120 h and monitored by ^{1}H NMR spectroscopy to confirm complete back conversion of the Dewar isomer.

This cycle of irradiation and heating was repeated ten times, leading to 93–95% conversion to **2**. The crude product was purified by size-exclusion chromatography (*n*-hexane/DCM 75:25) to afford the product as a colorless oil (21.4 mg, 46 µmol, 92%).

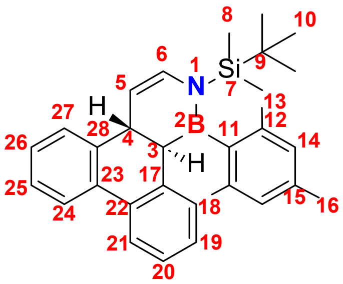

$C_{31}H_{38}BNSi$ (463.55 g/mol)

^{1}H-NMR (600 MHz, **C_6D_{12}**): δ = 7.67 (m, 2H, H-21/H-24), 7.48 (dm, $^{3}J_{HH}$ = 7.34 Hz, 1H, H-27), 7.20 (m, 2H, H-25/H-26), 6.99 (ps.t, 1H, H-20), 6.80 (s, 1H, H-14), 6.71 (s, 1H, H-14),

6.63 (m, 2H, H-18/H19), 6.51 (dd, $^3J_{HH}$ = 7.71 Hz, $^4J_{HH}$ = 2.81Hz, 1H, H-6), 6.08 (dd, $^3J_{HH}$ = 7.71 Hz, $^4J_{HH}$ = 2.74 Hz, 1H, H-5), 3.68 (dm, $^3J_{HH}$ = 18.01 Hz, 1H, H-4), 2.62 (dm, $^3J_{HH}$ = 18.01 Hz, 1H, H-3), 2.31 (s, 3H, H-16), 2.23 (s, 3H, H-13), 2.00 (s, 3H, H-13), 0.97 (s, 9H, H-10), 0.08 (s, 3H, H-8), -0.34 (s, 3H, H-8) ppm.

^{13}C-{^{1}H}-NMR (151 MHz, **C_6D_{12}**): δ = 141.7, 138.1, 137.8, 137.3, 137.1, 136.9, 134.6, 134.4, 131.8, 127.1, 127.0, 126.5, 126.3, 126.0, 125.0, 123.8, 123.7, 123.0, 114.9, 34.9, 33.3, 27.3, 22.7, 21.7, 20.6, 19.2, -4.2, -4.6 ppm.

^{11}B-{^{1}H}-NMR (193 MHz, **C_6D_{12}**): δ = 51.6 ppm.

HRMS (ESI) m/z: $[M + H]^+$ Calcd for $C_{31}H_{39}BNSi$ 464.2939; Found 464.2948.

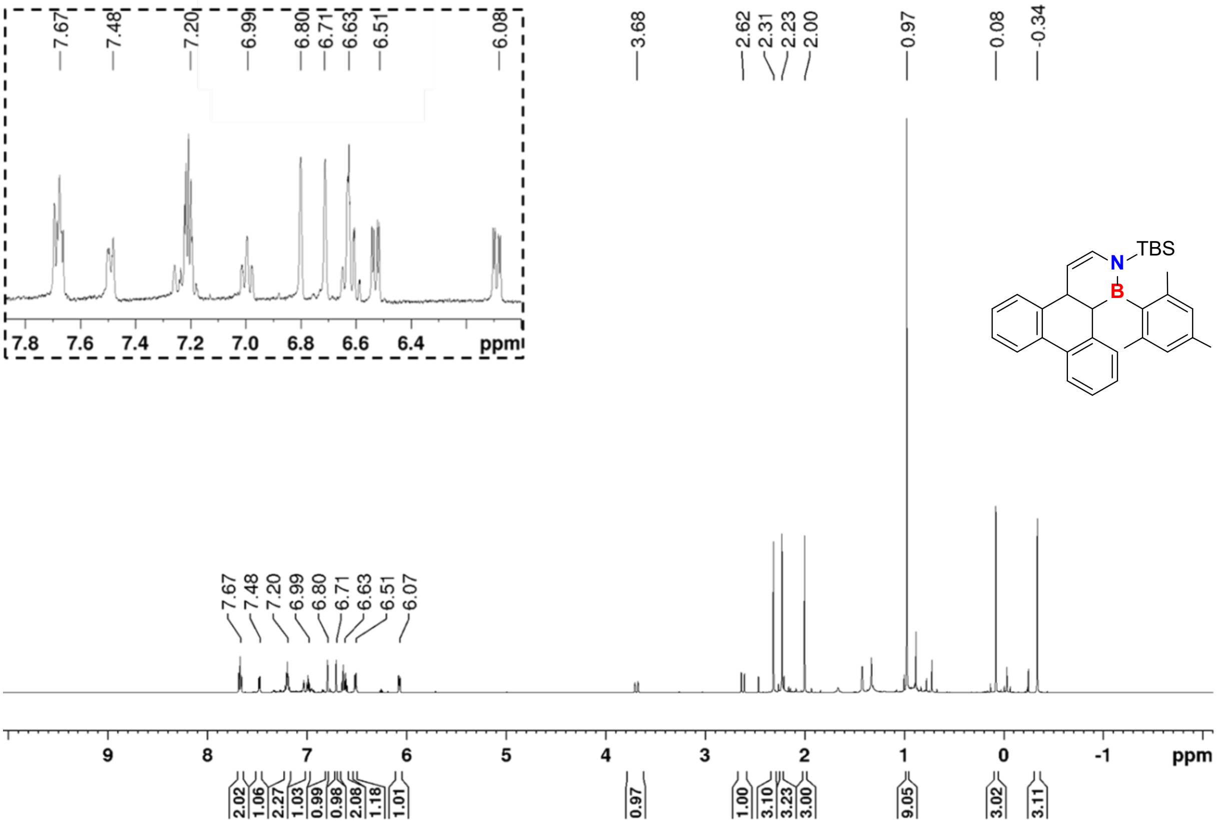

Figure S19. ^{1}H NMR spectrum of compound **2** in C_6D_{12} measured on a 600 MHz spectrometer.

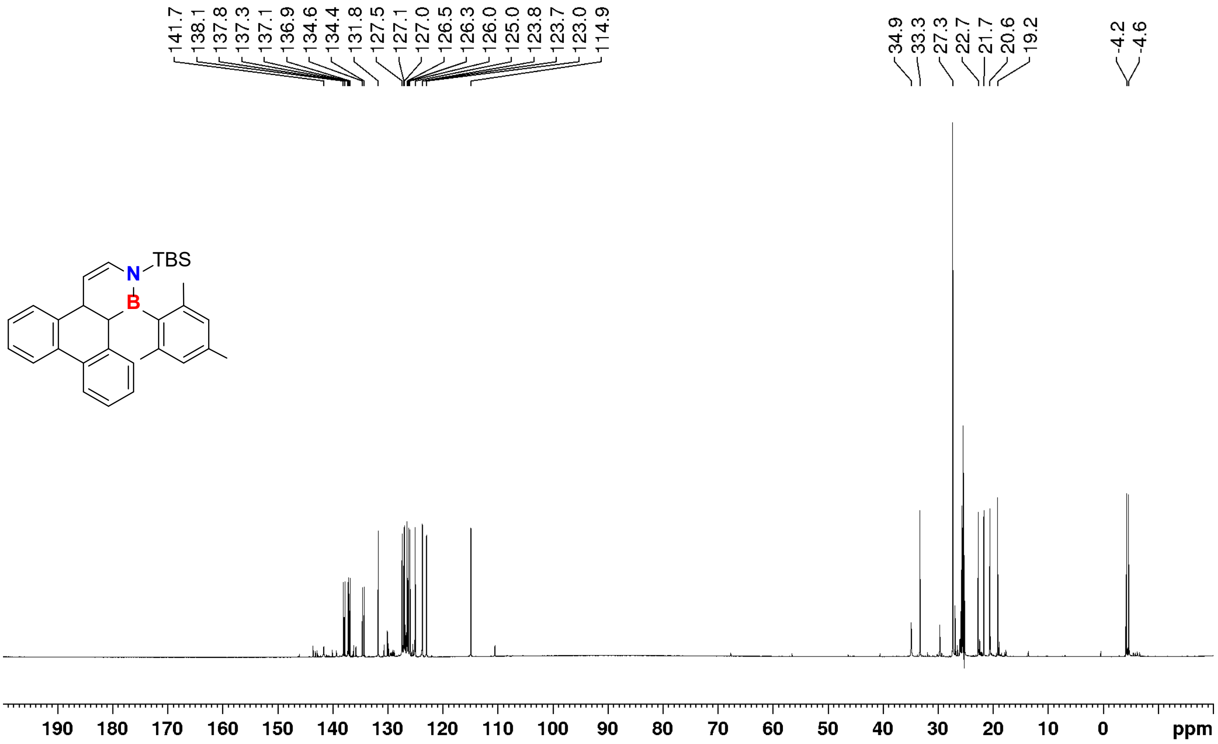

Figure S20. ^{13}C-{^{1}H} NMR spectrum of compound **2** in C_6D_{12} measured on a 600 MHz spectrometer.

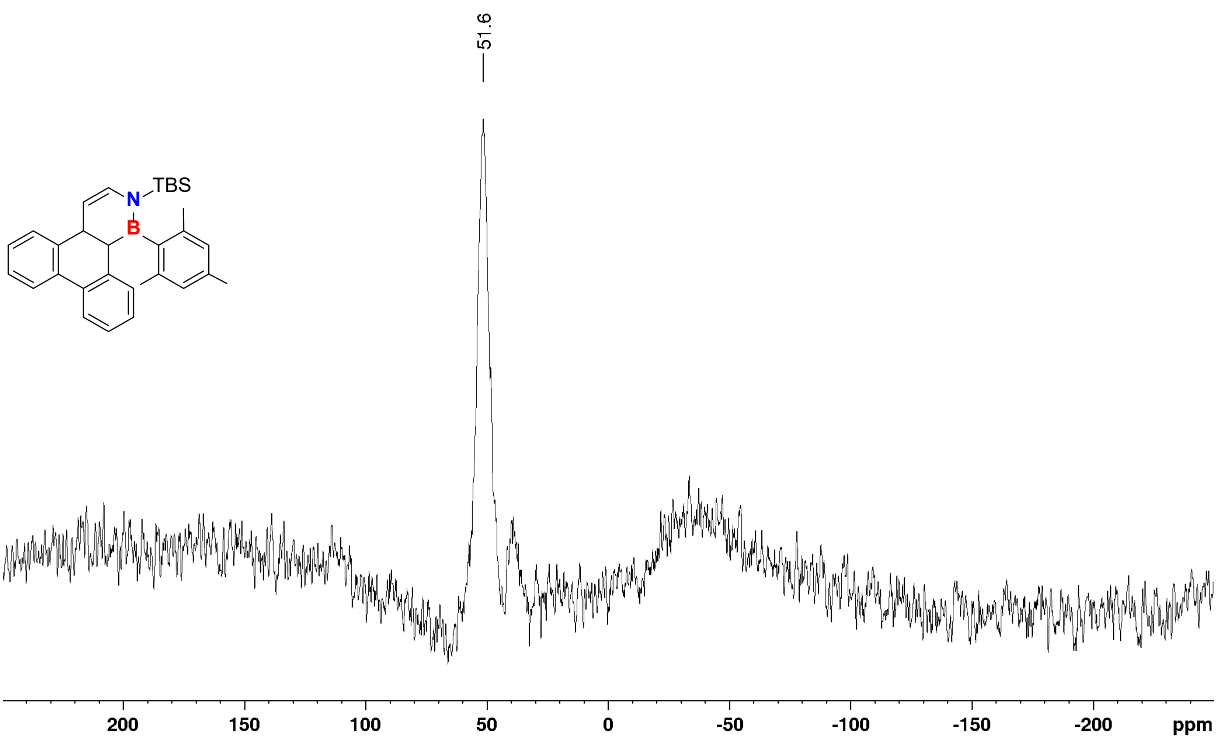

Figure S21. ^{11}B-{^{1}H} NMR spectrum of compound **2** in C_6D_{12} measured on a 600 MHz spectrometer.

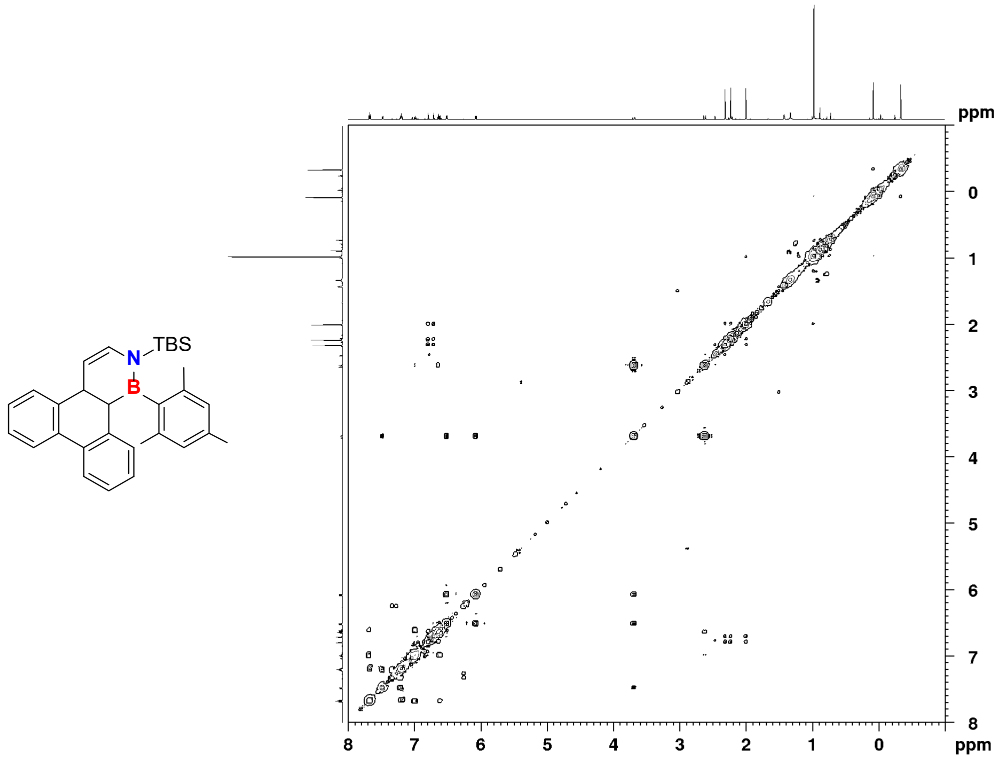

Figure S22. ^{1}H-^{1}H-COSY NMR spectrum of compound **2** in C_6D_{12} measured on a 600 MHz spectrometer.

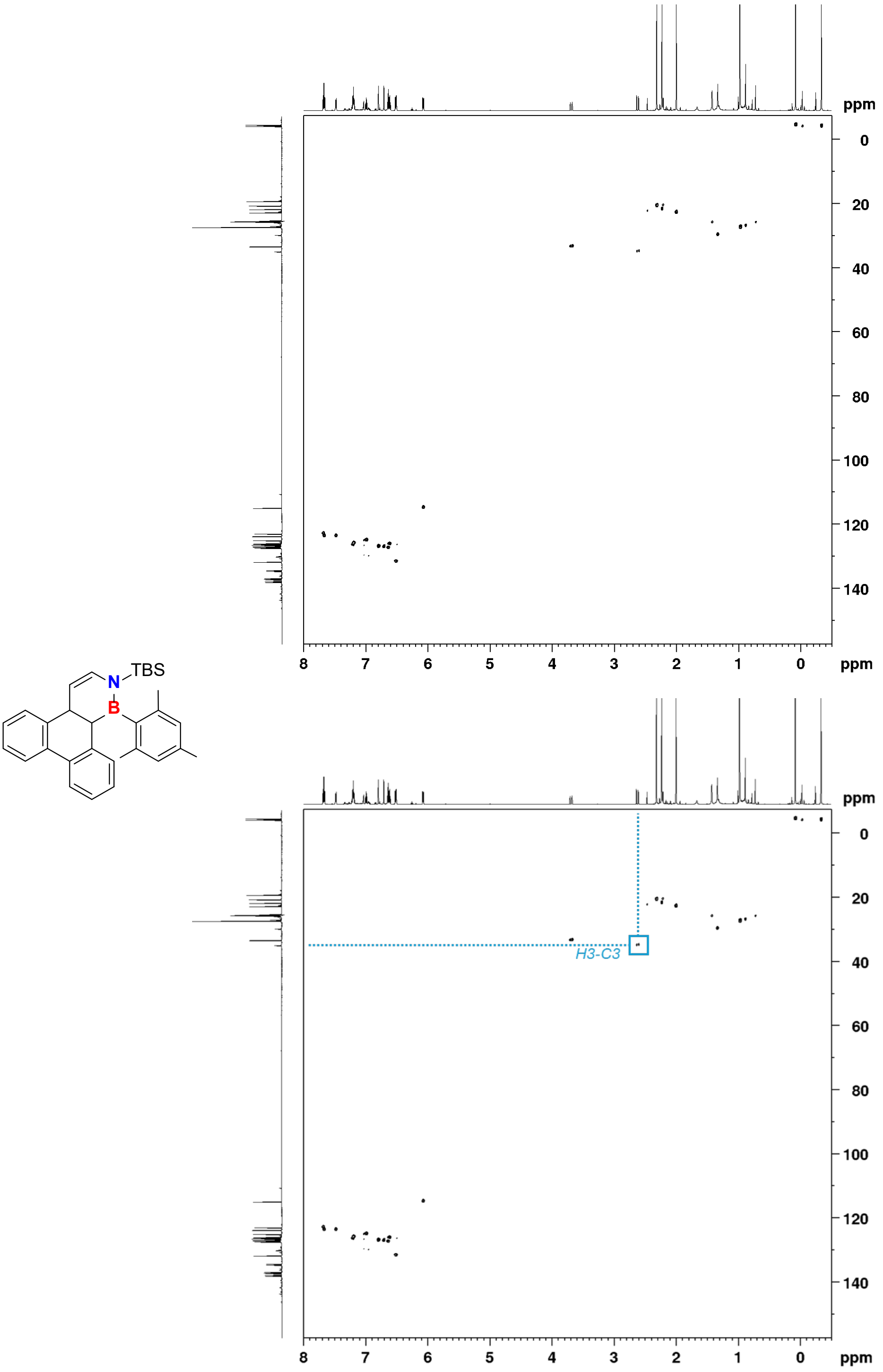

Figure S23. ^{1}H-^{13}C-HSQC NMR spectrum of compound **2** in C_6D_{12} measured on a 600 MHz spectrometer. Highlighted in light blue is the HSQC coupling of the characteristically broadened C3 signal (broadening caused by the quadrupole moment of the adjacent ^{11}B atom), which demonstrates that this carbon is no longer quaternary after irradiation (bottom).

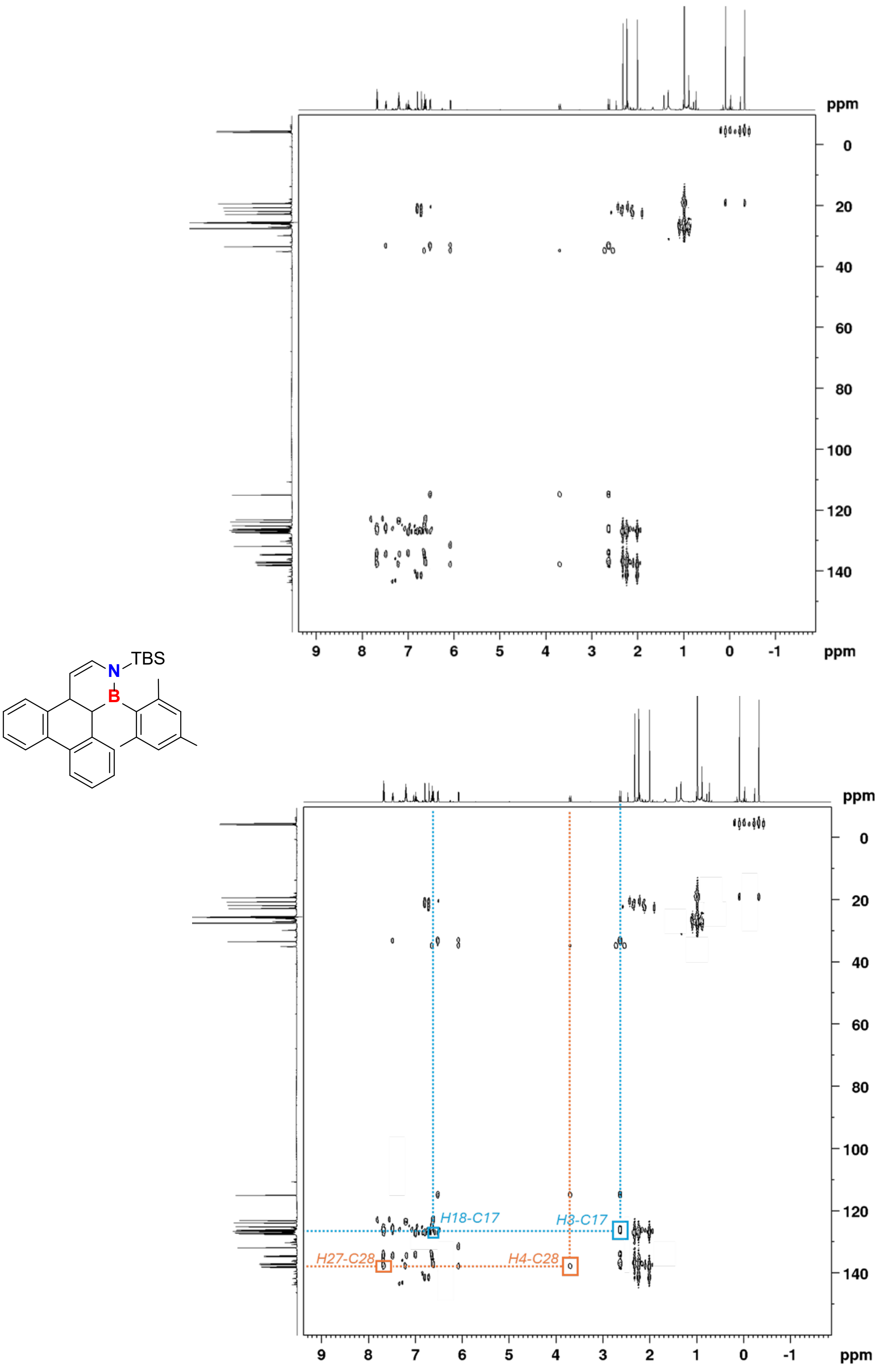

Figure S24. ^{1}H-^{13}C-HMBC NMR spectrum of compound **2** in C_6D_{12} measured on a 600 MHz spectrometer. Highlighted are the HMBC couplings characteristic between H-3/H-18 to the quaternary carbon C17 and from H-4/H-27 to the quaternary carbon C28. These correlations confirm the formation of a bond between C4 and C28 while the C3-C17 bond is retained (bottom).

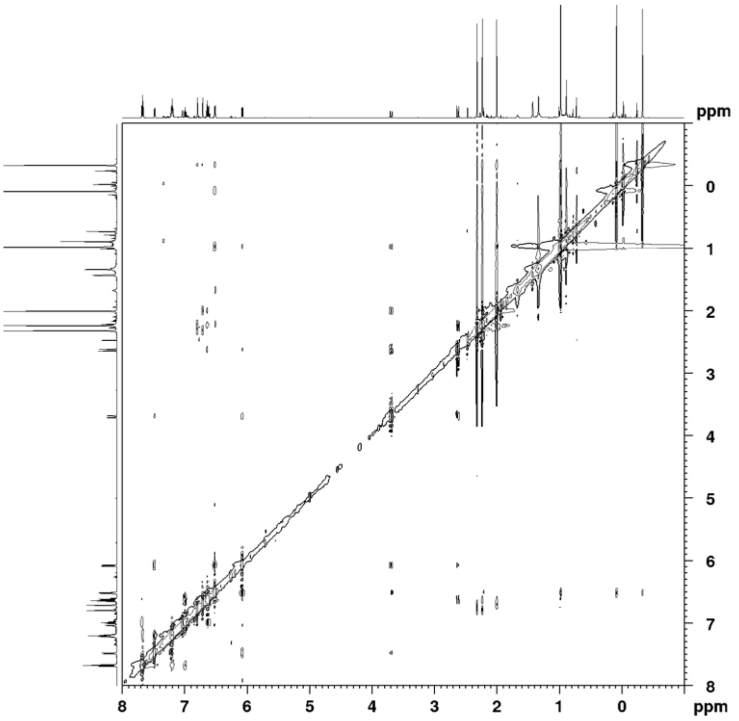

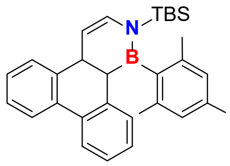

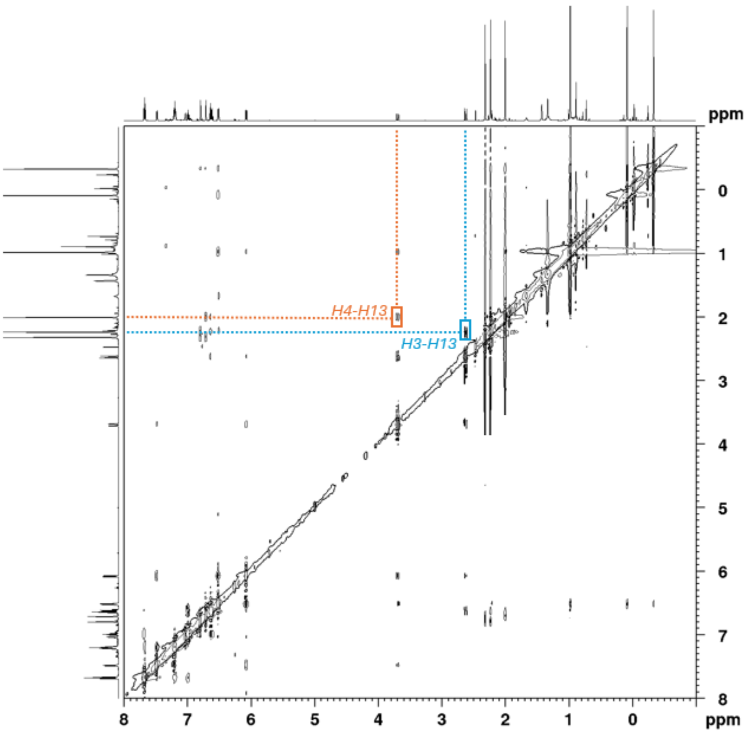

Figure S25. ^{1}H-^{1}H-NOESY NMR spectrum of compound **2** in C_6D_{12} measured on a 600 MHz spectrometer. Highlighted are the NOESY couplings between H-3/H-4 and one of the two *ortho* methyl groups (C13) of the mesityl substituent at the boron atom (bottom).

Irradiation of ^{BN}B4

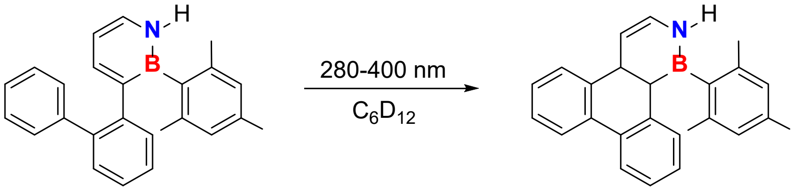

A 0.05 M solution of 3-(2-biphenylyl)-2-mesityl-1,2-dihydro-1,2-azaborinine (8.7 mg, 25 µmol) in cyclohexane-d_{12} (0.5 mL) was prepared under inert atmosphere in a quartz J. Young NMR tube. The solution was irradiated for 3 min with a wavelength range of 280-400 nm while cooled with compressed air (T = 30-35 °C). The crude product was purified by size-exclusion chromatography (*n*-hexane/DCM 50:50) to afford the product as a colorless oil (7.8 mg, 22.4 µmol, 92%).

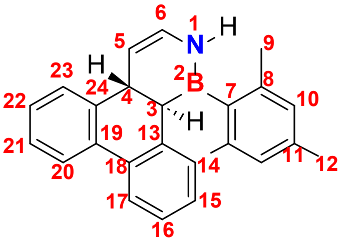

$C_{23}H_{24}BN$ (349.28 g/mol)

^{1}H-NMR (400 MHz, **C_6D_{12}**): δ = 7.63 (m, 2H, H-17/H-20), 7.47 (m, 1H, H-23), 7.20 (m, 3H, H-14/H-15/H-21), 7.05 (ps. t, 1H, H-16), 6.79 (s, 1H, H-10), 6.75 (ps.t, 1H, H-22), 6.74 (s, 1H, H-10), 6.23 (m, 1H, H-6), 5.70 (d, $^3J_{HH}$ = 8.66 Hz, 1H, H-5), 5.42 (br. S, 1H, H-1), 3.69 (dm, $^3J_{HH}$ = 18.53 Hz, 1H, H-4), 2.82 (d, $^3J_{HH}$ = 18.53 Hz, 1H, H-3), 2.27 (s, 3H, H-12), 2.20 (s, 3H, H-9), 2.11 (s, 3H, H-9) ppm.

^{13}**C-{^{1}H}-NMR** (128 MHz, **C_6D_{12}**): δ = 140.1, 139.9, 138.6, 137.7, 137.2, 136.6, 135.2, 135.0, 127.4, 127.1, 126.8, 126.6, 126.5, 126.0, 125.6, 124.2, 123.7, 123.1, 108.4, 35.5, 33.1, 22.1, 21.2, 20.5 ppm.

^{11}**B-{^{1}H}-NMR** (128 MHz, **C_6D_{12}**): δ = 46.8 ppm.

HRMS (ESI) m/z: $[M + H]^+$ Calcd for $C_{23}H_{25}BN$ 350.2075; Found 350.2076.

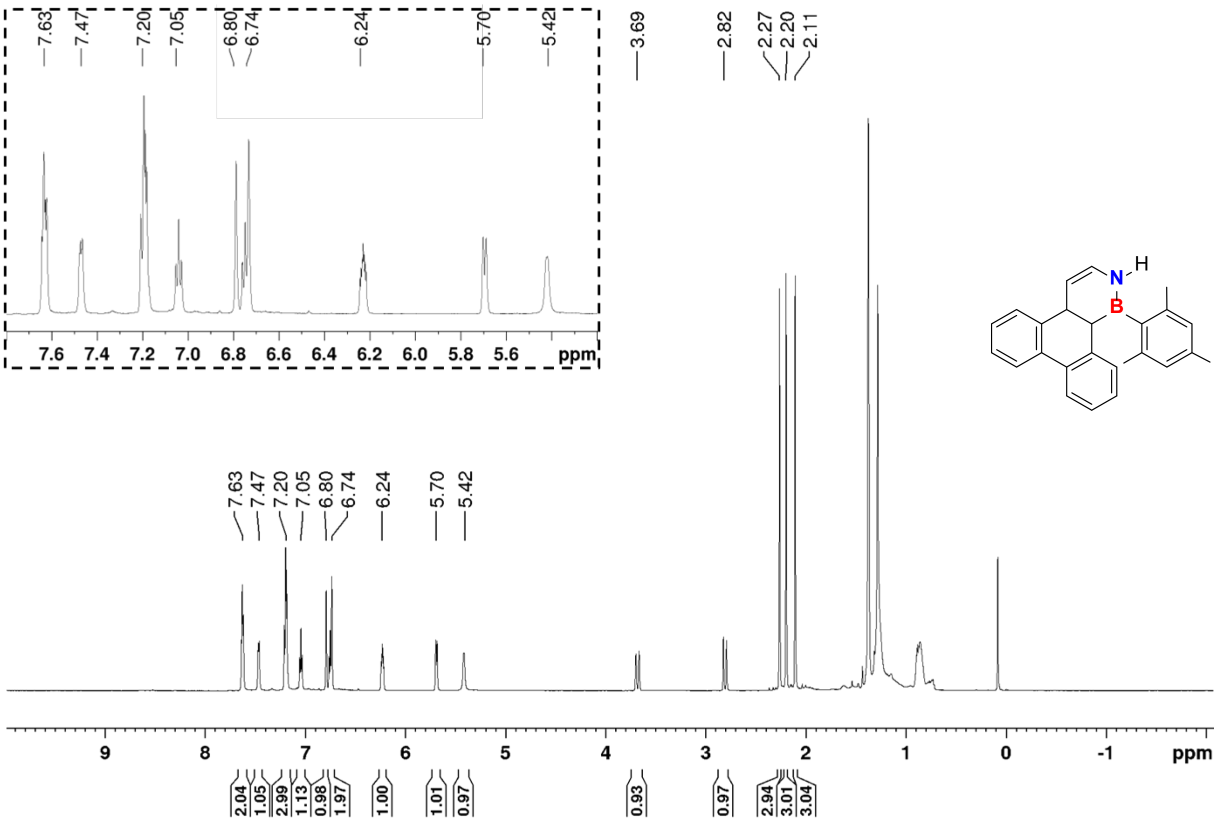

Figure S26. ^{1}H NMR spectrum of compound **3** in C_6D_{12} measured on a 600 MHz spectrometer.

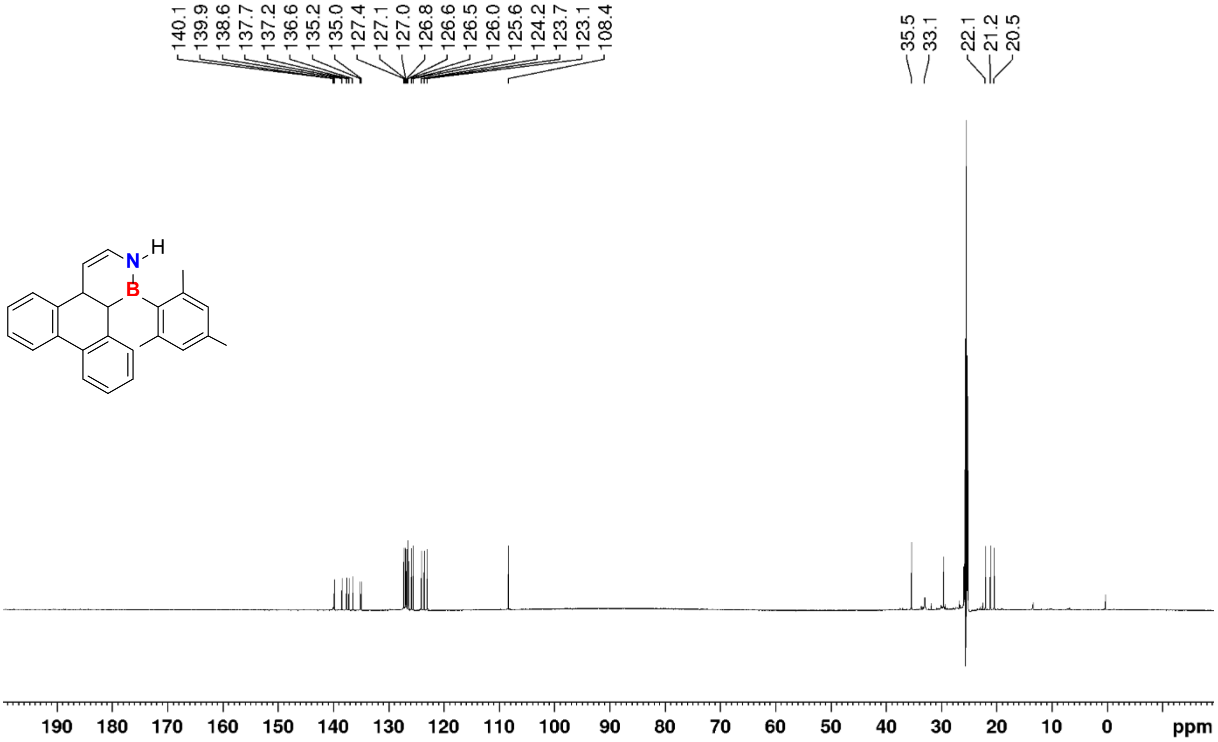

Figure S27. ^{13}C-{^{1}H} NMR spectrum of compound **3** in C_6D_{12} measured on a 600 MHz spectrometer.

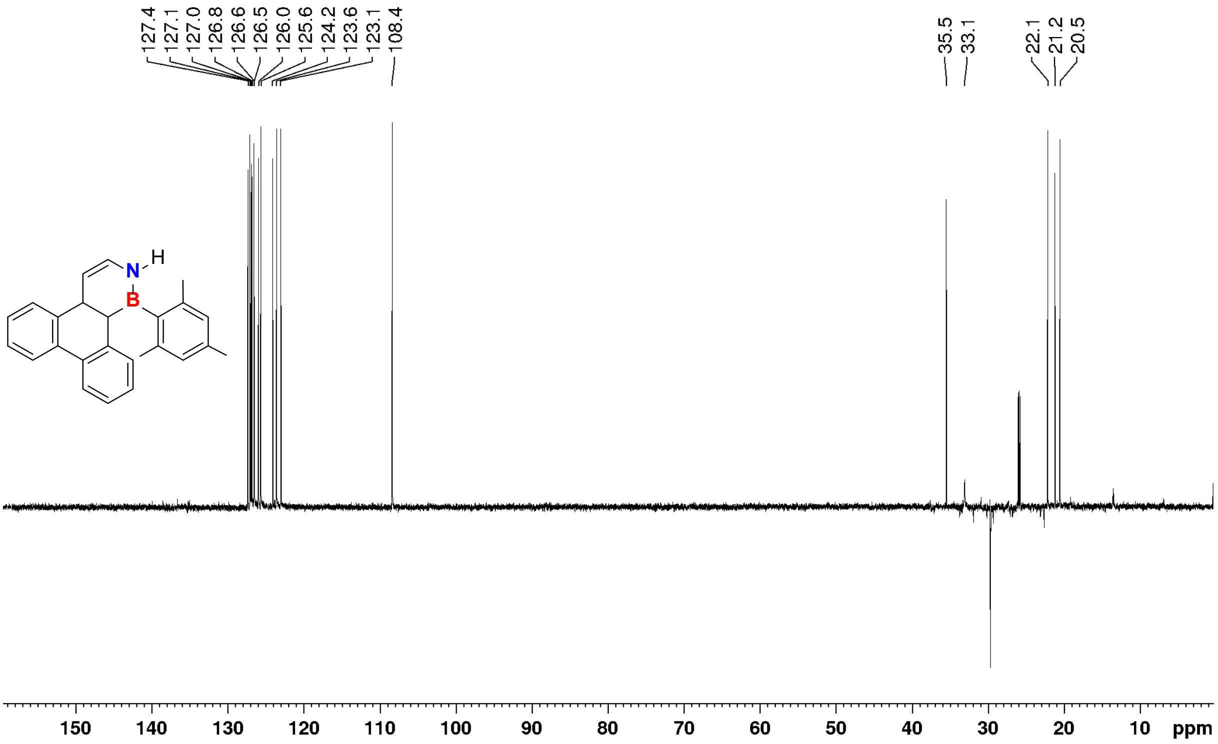

Figure S28. ^{13}C-DEPT90 NMR spectrum of compound **3** in C_6D_{12} measured on a 600 MHz spectrometer.

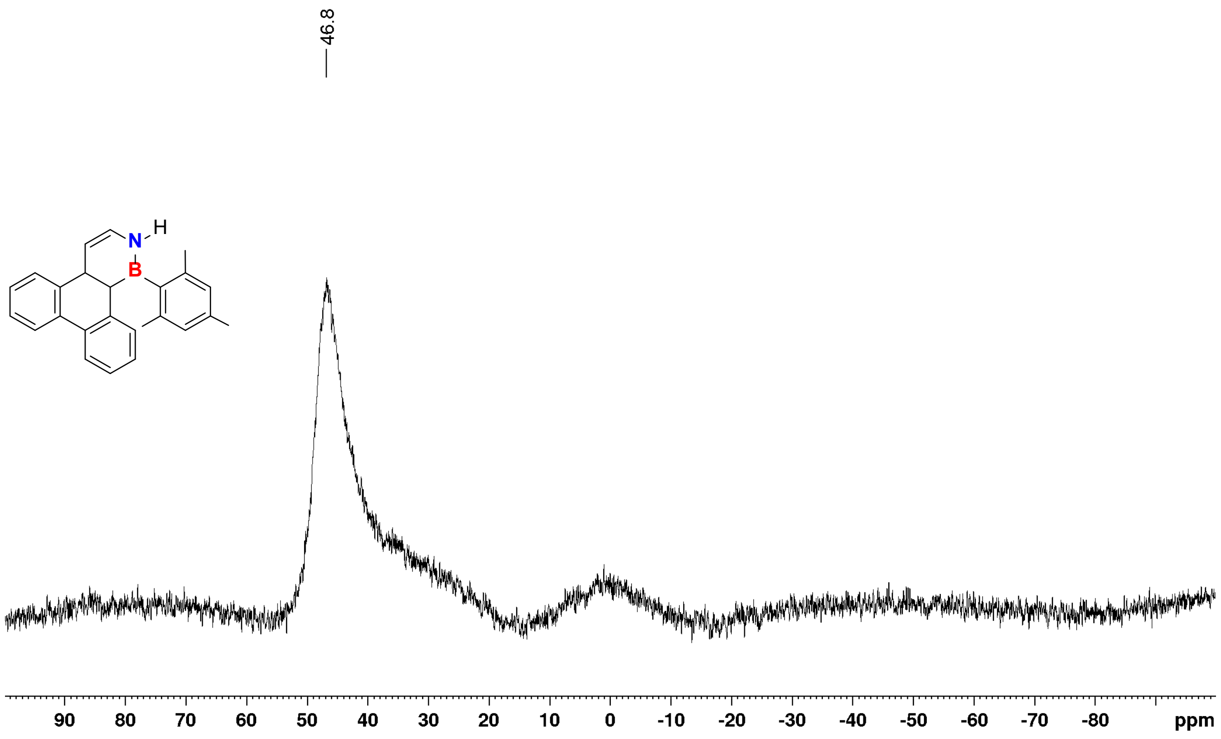

Figure S29. ^{11}B-{^{1}H}-NMR spectrum of compound **3** in C_6D_{12} measured on a 600 MHz spectrometer.

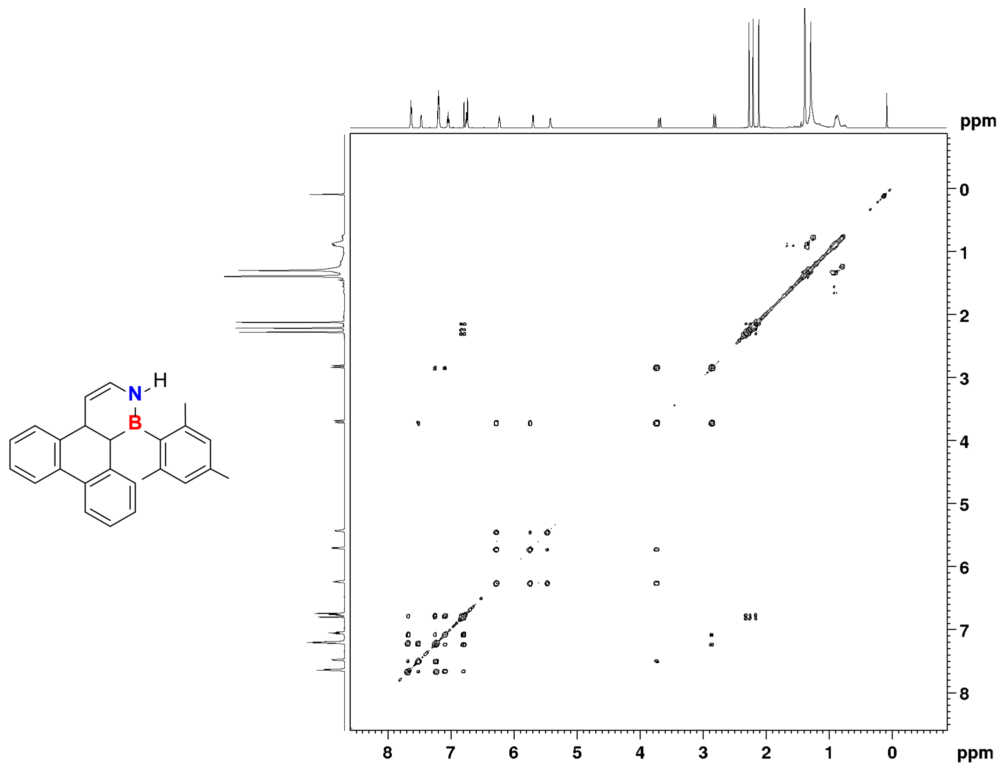

Figure S30. ^{1}H-^{1}H-COSY NMR spectrum of compound **3** in C_6D_{12} measured on a 600 MHz spectrometer.

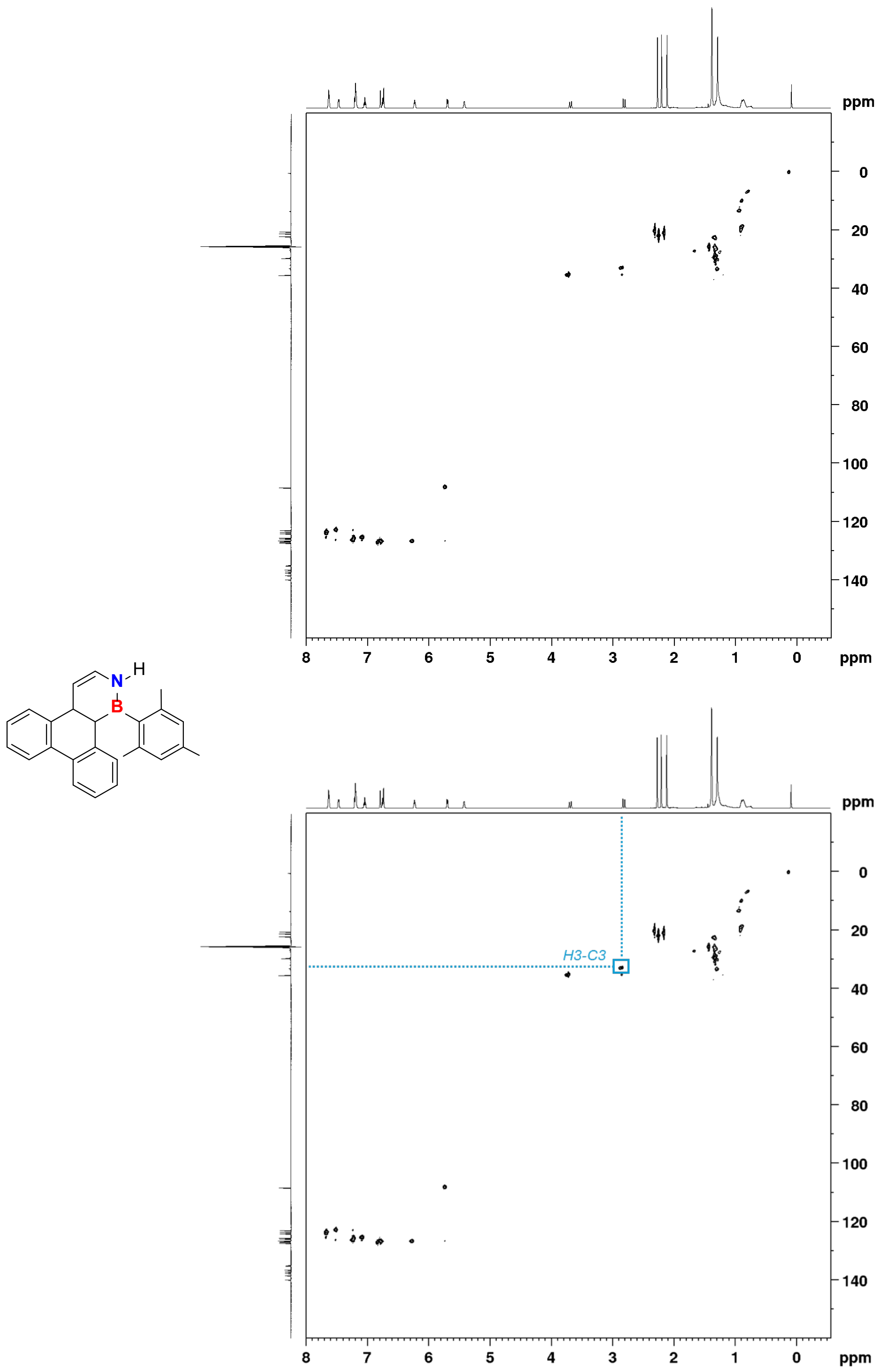

Figure S31. ^{1}H-^{13}C-HSQC NMR spectrum of compound **3** in C_6D_{12} measured on a 600 MHz spectrometer. Highlighted in light blue (bottom) is the HSQC coupling of the characteristically broadened C3 signal (broadening caused by the quadrupole moment of the adjacent ^{11}B atom), which demonstrates that this carbon is no longer quaternary after irradiation.

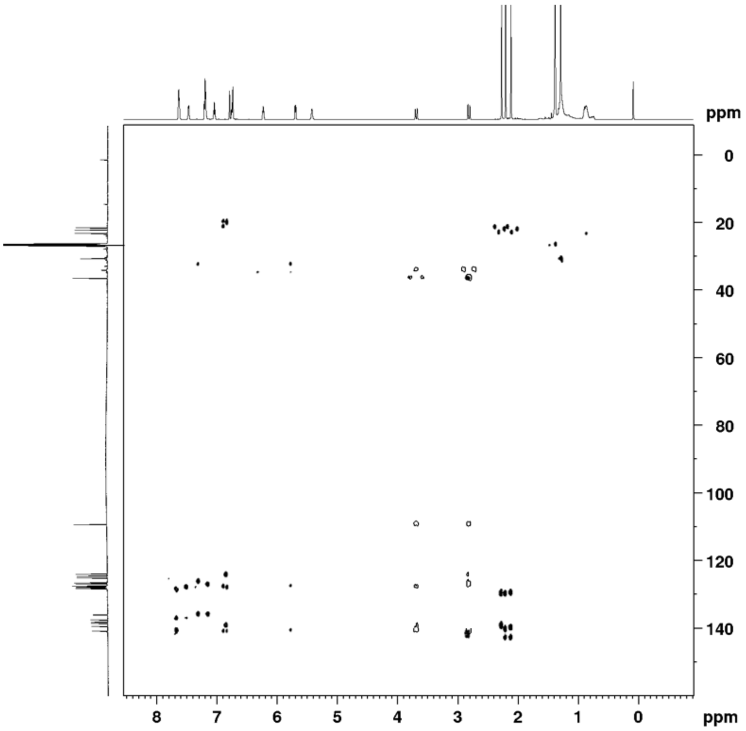

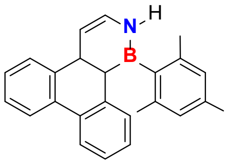

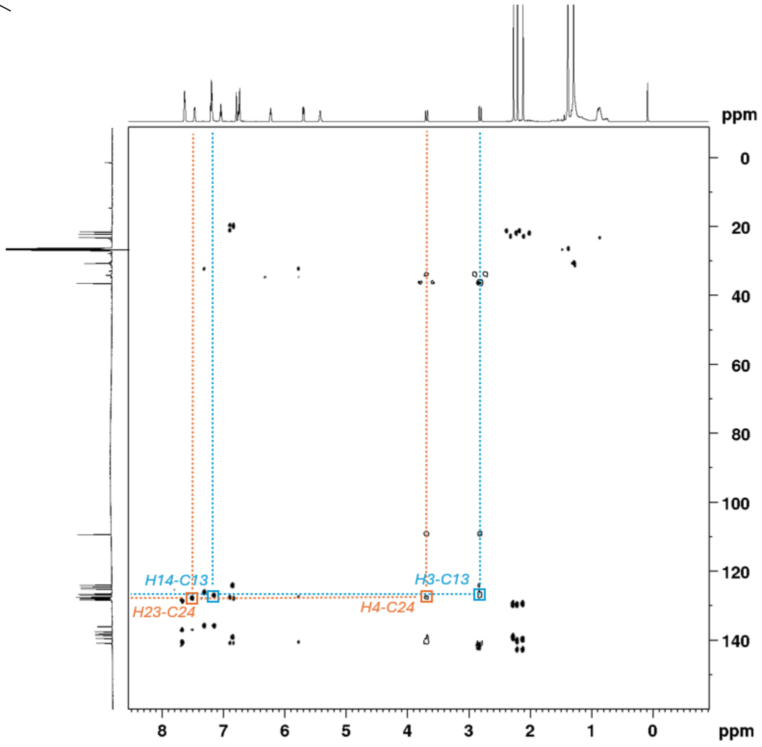

Figure S32. ^{1}H-^{13}C-HMBC NMR spectrum of compound **3** in C_6D_{12} measured on a 600 MHz spectrometer. Highlighted are the HMBC couplings characteristic between H-3/H-14 to the quaternary carbon C13 and from H-4/H-23 to the quaternary carbon C24. These correlations confirm the formation of a bond between C4 and C24 while the C3-C13 bond is retained (bottom).

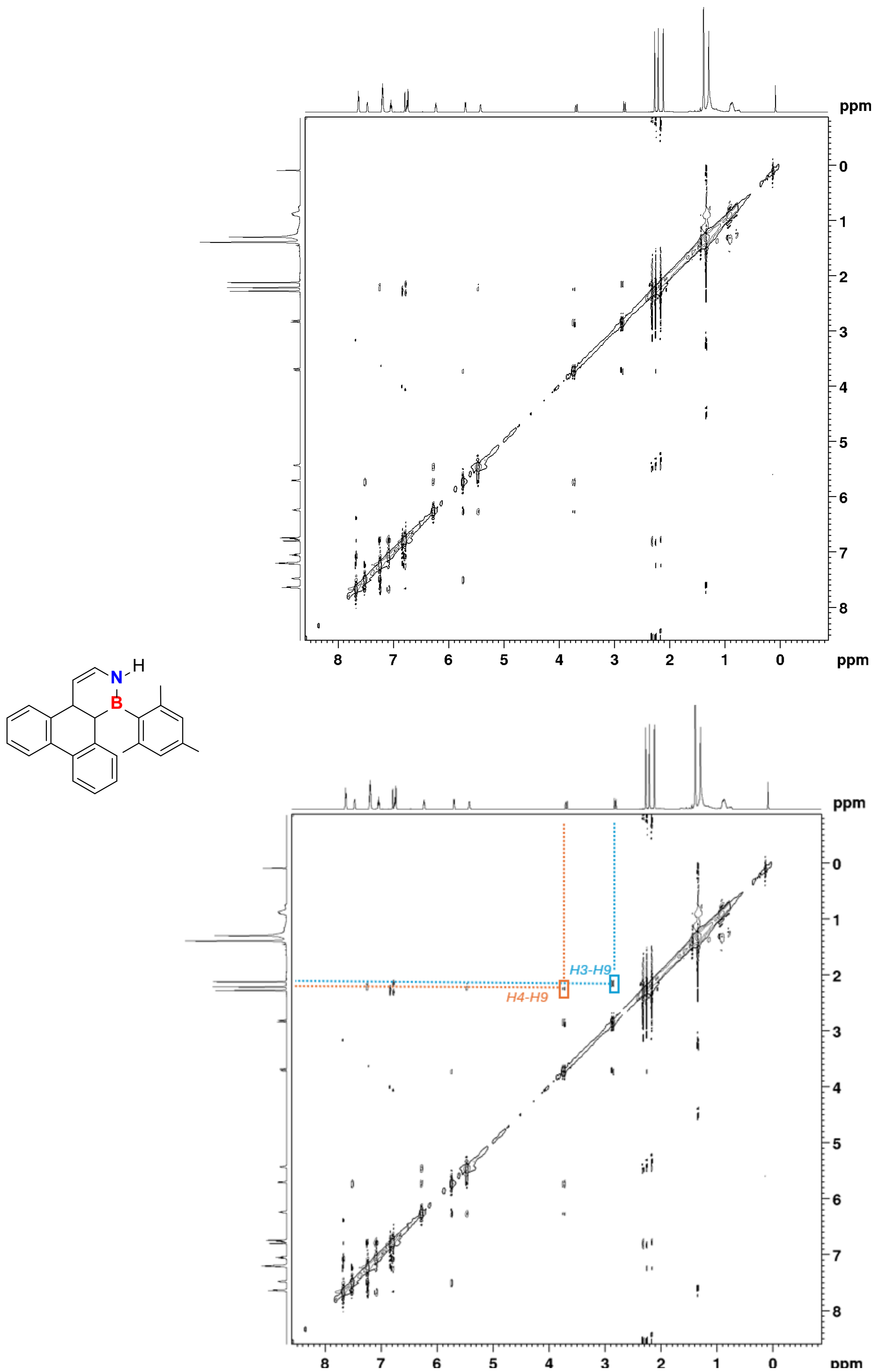

Figure S33. ^{1}H-^{1}H-NOEY NMR spectrum of compound **3** in C_6D_{12} measured on a 600 MHz spectrometer. Highlighted are the NOESY couplings between H-3/H-4 and one of the two *ortho* methyl groups (C9) of the mesityl substituent at the boron atom (bottom).

Synthesis of compound 4

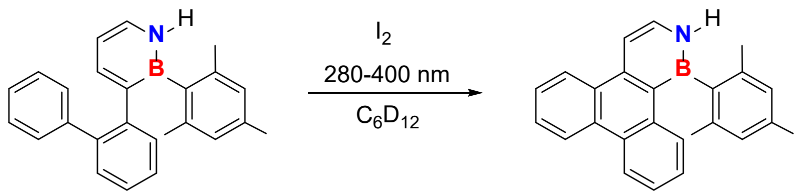

Route A: Irradiation of ^{BN}**B4** in the presence of iodine

A solution of 2-mesityl-3-(2-biphenylyl)-1,2-dihydro-1,2-azaborinine ^{BN}**B4** (88 mg, 0.25 mmol) in cyclohexane-d_{12} (5 mL) was prepared under inert atmosphere in a quartz Schlenk tube and iodine (66 mg, 0.26 mmol) was added. The solution was irradiated for 3 min with a wavelength range of 280-400 nm while cooled with compressed air (T = 30-35 °C). The crude product was purified by column chromatography (*n*-hexane/DCM 50:50) to afford the product (**4**) as a colorless oil (40 mg, 0.12 mmol, 46%).

Route B: Oxidation of compound **2**

Compound **4** can be obtained not only by direct irradiation of ^{BN}**B4** in the presence of iodine, but also by reacting **3** with iodine. For this purpose, a solution of **3** (5 mg, 14.2 µmol) in cyclohexane-d_{12} (0.5 mL) is prepared in a Quartz glas J.-Young NMR tube and iodine (7.2 mg, 28.4 µmol) is added. The solution is either irradiated with 280-400 nm light for 3-5 min or heated in an oil bath at 60 °C for 16 h in the dark. In both cases, the crude product is purified by column chromatography (*n*-hexane/DCM 50/50). Under irradiation 2.5 mg (7.2 µmol, 51%) of **4** are obtained, whereas heating affords 2.9 mg (8.5 µmol, 60%) of **4**.

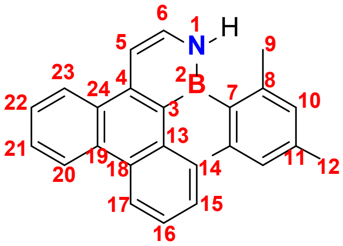

$C_{23}H_{22}BN$ (347.27 g/mol)

^{1}H-NMR (400 MHz, **C_6D_{12}**): δ = 8.65 (d, $^{3}J_{HH}$ = 8.32 Hz, 1H, H-23), 8.54 (d, $^{3}J_{HH}$ = 8.36 Hz, 2H, H-17/H-20), 8.30 (d, $^{3}J_{HH}$ = 8.32 Hz, 1H, H-14), 7.58 (m, 2H, H-5/H-22), 7.53 (t, $^{3}J_{HH}$ = 8.35 Hz, 2H, H-21), 7.48 (br. s, 1H, H-1), 7.36 (m, 2H, H-6/H-16), 7.08 (t, $^{3}J_{HH}$ = 8.35 Hz, 1H, H-15), 6.86 (s, 2H, H-10), 2.34 (s, 3H, H-12), 2.05 (s, 6H, H-9) ppm.

^{13}C-{^{1}H}-NMR (128 MHz, **C_6D_{12}**): δ = 142.7, 141.5, 139.5, 137.7, 137.5, 136.8, 133.5, 131.3, 130.4, 129.5, 128.5, 128.4, 128.1, 127.5, 127.2, 126.6, 126.0, 124.8, 123.6, 122.8, 107.1, 22.7, 21.6 ppm.

^{11}B-{^{1}H}-NMR (128 MHz, **C_6D_{12}**): δ = 36.7 ppm.

HRMS (ASAP) m/z: $[M + H]^{+}$ Calcd for $C_{23}H_{23}BN$ 348.1918; Found 348.1921.

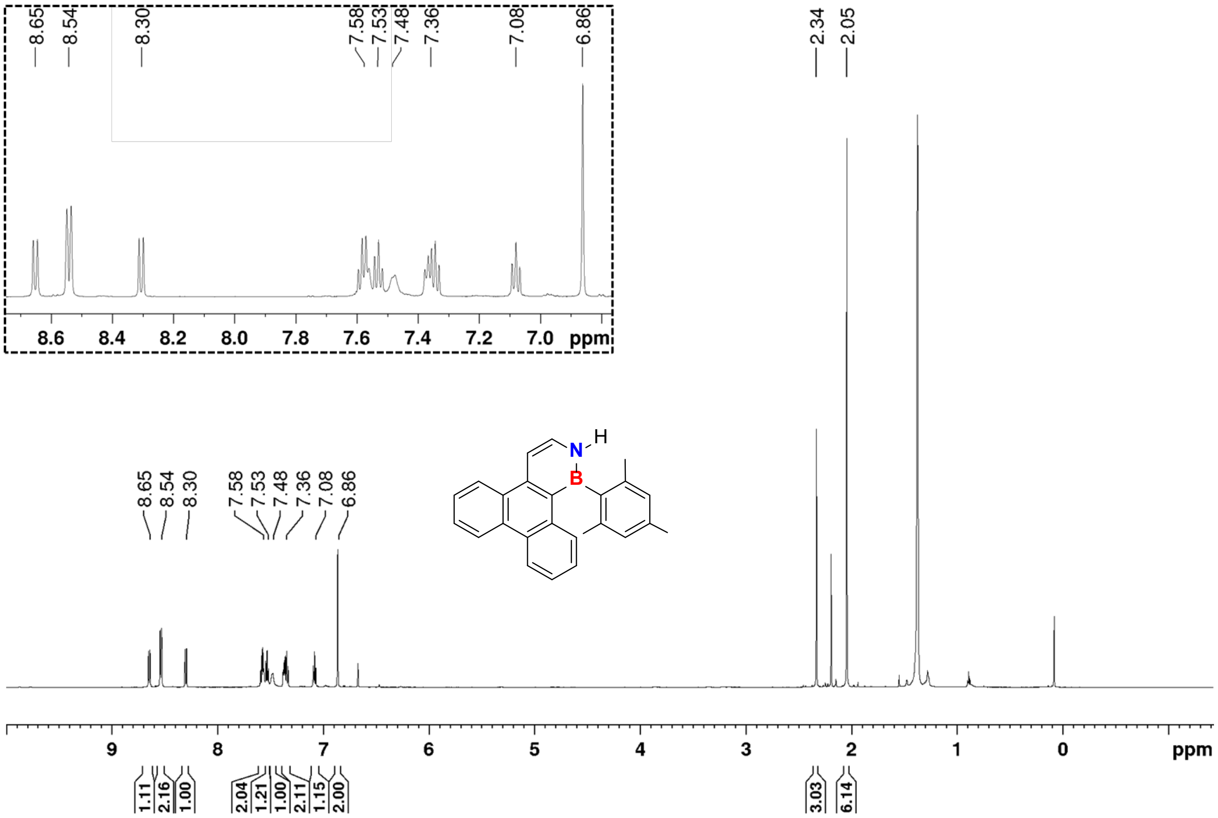

Figure S34. ^{1}H NMR spectrum of compound **4** in C_6D_{12} measured on a 600 MHz spectrometer.

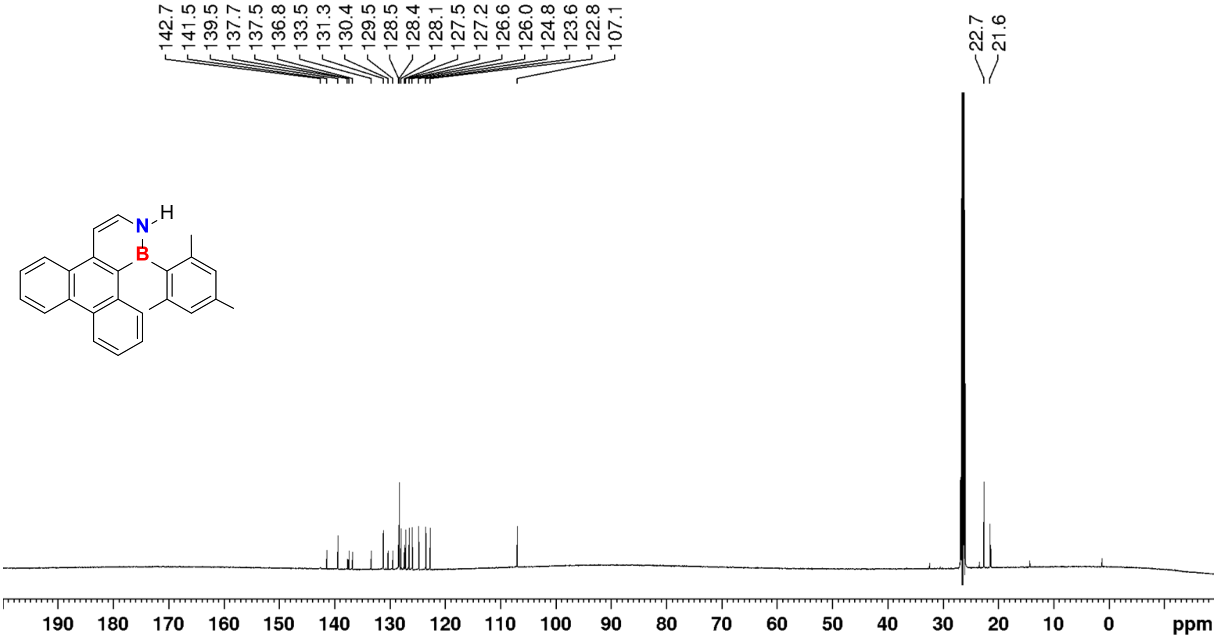

Figure S35. ^{13}C-{^{1}H} NMR spectrum of compound **4** in C_6D_{12} measured on a 600 MHz spectrometer.

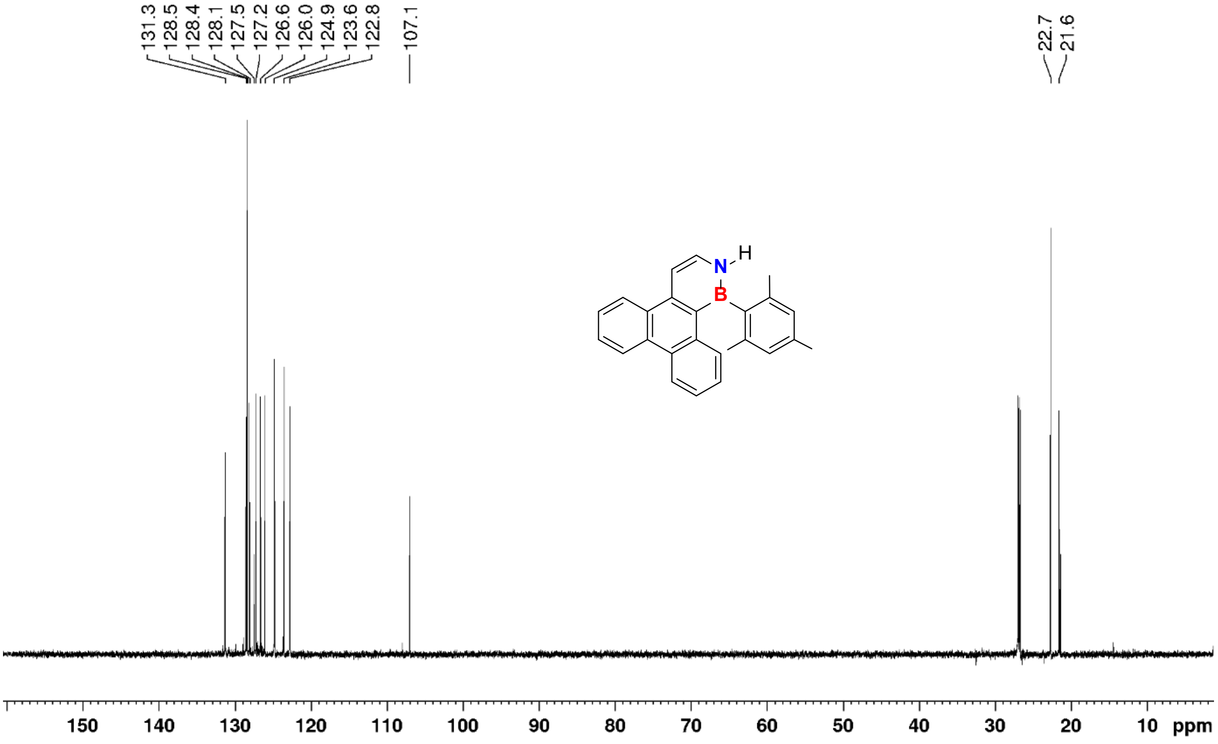

Figure S36. ^{13}C-DEPT90 NMR spectrum of compound **4** in C_6D_{12} measured on a 600 MHz spectrometer.

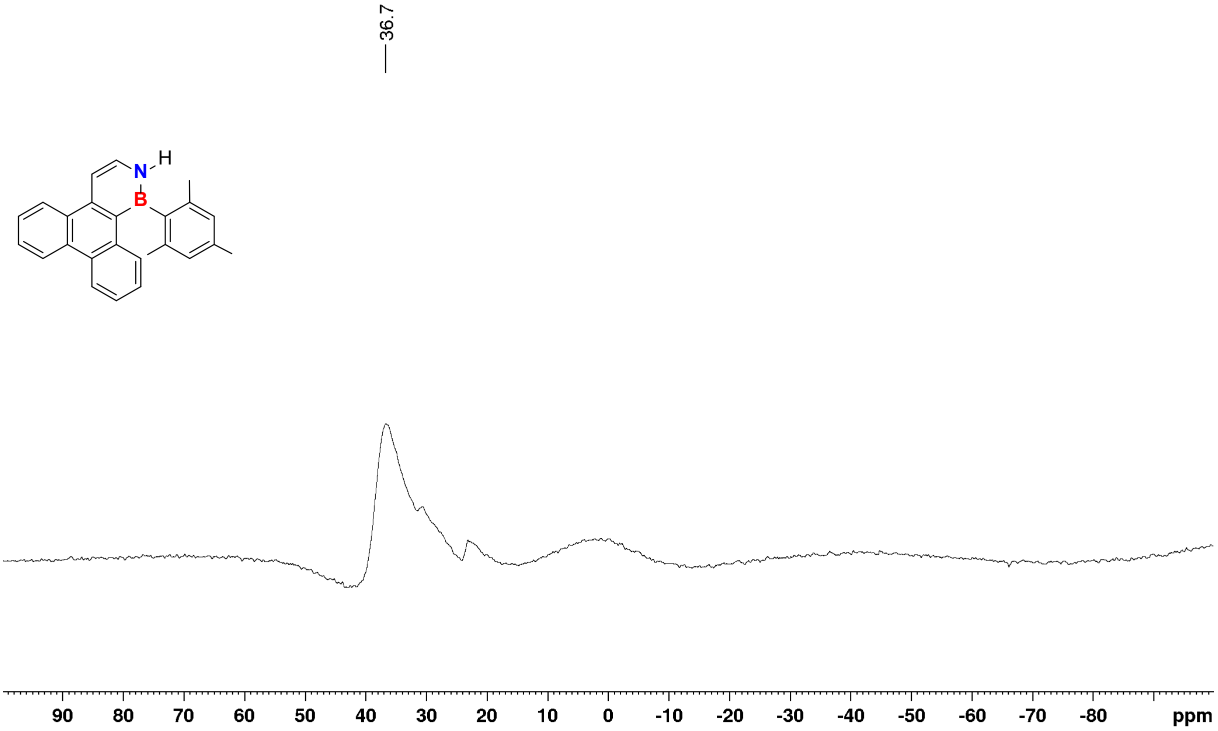

Figure S37. ^{11}B-{^{1}H}-NMR spectrum of compound **4** in C_6D_{12} measured on a 600 MHz spectrometer.

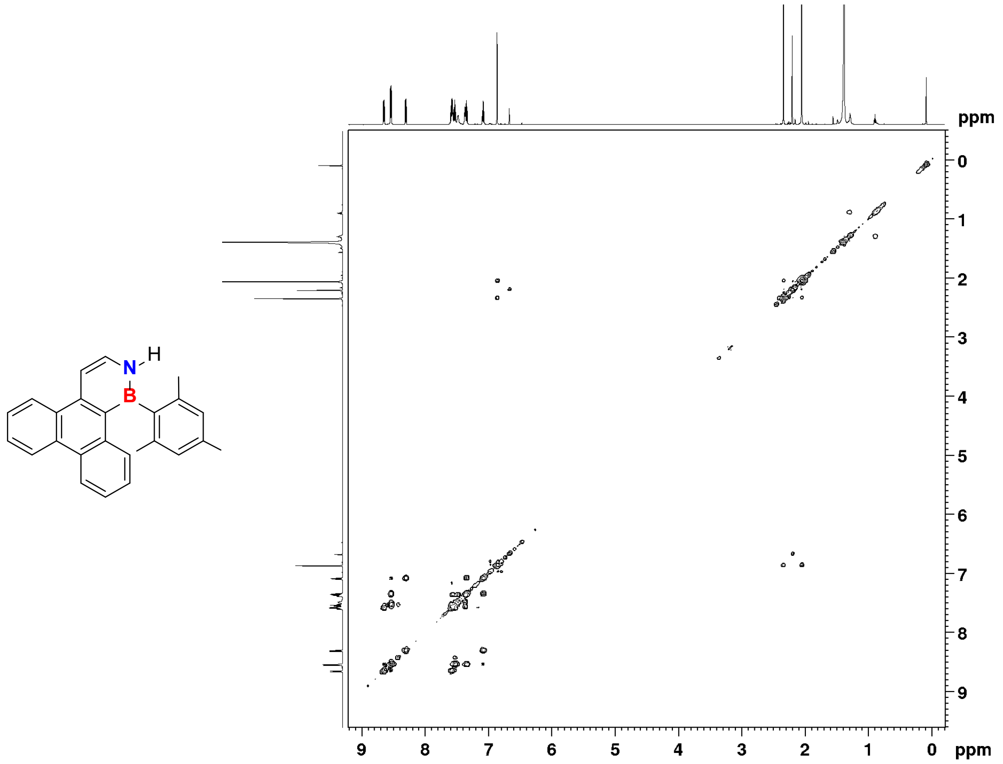

Figure S38. ^{1}H-^{1}H-COSY NMR spectrum of compound **4** in C_6D_{12} measured on a 600 MHz spectrometer.

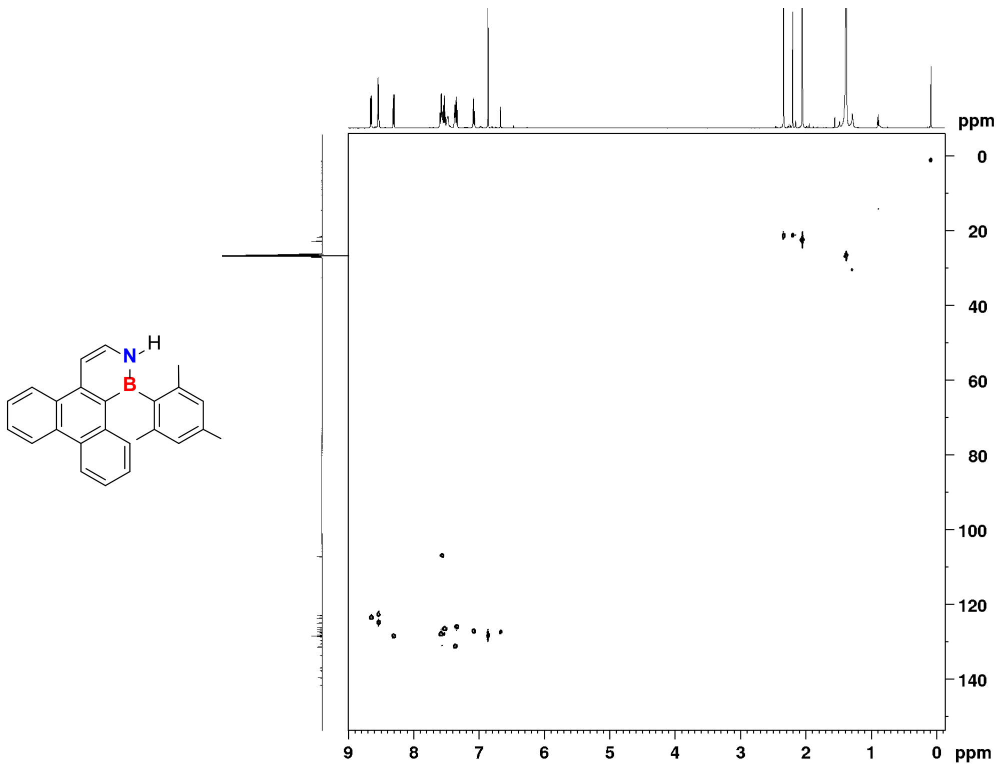

Figure S39. ^{1}H-^{13}C-HSQC NMR spectrum of compound **4** in C_6D_{12} measured on a 600 MHz spectrometer.

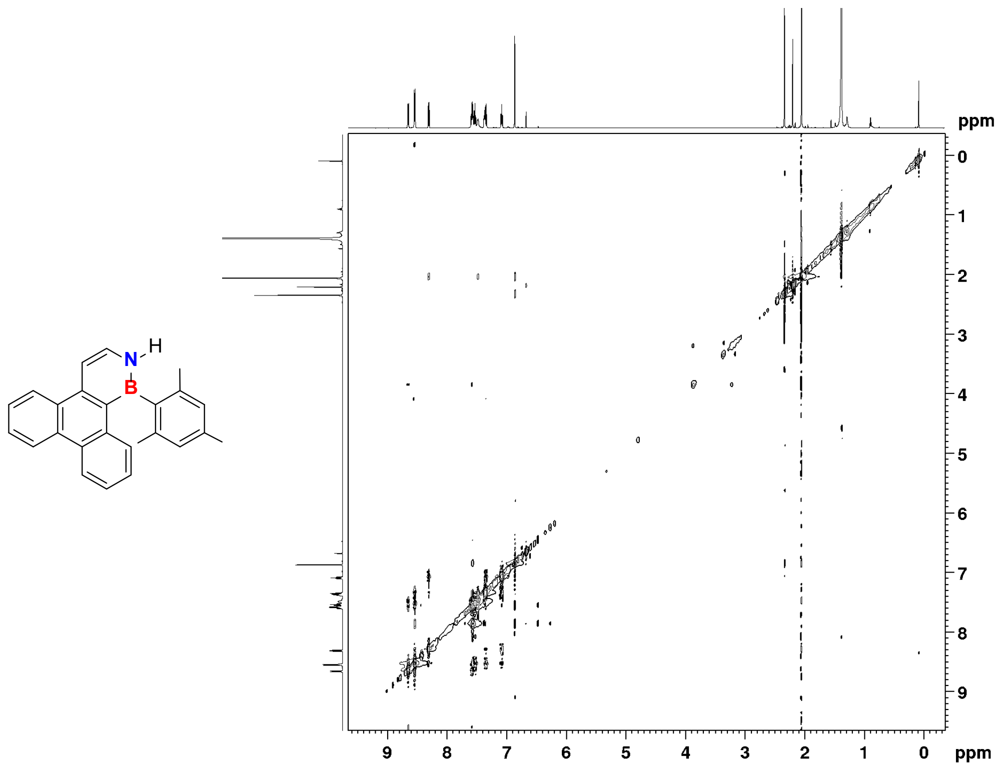

Figure S40. ^{1}H-^{1}H-NOESY NMR spectrum of compound **4** in C_6D_{12} measured on a 600 MHz spectrometer.

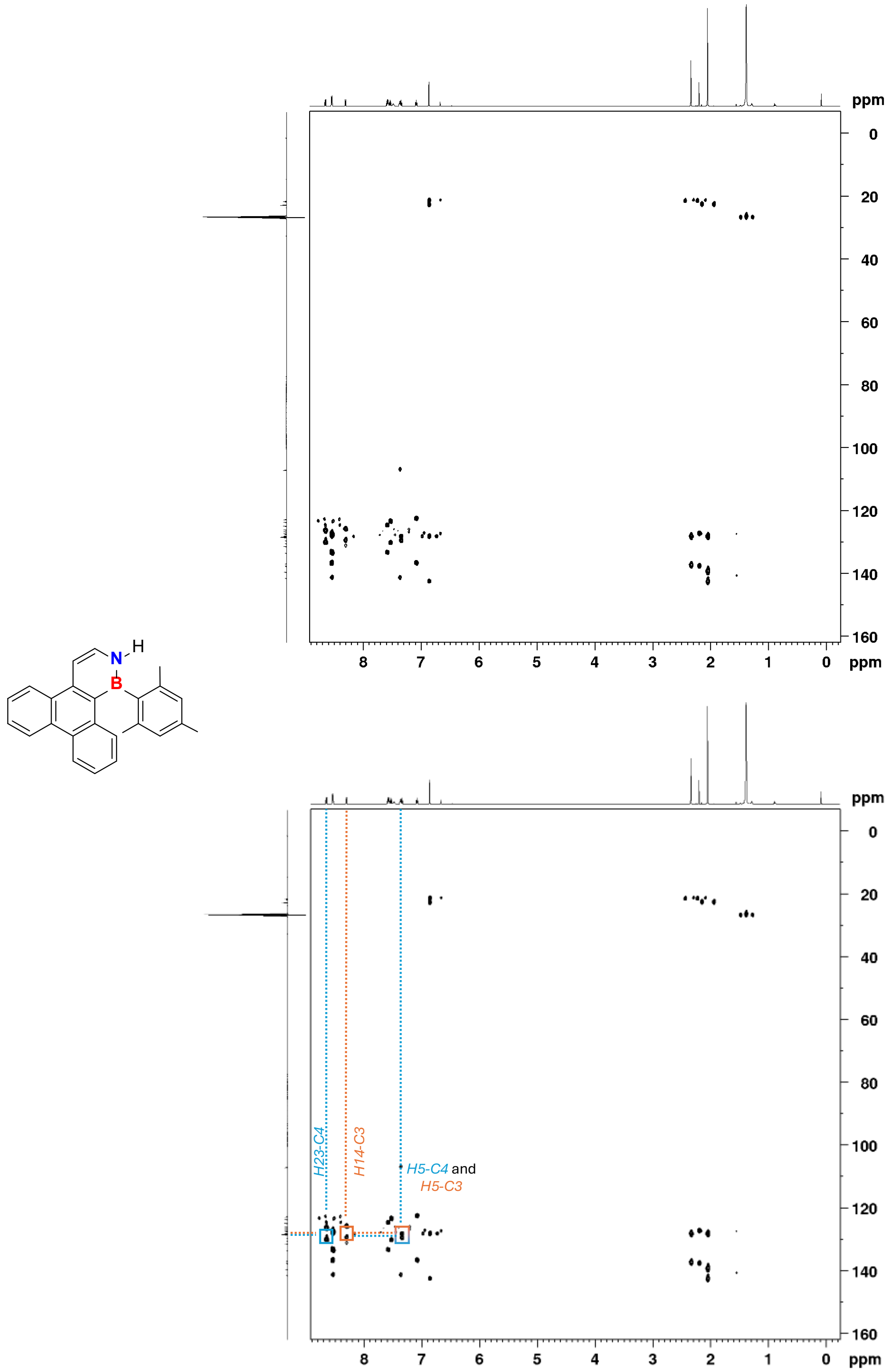

Figure S41. ^{1}H-^{13}C-HMBC NMR spectrum of compound **4** in C_6D_{12} measured on a 600 MHz spectrometer. Shown are the HMBC couplings from H-5 and H-23 to the quaternary carbon C4, and from H-5 and H-14 to the quaternary carbon C3 (bottom).

Reaction of 2 in the presence of iodine

A solution of **2** (3 mg, 6.5 µmol) in cyclohexane-d_{12} (0.5 mL) was prepared under inert atmosphere in a quartz Schlenk tube and iodine (3.3 mg, 13 µmol) was added.

Procedure A. The solution was irradiated for 5 min with a wavelength range of 280-400 nm while cooled with compressed air (T = 30-35 °C). The crude product was purified by column chromatography (*n*-hexane/DCM 80:20) to afford the product as a colorless oil (1.9 mg, 4.2 µmol, 64%).

Procedure B. The solution was heated to 60 °C in an oil bath for 16 h. The crude product was purified by column chromatography (*n*-hexane/DCM 80:20) to afford the product as a colorless oil (2.2 mg, 4.9 µmol, 75%).

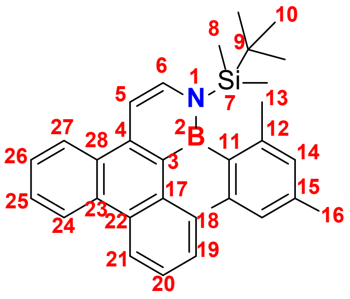

$C_{31}H_{36}BNSi$ (461.53 g/mol)

^{1}H-NMR (600 MHz, **C_6D_{12}**): δ = 8.61 (d, $^{3}J_{HH}$ = 8.20 Hz, 1H, H-27), 8.51 (d, $^{3}J_{HH}$ = 8.20 Hz, 2H, H-21/H-24), 7.89 (d, $^{3}J_{HH}$ = 8.94 Hz, 1H, H-18), 7.61 (m, 2H, H-25/H-26), 7.56 (m, 2H, H-6), 7.51 (m, 1H, H-5), 7.27 (m, 1H, H-20), 6.93 (m, 1H, H-19), 6.84 (s, 2H, H-14), 2.35 (s, 3H, H-16), 2.02 (s, 6H, H-13), 0.97 (s, 9H, H-10), 0.02 (s, 6H, H-8) ppm.

^{13}C-{^{1}H}-NMR (128 MHz, **C_6D_{12}**): δ = 143.8, 139.1, 136.8, 135.8, 132.8, 130.1, 129.2, 128.9, 127.6, 127.3, 127.2, 125.8, 125.5, 124.6, 123.9, 122.5, 121.8, 107.1, 27.5, 22.3, 20.7, 19.3, -3.3 ppm.

^{11}B-{^{1}H}-NMR (128 MHz, **C_6D_{12}**): δ = 40.8 ppm.

HRMS (ASAP) m/z: $[M + H]^+$ Calcd for $C_{31}H_{37}BNSi$ 462.2783; Found 462.2778.

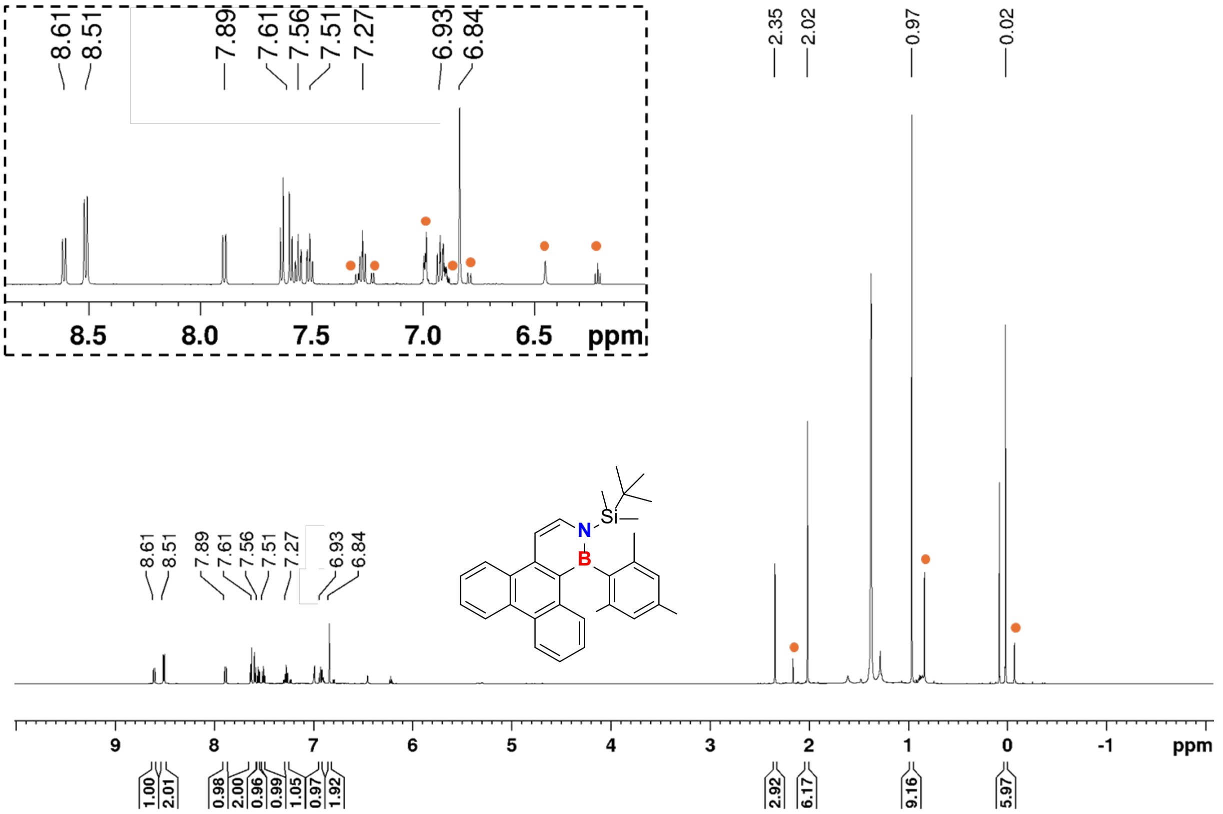

Figure S42. ^{1}H NMR spectrum of compound **5** in C_6D_{12} measured on a 600 MHz spectrometer.

*The signals marked with orange dots originate from a contamination with **^{BN}B3** (5 mass %, determined by NMR). Purification by HPLC, as described on page 21, was omitted due to the limited amount of precursor available (6 mg).*

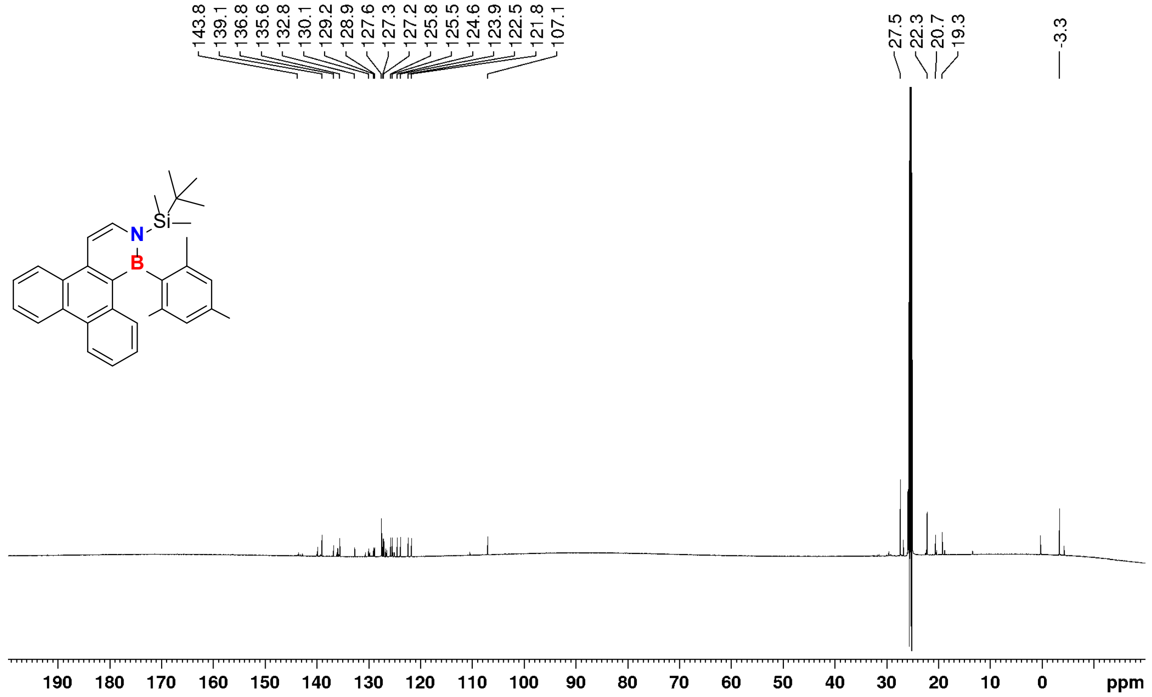

Figure S43. ^{13}C-{^{1}H} NMR spectrum of compound **5** in C_6D_{12} measured on a 600 MHz spectrometer.

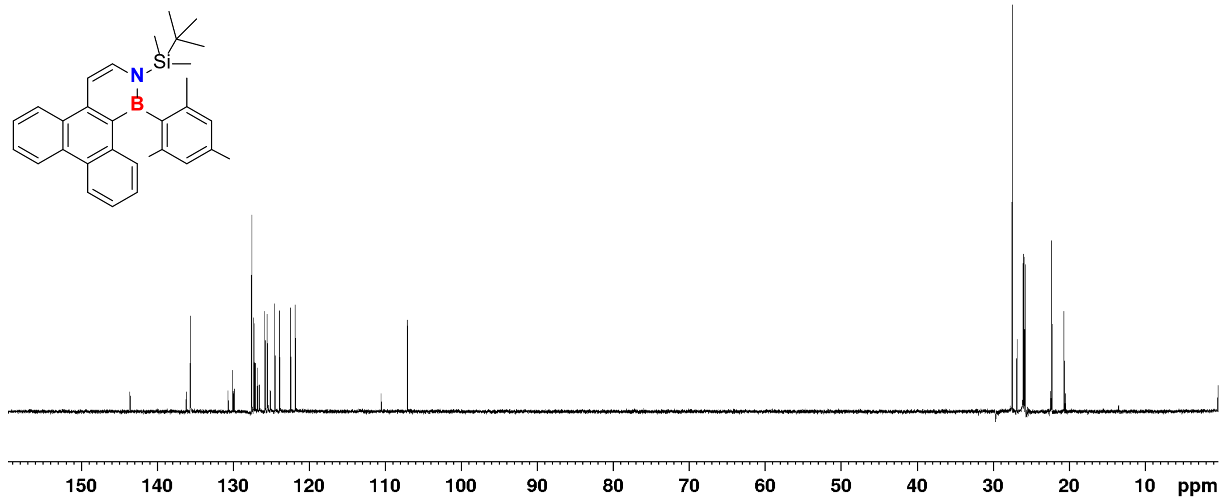

Figure S44. ^{13}C-{^{1}H}-135-DEPT NMR spectrum of compound **5** in C_6D_{12} measured on a 600 MHz spectrometer.

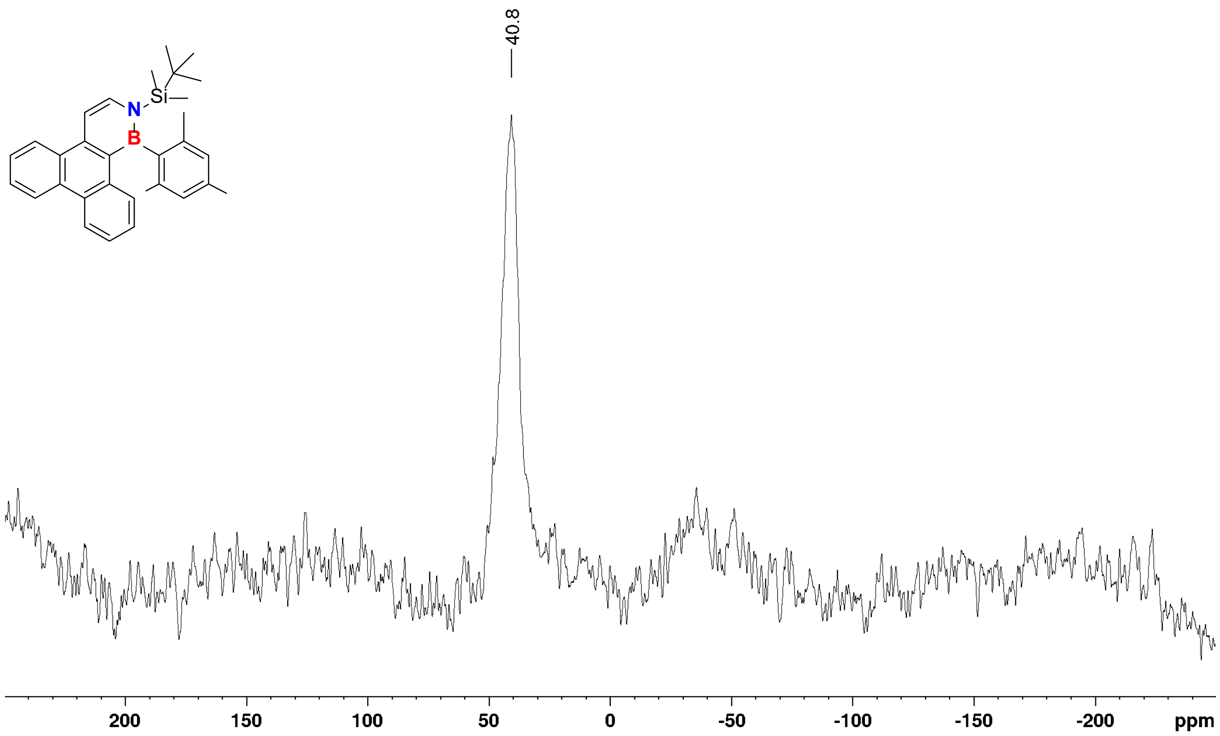

Figure S45. ^{11}B-{^{1}H} NMR spectrum of compound **5** in C_6D_{12} measured on a 400 MHz spectrometer.

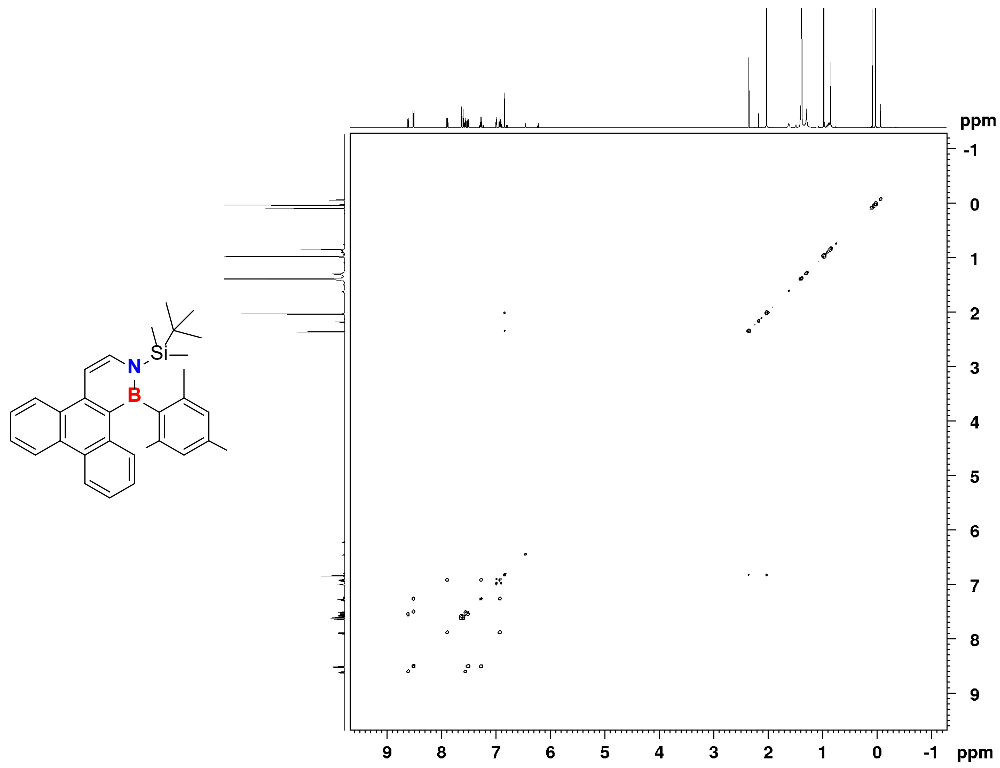

Figure S46. ^{1}H-^{1}H-COSY NMR spectrum of compound **5** in C_6D_{12} measured on a 600 MHz spectrometer.

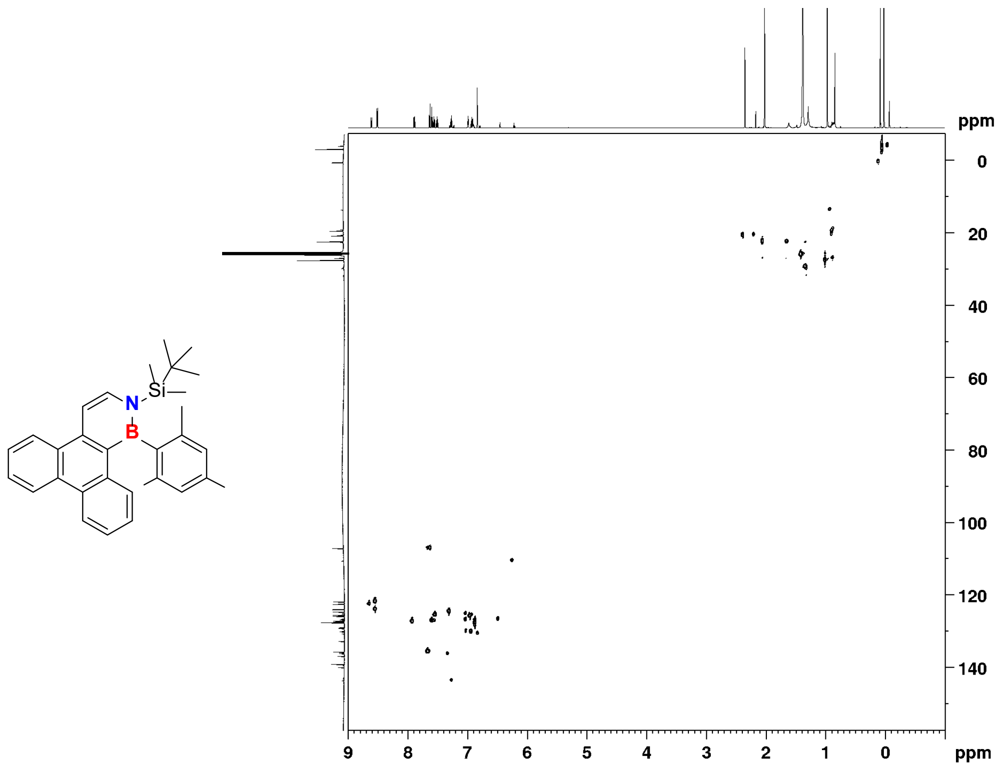

Figure S47. ^{1}H-^{13}C-HSQC NMR spectrum of compound **5** in C_6D_{12} measured on a 600 MHz spectrometer.

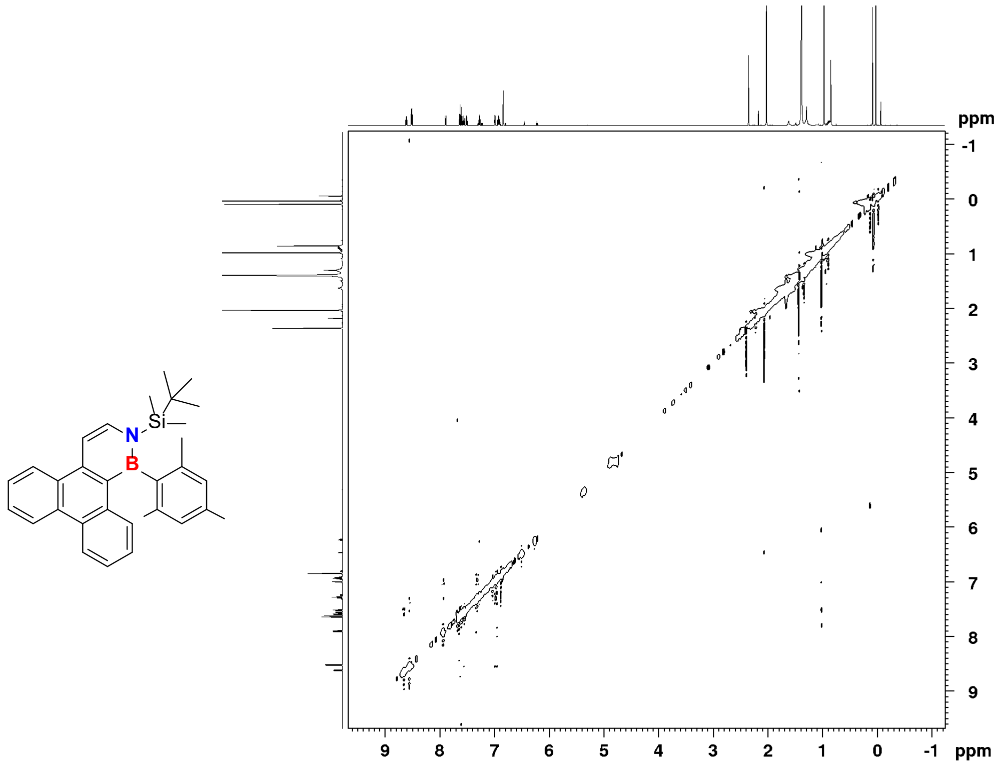

Figure S48. ^{1}H-^{1}H NOESY NMR spectrum of compound **5** in C_6D_{12} measured on a 600 MHz spectrometer.

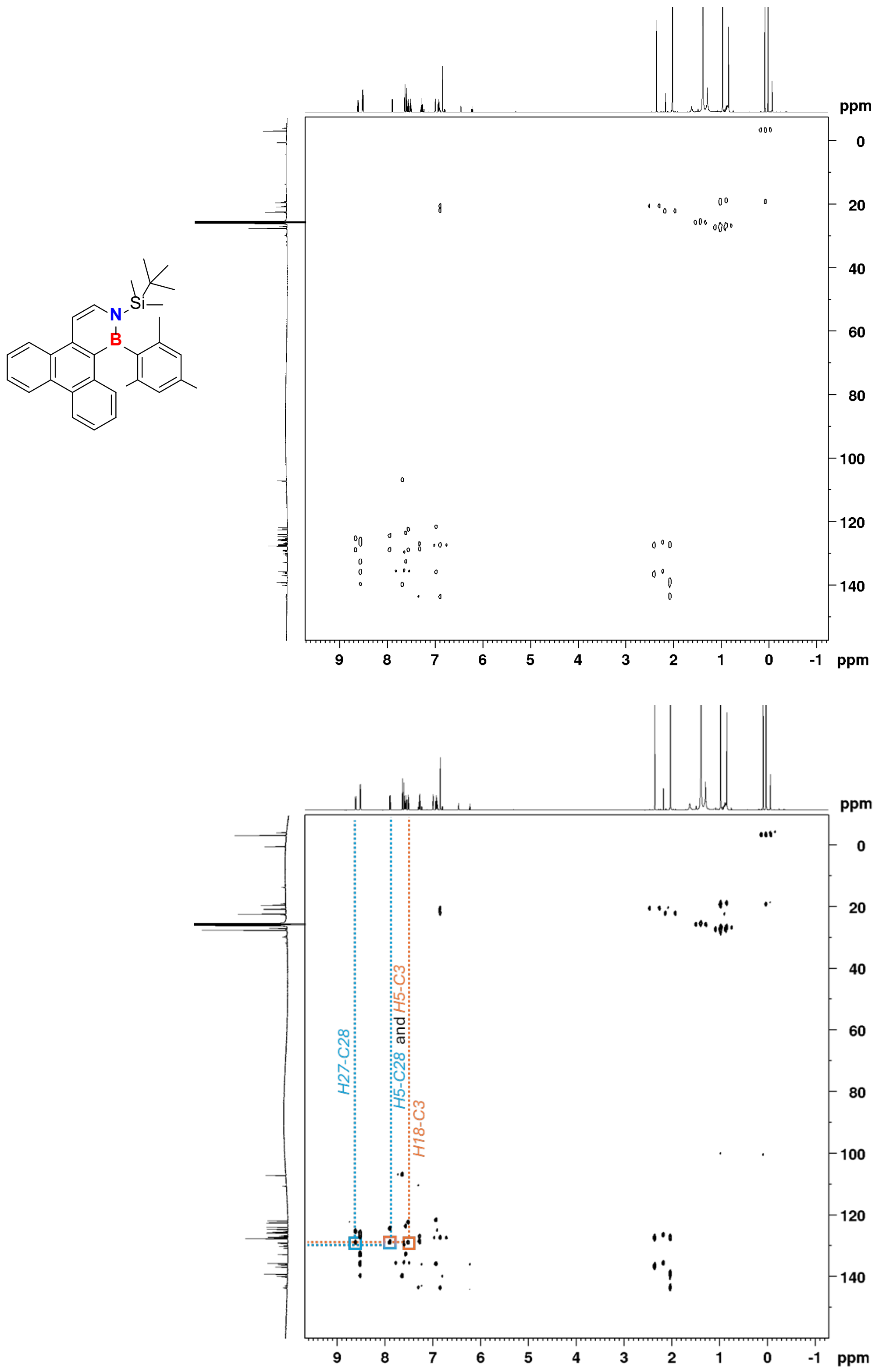

Figure S49. ^{1}H-^{13}C-HMBC NMR spectrum of compound **5** in C_6D_{12} measured on a 600 MHz spectrometer. Shown are the HMBC couplings from H-5 and H-27 to the quaternary carbon C28, and from H-5 and H-18 to the quaternary carbon C3 (bottom).

Low-temperature UV-Vis irradiation experiment with ^{BN}B5

A UV-Vis sample of ^{BN}**B5** in *n*-hexane was irradiated at 0 °C. As shown in Figure S50, the absorption band of ^{BN}**B5** vanishes under these conditions, supporting the assumption that a photoisomerization of such compounds is in principle possible. As all potential photoisomers absorb considerably short wavelengths and are concealed by solvent absorption, the nature of the photoisomerization product cannot be conclusively determined. Upon heating to 45 °C, the ^{BN}**B5** absorption regains intensity, accompanied by the emergence of a second, broad absorption band at around 400 nm. This finding suggests that a decomposition of the unknown photoproduct occurs parallel to its thermal reversion. Accordingly, the kinetic and thermodynamic parameters of the thermal back-reaction could not be determined.

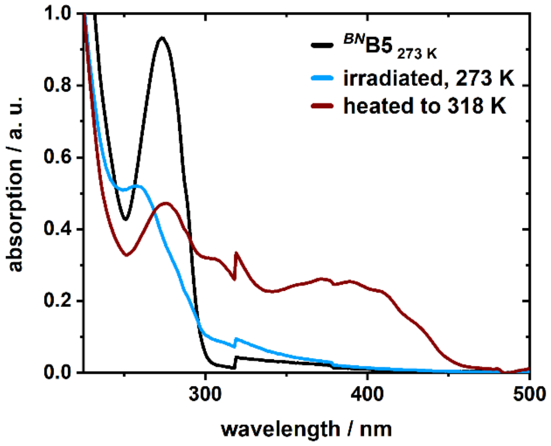

Figure S50. Comparison of the UV-Vis spectra of ^{BN}**B5** at 0 °C (273 K) before (black) and after (blue) irradiation as well as after heating to 45 °C (318 K, red).

5. Crystal structures

Refinement details

The implementation NoSpherA2 for non-spherical atom form factors in *Olex2* makes use of tailor made aspherical atomic form factors calculated from a Hirshfeld-partitioned electron density (ED), not from spherical atom form factors.[10] The electron density is calculated from a Gaussian basis set single determinant SCF wavefunction for a fragment of the crystal. This fragment can be embedded in an electrostatic crystal field by employing cluster charges or modelled using implicit solvation models, depending on the software used. This was computed using B3LYP/6-31G(d,p),[11-14] normal integration accuracy, a charge of 0 and a multiplicity of 1 with Orca 5.0.[15]

Table S1. Parameters and results of the X-ray diffraction measurement of compound **4**.

Parameter	unit	4
Molecular formula		$C_{25}H_{22}BN$
CCDC		2498872
$D_{calc.}$	g cm^{-3}	1.234
μ	mm^{-1}	0.530
Molar weight	g/mol	347.284
Colour		clear colourless
Shape		block-shaped
Size	mm^3	0.16×0.15×0.12
T/K		149.99(10)
Crystal System		orthorhombic
Space Group		*Pbcn*
a/Å		17.9893(1)
b/Å		11.5834(1)
c/Å		17.9378(1)
α/°		90
β/°		90
γ/°		90
V	Å^3	3737.83(4)
Z		8
Z'		1
Wavelength	Å	1.54184
Radiation type		Cu K$_a$
Θ_{min}	°	4.54
Θ_{max}	°	79.86
Measured Refl's.		-22 ≥ h ≥ 22
Indep't Refl's		-12 ≥ k ≥ 14
Refl's I≥2 σ(I)		-22 ≥ l ≥ 22
R_{int}		158832
Parameters		4072
Restraints		3983
Largest Peak		0.0174
Deepest Hole		443
GooF		0
wR_2 (all data)		0.0365
wR_2		0.0362
R_1 (all data)		0.0153
R_1		0.0149

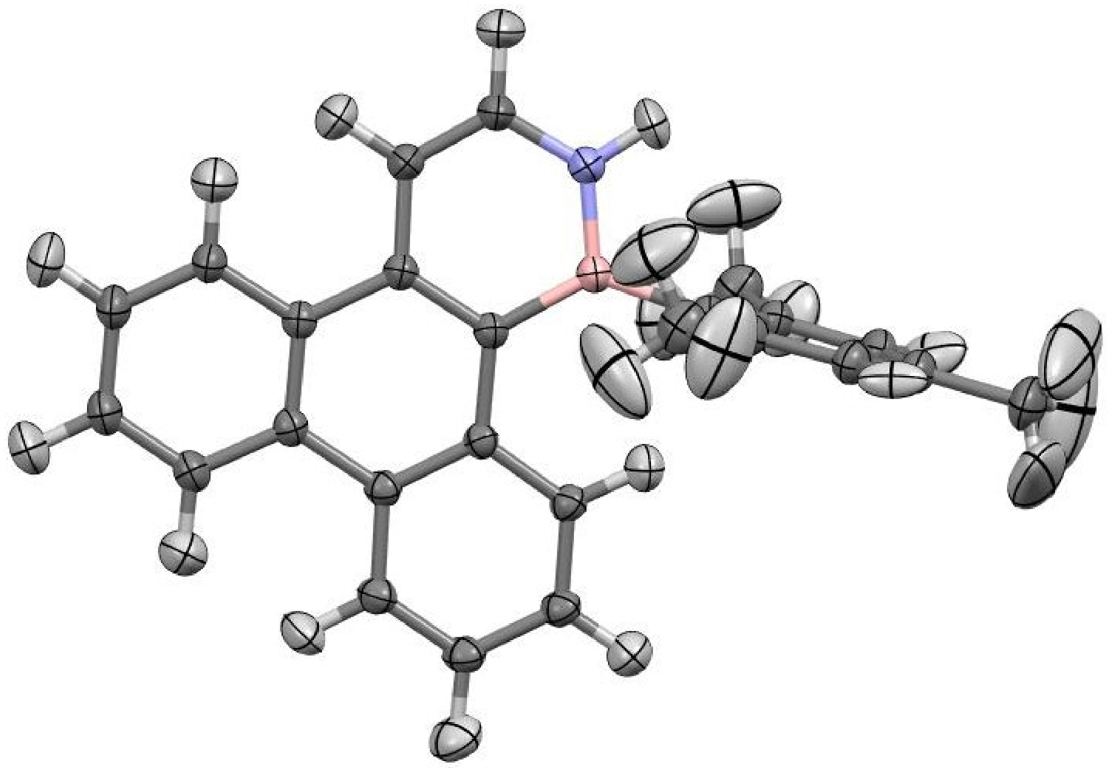

Figure S51. Crystal structure of **4**. Thermal ellipsoids are drawn at the 50% probability level.

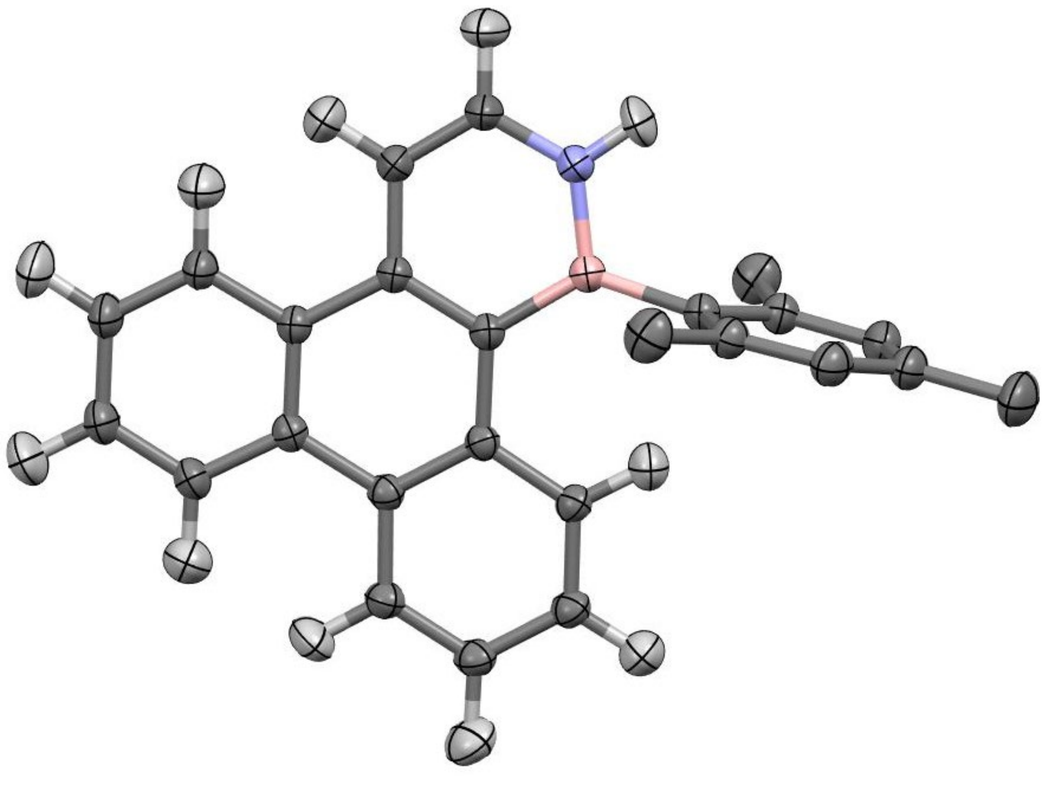

Figure S52. Crystal structure of **4**. Hydrogens of the mesityl group are omitted for clarity and thermal ellipsoids are drawn at the 50% probability level.

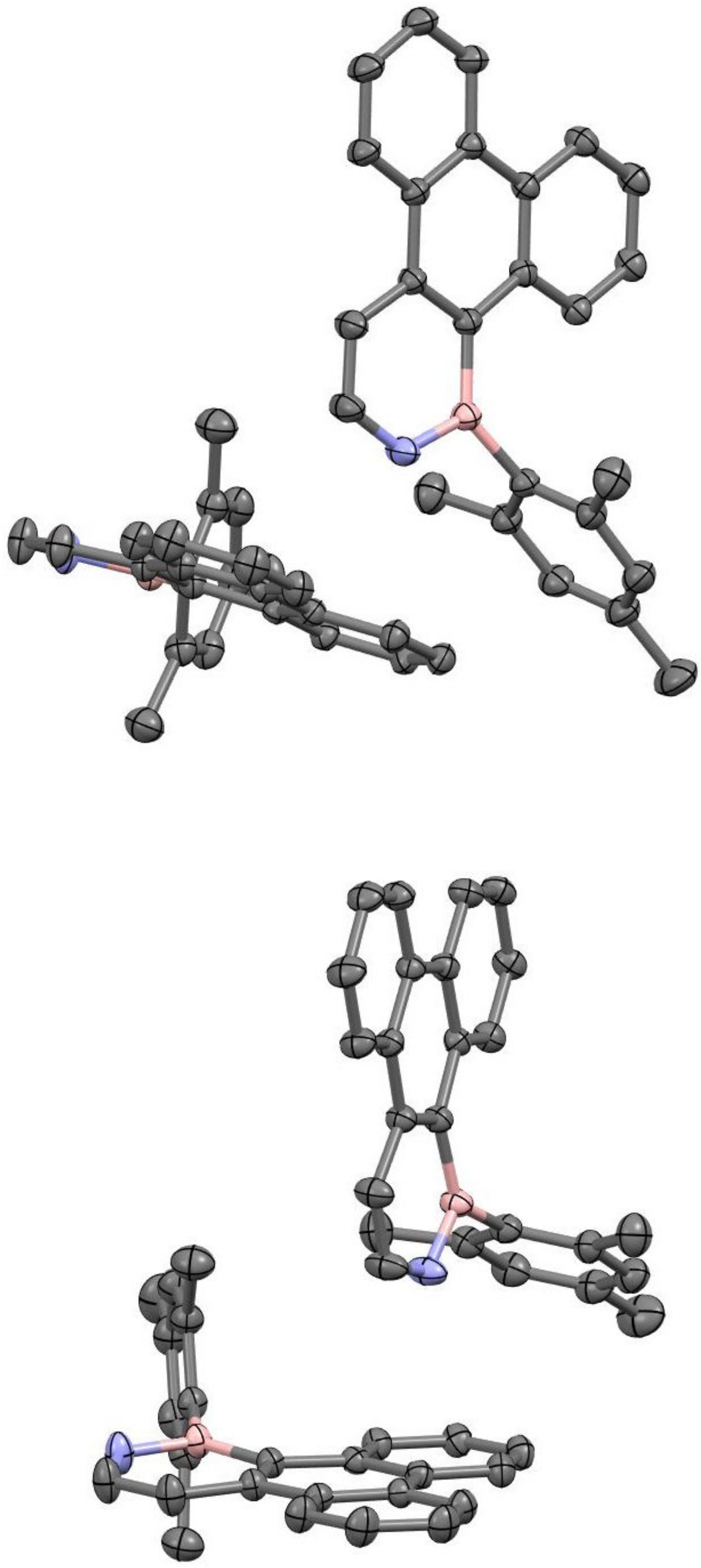

Figure S53. Section of the crystal packing highlighting the interaction between the NH group and the aromatic framework of an adjacent molecule viewed from two different angles.

6. Computations

Electronic structure computations: All geometries were optimized using the M06-2X functional[11] and the 6-311+G(d,p) basis set[16] in Gaussian 16.[17] The following coordinates are given in Å. Verification of the stationary points involved analytical frequency computations, confirming minima with zero imaginary vibrational frequencies and one imaginary vibrational frequency for transition states.

For a verification of the transitions states intrinsic reaction coordinates were calculated by using the local quadratic approximation.

Free energies were computed using standard thermochemistry equations implemented in Gaussian and are given at 298.15 K and 1 atm. In this section, total energies are given in Hartree/particle and kcal/mol.

Relative energies of the optimized structures (M06-2X/6-311+G(d,p))

Table S2. Zero-point vibrational energy (ZPVE) and Gibbs energy (G) of the optimized structures that occur during the thermal [1,5]-H-Shift from **1** to **2**. The relative Gibbs energy is given with respect to ***BN*B3**.

	ZPVE		Gibbs Energie (G)		$\Delta G^{\ddagger}$	G_{rel}
	hartree/particle	kcal/mol	hartree/particle	kcal/mol	kcal/mol	kcal/mol
***BN*B3**	-1572.56	-986792.79	-1572.62	-986834.54		0.00
**1**	-1572.47	-986740.61	-1572.54	-986781.54		53.00
**TS**	-1572.46	-986732.37	-1572.52	-986773.02	**8.52**	61.52
**2**	-1572.55	-986788.21	-1572.61	-986829.56		4.98

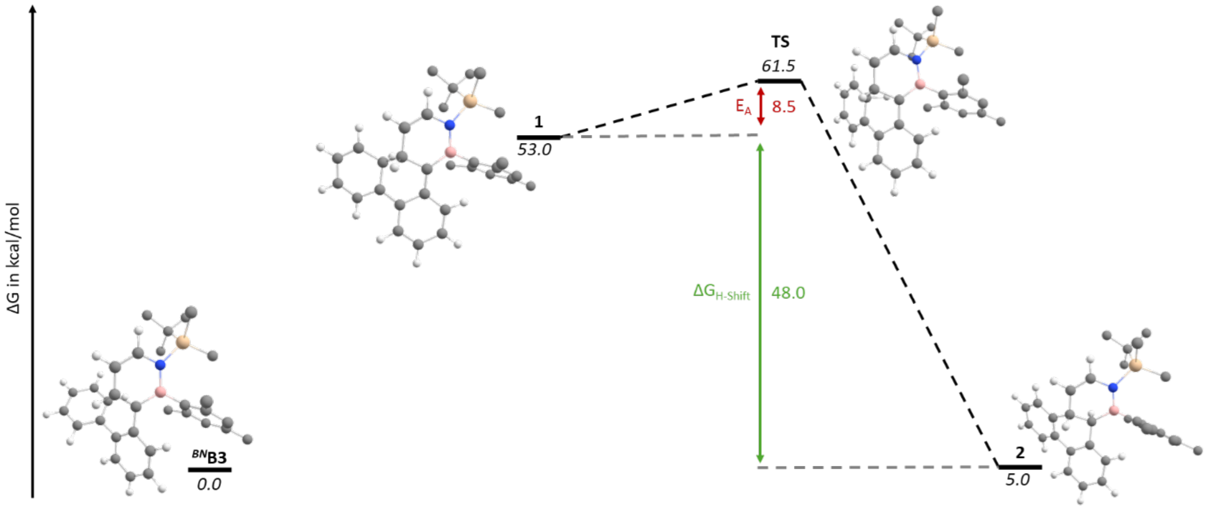

Figure S54. Mechanism calculated at the M062X/6-311+G(d,p) level of theory for the thermally allowed [1,5]-H shift forming compound **2**, with Gibbs energies (298.15 K) relative to ***BN*B3**.

Cartesian coordinates

Compound **BNB3**

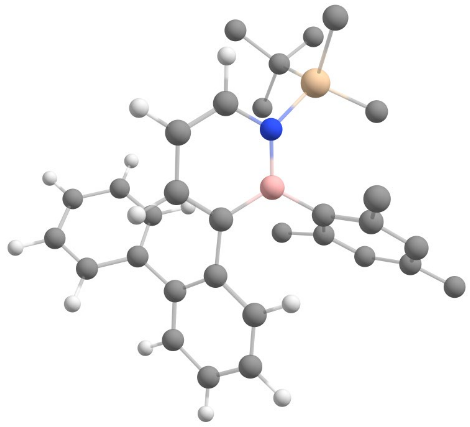

C	1.57219000	-0.92904000	1.85660200
C	1.09192100	0.11720000	1.11201700
C	-0.54350400	-2.07866200	1.81203100
C	0.74797500	-2.01964500	2.23350300
H	2.61237800	-0.94142200	2.17414700
H	-1.15473800	-2.93327000	2.08088600
H	1.14058200	-2.82337100	2.84182700
C	-1.11592900	1.36391400	0.09864700
C	-1.95414000	2.12324900	0.93924100
C	-0.88556000	1.82191000	-1.20750400
C	-2.57295100	3.27372500	0.45796900
C	-1.53583700	2.96723000	-1.67103000
C	-2.39023300	3.70268400	-0.85623100
H	-3.21285900	3.85105000	1.12023900
H	-1.35698200	3.30088400	-2.68963900
C	0.10111000	1.12818700	-2.11401900
H	1.12024200	1.45779900	-1.88323200
H	0.06860400	0.04511300	-1.99020600
H	-0.09608500	1.35622300	-3.16314200

C	-2.18303700	1.70715700	2.37520400
H	-2.79896600	0.80404900	2.43898800
H	-1.23930200	1.47672600	2.87869400
H	-2.68720300	2.49638200	2.93473400
C	-3.10734700	4.92127300	-1.37721600
H	-2.57477800	5.36126300	-2.22170400
H	-4.11397000	4.66134300	-1.71692700
H	-3.20762500	5.68141000	-0.60025700
C	-3.77455800	-0.19502900	-0.32973600
H	-3.99758500	0.57762200	0.40660800
H	-3.31267900	0.29233400	-1.18986300
H	-4.72525700	-0.63549600	-0.64711900
C	-3.74249400	-2.33211400	1.80551100
H	-3.36082400	-3.28820800	2.16733700
H	-3.76959900	-1.63992300	2.65175800
H	-4.77416900	-2.49237400	1.47995800
C	-2.42319100	-2.88420600	-0.97016900
C	-1.41685100	-2.31598000	-1.97946100
H	-1.27298300	-3.02124500	-2.80661400
H	-1.75797800	-1.36797500	-2.40780000
H	-0.44239500	-2.14890500	-1.50889800
C	-1.86528100	-4.20014900	-0.40872000
H	-2.53161500	-4.64518300	0.33640100
H	-1.75364900	-4.92673100	-1.22220100
H	-0.87942800	-4.06570000	0.04325100
C	-3.74920900	-3.18207100	-1.69077900
H	-4.13929400	-2.30055400	-2.20538200
H	-3.59374300	-3.96257300	-2.44422400
H	-4.51879300	-3.54298100	-1.00030100
C	2.02011200	1.21433900	0.72093800
C	1.66223700	2.54186800	0.98721300
C	3.26342800	0.96842000	0.09922300

C	2.49380900	3.60328200	0.65727500
H	0.71287600	2.73891200	1.47071900
C	4.09271300	2.04784200	-0.22415300
C	3.71768400	3.35685600	0.04530300
H	2.18532800	4.61817000	0.87871600
H	5.03607500	1.84698700	-0.72033200
H	4.37289500	4.17611500	-0.22596400
N	-1.14280000	-1.09745100	1.05419600
B	-0.39100100	0.10083000	0.72149700
Si	-2.75667400	-1.58883400	0.38761200
C	3.73814600	-0.39863100	-0.26072100
C	5.00742400	-0.82497700	0.13663000
C	2.95236300	-1.26624600	-1.02484500
C	5.47614300	-2.08901300	-0.20998000
H	5.62326000	-0.16404000	0.73727300
C	3.41942300	-2.52567800	-1.37608800
H	1.96753500	-0.94446100	-1.34091600
C	4.68308400	-2.94434000	-0.96604100
H	6.46032400	-2.40546500	0.11540900
H	2.79665600	-3.18206300	-1.97312600
H	5.04594600	-3.92890000	-1.23619700

Compound **1**

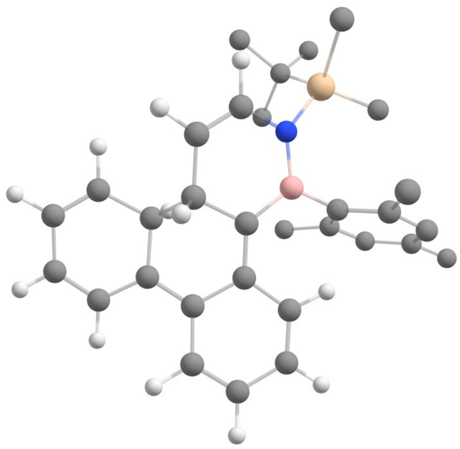

C	-2.02034100	1.11323300	1.02072300
C	-1.24395300	-0.15796700	0.69606800
C	0.19055400	2.24427600	1.29289800
C	-1.13890700	2.27488800	1.35501400
H	-2.63811600	0.90754900	1.91598800
H	0.74306700	3.11899600	1.61859800
H	-1.60003500	3.17027100	1.75499700
C	1.19836600	-1.33179300	0.14417600
C	1.90996400	-2.05709300	1.11763200
C	1.25465700	-1.75405900	-1.19134900
C	2.69007100	-3.14641000	0.74383600
C	2.06600700	-2.83573200	-1.54290200
C	2.79750400	-3.53916600	-0.59135400
H	3.23243300	-3.70047500	1.50553400
H	2.11149300	-3.14630000	-2.58348500
C	0.38011900	-1.12600800	-2.25041100
H	-0.56592500	-1.67450800	-2.31772800
H	0.13522800	-0.08915100	-2.02096500
H	0.85828800	-1.15929400	-3.23177200
C	1.81249400	-1.66035000	2.57172300

H	2.25638900	-0.67477600	2.74566400
H	0.76673400	-1.59584900	2.88861800
H	2.32039300	-2.38105400	3.21401200
C	3.69007600	-4.68754900	-0.98548400
H	3.39334400	-5.10562600	-1.94848400
H	4.72913500	-4.35710500	-1.07069300
H	3.65802300	-5.48446800	-0.24009100
C	3.88185800	0.23396300	0.21713900
H	3.94850100	-0.43573300	1.07506000
H	3.65905900	-0.37120400	-0.66342700
H	4.86573900	0.69485100	0.08381300
C	3.31404200	2.54713800	2.03928400
H	2.81663200	3.49633400	2.24419600
H	3.20722000	1.91511600	2.92565900
H	4.38036100	2.75678000	1.91370900
C	2.69036600	2.79457700	-1.00123300
C	1.88203400	2.14836600	-2.13342200
H	1.95713300	2.75251300	-3.04565200
H	2.24225700	1.14139300	-2.36990100
H	0.82360700	2.07570700	-1.86543800
C	2.09703300	4.17880100	-0.69837500
H	2.62662200	4.68173800	0.11641500
H	2.18164900	4.81644100	-1.58667800
H	1.03747500	4.12091500	-0.43899500
C	4.14447600	2.98333700	-1.46561900
H	4.58406600	2.04623900	-1.81570200
H	4.17733400	3.69588000	-2.29796900
H	4.78156500	3.38172100	-0.66875700
C	-2.04179700	-1.24949800	0.40678200
C	-1.56192200	-2.62199800	0.36859800
C	-3.48765800	-1.06648400	0.15420500
C	-2.40321500	-3.67271300	0.24962300

H	-0.51055200	-2.81072100	0.51813900
C	-4.33998000	-2.23740700	0.10157400
C	-3.82698400	-3.48416000	0.14125000
H	-2.00501600	-4.68049200	0.27719800
H	-5.41215600	-2.10515000	0.04257800
H	-4.47957800	-4.34823900	0.10504700
N	0.97849500	1.15564700	0.84692000
B	0.31611700	-0.09742500	0.59211000
Si	2.67935000	1.63635000	0.51753500
C	-3.96439000	0.18677600	-0.16581200
C	-5.29866400	0.43370000	-0.66451000
C	-3.01162800	1.35894600	-0.11170200
C	-5.76939000	1.69030000	-0.81742300
H	-5.94021200	-0.40155900	-0.91143200
C	-3.68884000	2.70036600	-0.09936900
H	-2.42374300	1.32634500	-1.06026900
C	-4.96659900	2.85085000	-0.46441100
H	-6.77583600	1.84553600	-1.18891600
H	-3.07645900	3.56467600	0.12975100
H	-5.41918400	3.83463200	-0.50598400

TS-[1,5]-H-Shift (**1** → **2**)

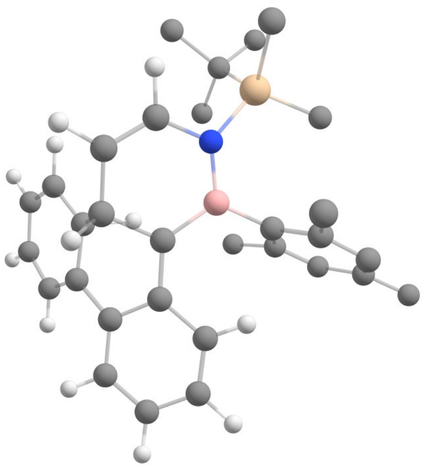

C	-1.75615500	1.27412800	1.50699000
C	-1.19345200	-0.03366900	0.94245700
C	0.61997200	2.00964200	1.71291300
C	-0.67785800	2.21391200	1.94404900
H	-2.45269800	1.09137700	2.33824500
H	1.33131400	2.72964300	2.10551200
H	-0.96040800	3.08871600	2.51921100
C	0.90949800	-1.52710900	0.08092100
C	1.62842200	-2.39613500	0.92246600
C	0.65656700	-1.92234500	-1.24227500
C	2.10435500	-3.60943200	0.43256300
C	1.16344800	-3.13530600	-1.71192300
C	1.89374900	-3.99049600	-0.89210400
H	2.65192500	-4.27367800	1.09600200
H	0.97026700	-3.42567000	-2.74118000
C	-0.20184800	-1.08711700	-2.16308900
H	-0.01495600	-1.34010800	-3.20830000
H	-1.26285500	-1.26722000	-1.95716000
H	-0.02274300	-0.01893500	-2.03193600
C	1.87597900	-2.02571600	2.36662500

H	2.27427500	-2.87283200	2.92676400
H	2.58826700	-1.19866700	2.44857400
H	0.95351500	-1.69544500	2.85412000
C	2.45623400	-5.28384400	-1.42269200
H	3.48019500	-5.14143200	-1.77974600
H	2.48089100	-6.05066300	-0.64639400
H	1.86272100	-5.65897600	-2.25791700
C	3.70218900	-0.22964300	-0.34606000
H	3.79597400	-1.05809700	0.35686200
H	3.20837300	-0.61188100	-1.24148300
H	4.71228700	0.09286400	-0.61733700
C	3.89407100	1.82087800	1.86476300
H	3.54553600	2.74080500	2.33732800
H	3.93092300	1.04354300	2.63298700
H	4.91947900	1.99556900	1.52581800
C	2.72972200	2.63389400	-0.90129600
C	1.63700900	2.27457900	-1.91681600
H	1.58971100	3.03248200	-2.70808600
H	1.83222200	1.30951900	-2.39561500
H	0.65178900	2.22617400	-1.43992100
C	2.40549400	4.00565700	-0.29030800
H	3.13741400	4.29969000	0.46817300
H	2.42238100	4.77087100	-1.07585800
H	1.41160100	4.02568900	0.16292300
C	4.07911600	2.73751800	-1.63191200
H	4.31591700	1.82293500	-2.18117300
H	4.04711000	3.55955400	-2.35650200
H	4.90396300	2.94288200	-0.94142400
C	-2.21330400	-1.00564100	0.71086600
C	-2.00376900	-2.41581600	0.75808700
C	-3.53598100	-0.54622400	0.37375100
C	-3.04145600	-3.29315500	0.59492000

H	-1.01810400	-2.79243400	0.99506200
C	-4.59063000	-1.48703400	0.22236400
C	-4.35523500	-2.82767800	0.34290700
H	-2.86071600	-4.35755700	0.68974700
H	-5.59588700	-1.13147000	0.02878200
H	-5.16991100	-3.53574000	0.24940300
N	1.19053500	0.92031300	1.00966200
B	0.32689300	-0.19289900	0.69459600
Si	2.84319500	1.24993800	0.41138600
C	-3.64328800	0.81084000	-0.04299500
C	-4.64180500	1.28764700	-0.92948200
C	-2.51823100	1.68625800	0.26222700
C	-4.58319500	2.55482300	-1.44412600
H	-5.44983900	0.62895600	-1.22337100
C	-2.55758300	3.05360400	-0.22821800
H	-1.62660700	1.01715300	-0.37683600
C	-3.52825900	3.44754400	-1.09206400
H	-5.34975900	2.88838700	-2.13358100
H	-1.76472800	3.72773200	0.07587600
H	-3.52443300	4.45206800	-1.49862600

Compound **2**

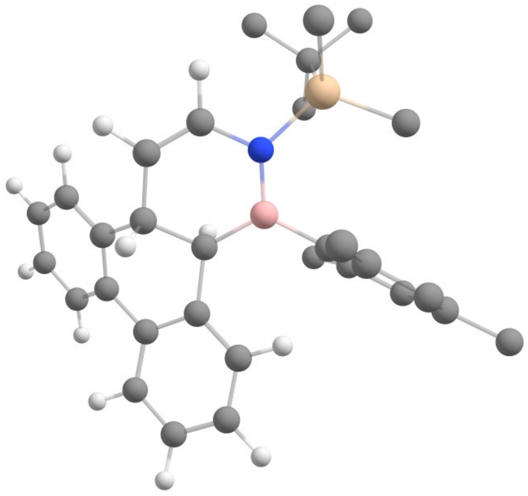

C	-1.97785100	-1.03776900	-1.08637000
C	-1.24364700	0.01251900	-0.22058200
C	0.21019100	-2.21833200	-1.27209700
C	-1.11202900	-2.20934300	-1.42673200
H	-2.25703300	-0.54429900	-2.03223900
H	0.76298200	-3.09376700	-1.59469300
H	-1.56294400	-3.07070800	-1.90494700
C	1.19893300	1.33973500	-0.03600800
C	1.82926900	2.03331400	-1.08451300
C	1.28313800	1.85586200	1.26636900
C	2.56586500	3.18394000	-0.81472100
C	2.05017700	2.99491700	1.51150400
C	2.70710500	3.66854300	0.48467400
H	3.04202300	3.71524500	-1.63442200
H	2.11985100	3.37773800	2.52601200
C	0.49418300	1.24015000	2.39668300
H	0.86034700	1.58188000	3.36593800
H	-0.55952000	1.52859300	2.31469900
H	0.54012700	0.14994300	2.38353700

C	1.69061200	1.55278900	-2.51108200
H	2.05830000	2.30178000	-3.21371200
H	2.24843900	0.62632000	-2.67869700
H	0.64507400	1.34184000	-2.75799600
C	3.55278500	4.88240000	0.76975500
H	4.59214000	4.59342700	0.94982200
H	3.54359100	5.57592900	-0.07275300
H	3.19764900	5.41171600	1.65533900
C	3.89448300	-0.20609000	-0.17108800
H	3.93798800	0.49562400	-1.00461500
H	3.66569400	0.36365000	0.73108100
H	4.89025000	-0.64698900	-0.06111700
C	3.31891100	-2.47239500	-2.07304700
H	2.88266500	-3.45752400	-2.24706300
H	3.09921100	-1.85184700	-2.94656100
H	4.40417400	-2.59803800	-2.02473400
C	2.77656700	-2.82775700	0.97529400
C	2.09153400	-2.17563900	2.18330100
H	2.19277500	-2.81568900	3.06785000
H	2.52921700	-1.20177300	2.42703600
H	1.02242700	-2.03478900	1.99629300
C	2.08134800	-4.16586400	0.68247200
H	2.51076900	-4.67008400	-0.18865000
H	2.20291300	-4.83730800	1.54088400
H	1.00903200	-4.04027900	0.51614700
C	4.24872300	-3.10920700	1.32039500
H	4.76739200	-2.20834600	1.65734700
H	4.30489500	-3.84462500	2.13118200
H	4.79809100	-3.52072100	0.46710500
C	-2.11527600	1.26096900	-0.21420600
C	-1.65873200	2.56466100	-0.40977700
C	-3.50183300	1.05805700	-0.03242400

C	-2.53866700	3.64439200	-0.41889900
H	-0.60660200	2.75640600	-0.56191400
C	-4.37522600	2.14710600	-0.06038100
C	-3.90030400	3.43871100	-0.24439200
H	-2.15269500	4.64460100	-0.57727100
H	-5.44139000	1.97854600	0.03708300
H	-4.59075300	4.27345100	-0.26767000
N	1.00227800	-1.15267500	-0.76492700
B	0.34403300	0.05900800	-0.38536200
Si	2.71821300	-1.62362200	-0.50367700
C	-4.01026600	-0.31917000	0.16147500
C	-5.18527400	-0.58119900	0.86838600
C	-3.27641100	-1.39065600	-0.37955200
C	-5.65056900	-1.88179600	1.01990500
H	-5.72957600	0.23718900	1.32510700
C	-3.74854000	-2.68972300	-0.21698200
H	-1.28863100	-0.39531800	0.81127900
C	-4.93260000	-2.93752700	0.47285100
H	-6.56173100	-2.06938800	1.57533400
H	-3.18615800	-3.52544100	-0.61329800
H	-5.28167400	-3.95613800	0.59478400

Intrinsic Reaction Coordinate

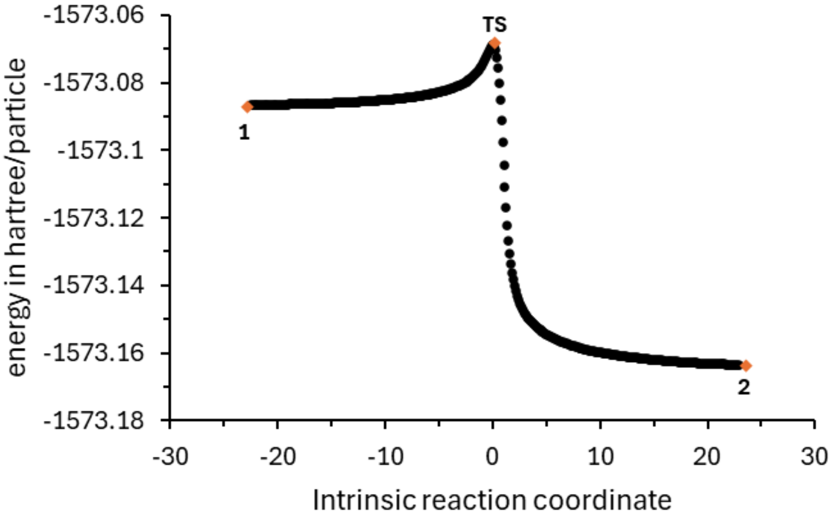

Figure S55. Intrinsic reaction coordinate of the thermal [1,5]-H-Shift from compound **1** to species **2**.

(1) Sheldrick, G. M. Acta Crystallogr. Sect. A **2008**, *2008*, 112-122.
(2) Dittrich, C. B. H. G. M. S. B. ShelXle: a Qt graphical user interface for SHELXL. J. Appl. Crystallogr. **2011**, *44*,
(3) Sheldrick, G. Crystal structure refinement with SHELXL. Acta Crystallogr. Sect. C **2015**, *71* (1), 3-8.
(4) Dolomanov, O.; Bourhis, L.; Gildea, R.; Howard, J.; Puschmann, H. OLEX2: A complete structure solution, refinement and analysis program. J. Appl. Cryst. J. Appl. Cryst **2009**, *42*, 339-341.
(5) Bourhis, L. J.; Dolomanov, O. V.; Gildea, R. J.; Howard, J. A.; Puschmann, H. The anatomy of a comprehensive constrained, restrained refinement program for the modern computing environment - Olex2 dissected. Acta Crystallogr. A Found Adv. **2015**, *71* (Pt 1), 59-75.
(6) Richter, R. C.; Biebl, S. M.; Einholz, R.; Walz, J.; Maichle-Mössmer, C.; Ströbele, M.; Bettinger, H. F.; Fleischer, I. Facile Energy Release from Substituted Dewar Isomers of 1,2-Dihydro-1,2-Azaborinines Catalyzed by Coinage Metal Lewis Acids. Angew. Chem. Int. Ed. **2024**, *63* (30), e202411078.
(7) Lee, H.; Alvarado, M.; Ingram, S.; Li, B.; Liu, S.-Y. N-Functionalization of 1,2-Azaborines. Synlett **2023**, *34* (18), 2169-2174.
(8) Biebl, S. M.; Ziemann, P.; Ströbele, M.; Bettinger, H. F. Mechanistic Insight into the Thermal Ring Opening of the Dewar Isomer of 1,2-Dihydro-1,2-azaborinines. JACS Au **2025**, *5* (10), 5006-5016.
(9) Yang, K.; Mao, Y.; Zhang, Z.; Xu, J.; Wang, H.; He, Y.; Yu, P.; Song, Q. Construction of C-B axial chirality via dynamic kinetic asymmetric cross-coupling mediated by tetracoordinate boron. Nat. Comm. **2023**, *14* (1), 4438.
(10) Kleemiss, F.; Dolomanov, O. V.; Bodensteiner, M.; Peyerimhoff, N.; Midgley, L.; Bourhis, L. J.; Genoni, A.; Malaspina, L. A.; Jayatilaka, D.; Spencer, J. L.; et al. Accurate crystal structures and chemical properties from NoSpherA2. Chem. Sci. **2021**, *12* (5), 1675-1692.
(11) Zhao, Y.; Truhlar, D. G. The M06 suite of density functionals for main group thermochemistry, thermochemical kinetics, noncovalent interactions, excited states, and transition elements: two new functionals and systematic testing of four M06-class functionals and 12 other functionals. Theor. Chem. Acc. **2008**, *120* (1), 215-241.
(12) Ditchfield, R.; Hehre, W. J.; Pople, J. A. Self-Consistent Molecular-Orbital Methods. IX. An Extended Gaussian-Type Basis for Molecular-Orbital Studies of Organic Molecules. J. Chem. Phys. **1971**, *54* (2), 724-728.
(13) Hehre, W. J.; Ditchfield, R.; Pople, J. A. Self—Consistent Molecular Orbital Methods. XII. Further Extensions of Gaussian—Type Basis Sets for Use in Molecular Orbital Studies of Organic Molecules. J. Chem. Phys. **1972**, *56* (5), 2257-2261.
(14) Francl, M. M.; Pietro, W. J.; Hehre, W. J.; Binkley, J. S.; Gordon, M. S.; DeFrees, D. J.; Pople, J. A. Self-consistent molecular orbital methods. XXIII. A polarization-type basis set for second-row elements. J. Chem. Phys. **1982**, *77* (7), 3654-3665.
(15) Neese, F. The ORCA program system. WIREs Comput. Mol. Sci. **2012**, *2* (1), 73-78.
(16) McLean, A. D.; Chandler, G. S. Contracted Gaussian basis sets for molecular calculations. I. Second row atoms, Z=11–18. J. Chem. Phys. **1980**, *72* (10), 5639-5648.
(17) *Gaussian 16 Rev. C.01*; Wallingford, CT, 2016. (accessed.
